# Proteome-wide analysis reveals an age-associated cellular phenotype of *in situ* aged human fibroblasts

**DOI:** 10.18632/aging.100698

**Published:** 2014-11-02

**Authors:** Daniel M. Waldera-Lupa, Faiza Kalfalah, Ana-Maria Florea, Steffen Sass, Fabian Kruse, Vera Rieder, Julia Tigges, Ellen Fritsche, Jean Krutmann, Hauke Busch, Melanie Boerries, Helmut E. Meyer, Fritz Boege, Fabian Theis, Guido Reifenberger, Kai Stuhler

**Affiliations:** ^1^ Institute for Molecular Medicine, Heinrich-Heine-University, Düsseldorf, Germany; ^2^ Molecular Proteomics Laboratory, Biomedical Research Centre (BMFZ), Heinrich-Heine-University, Düsseldorf, Germany; ^3^ Institute of Clinical Chemistry and Laboratory Diagnostics, Heinrich-Heine-University, Med. Faculty, Düsseldorf, Germany; ^4^ Department of Neuropathology, Heinrich-Heine-University, Düsseldorf, and German Cancer Consortium (DKTK), German Cancer Research Center (DKFZ), Heidelberg, Germany; ^5^ Institute of Computational Biology, Helmholtz Center Munich, German Research Center for Environmental Health, Neuherberg, Germany; ^6^ Leibniz Research Institute for Environmental Medicine (IUF), Düsseldorf, Germany; ^7^ Institute of Molecular Medicine and Cell Research, Albert-Ludwigs-University Freiburg, Freiburg, Germany; ^8^ German Cancer Consortium (DKTK), Freiburg, Germany; ^9^ German Cancer Research Center (DKFZ), D-69120, Heidelberg, Germany; ^10^ Department of Biomedical Research, Leibniz-Institute for Analytical Science - ISAS, Dortmund, Germany; ^11^ Department of Mathematics, Technical University Munich, Garching, Germany

**Keywords:** aging, human, stroma, dermal fibroblasts, proteomics, mass spectrometry

## Abstract

We analyzed an ex vivo model of in situ aged human dermal fibroblasts, obtained from 15 adult healthy donors from three different age groups using an unbiased quantitative proteome-wide approach applying label-free mass spectrometry. Thereby, we identified 2409 proteins, including 43 proteins with an age-associated abundance change. Most of the differentially abundant proteins have not been described in the context of fibroblasts’ aging before, but the deduced biological processes confirmed known hallmarks of aging and led to a consistent picture of eight biological categories involved in fibroblast aging, namely proteostasis, cell cycle and proliferation, development and differentiation, cell death, cell organization and cytoskeleton, response to stress, cell communication and signal transduction, as well as RNA metabolism and translation. The exhaustive analysis of protein and mRNA data revealed that 77% of the age-associated proteins were not linked to expression changes of the corresponding transcripts. This is in line with an associated miRNA study and led us to the conclusion that most of the age-associated alterations detected at the proteome level are likely caused post-transcriptionally rather than by differential gene expression. In summary, our findings led to the characterization of novel proteins potentially associated with fibroblast aging and revealed that primary cultures of in situ aged fibroblasts are characterized by moderate age-related proteomic changes comprising the multifactorial process of aging.

## INTRODUCTION

Aging is a multifactorial process that is characterized by distinct molecular and biological changes, such as increased genomic instability, telomere attrition, epigenetic alterations, loss of proteostasis, deregulated nutrient-sensing, mitochondrial dysfunction, cellular senescence and altered intercellular communication [1]. It has been suggested that the aging process of the skin is mainly determined by alterations of its dermal stroma consisting predominantly of dermal fibroblasts and the extracellular matrix [2, 3]. The dermis is a highly stationary tissue compartment of mostly quiescent cells that cannot be removed quickly in case of dysfunction. Thus, the homeostasis of the dermis is primarily based on cellular adaptation and damage clearance and thereby prone to age-related changes. As the major cell source in the dermis and a long-lived cell system, dermal fibroblasts are able to accumulate aging-associated alterations and adapt their cellular functions. Therefore, dermal fibroblasts are a favored cell model to analyse the aging process of the skin. A broadly applied fibroblast aging model is based on the limited replicative lifespan of these cells [4]. It is thought that induction of cellular senescence by telomere attrition, stress or oncogene activation is an important driver of the aging process and that accumulation of senescent cells in various organs is one of the hallmarks of aging [1]. However, it remains unclear to which extent cellular senescence induced *in vitro* is a representative model for cell and organ aging *in vivo*. Moreover, most studies analyzing aging of fibroblasts *in vivo* and *in vitro* focused on single genes or pathways [4], while comprehensive investigations of aging-associated alterations at the transcriptome-, miRNAome- and proteome-wide levels are sparse. In previous transcriptome-wide studies, replicative senescent [5] and photo-aged fibroblasts [6] were analyzed. These approaches revealed activation of the p53/p21 and p16(INK4a)/pRb pathways, differential expression of interleukins and differential expression of matrix metalloproteinases and their inhibitory proteins. There is also evidence that miRNA alterations are involved in the aging process. It was found that overexpression of the two miRNAs miR-152 and miR-181a is sufficient to induce senescence in fibroblasts [7]. At the protein level, most studies have analyzed *in vitro* aging models of fibroblasts [4]. A proteome-wide study of *in situ* aged fibroblasts was reported by Boraldi et al. who used two-dimensional gel electrophoresis coupled with MALDI-MS to study fibroblasts which were isolated from donors of various age and cultured *ex vivo* [8]. Altogether, these studies identified several cellular processes as being altered during aging, including proliferation, metabolism, response to stress, cytoskeletal organization, as well as protein synthesis and degradation. So far, a detailed systems biological study simultaneously analyzing aging-associated changes at the mRNA, miRNA and proteome levels has not been conducted. Here, we report on a proteome-wide study integrating transcriptome and miRNAome data of primary, *ex vivo* cultured adult human dermal fibroblasts obtained from 15 donors of different ages. By performing combined bioinformatic analyses of the distinct large-scale data sets, we aimed to characterize aging-associated molecular changes and to identify relevant biological processes and regulatory mechanisms.

## RESULTS

To reveal relevant biological processes and candidate proteins involved in the adaption of human dermal fibroblasts during the process of human skin aging, we analyzed an *ex vivo* model of *in situ* aged human dermal fibroblasts by proteome-wide approach integrating large-scale data from associated transcriptome and miRNA studies. In a previous work (Waldera-Lupa et al., unpublished data) we have shown that this model encompasses DNA-SCARS, which are neither accompanied by induced DNA double strand breaks nor decreased cell viability nor telomere shortening, and exhibit a secretory phenotype differing from the senescence-associated secretory phenotype [9] of common aging model systems.

### Proteome-wide characterization of *in situ* aged and *ex vivo* cultured dermal fibroblasts

**Figure 1:**
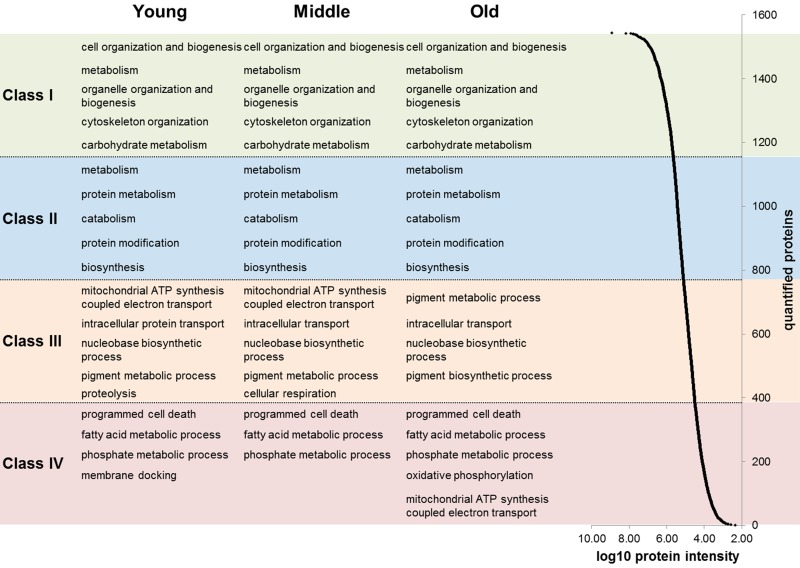
Abundance ranges of quantified proteins For each age group (young, middle aged and old) the quantified proteins were ranked into four classes based on their abundance (log10 of protein intensities). Abundances of quantified proteins spanned approximately five orders of magnitude (right). For each abundance class and age group an enrichment analysis of biological processes was applied. The five most significant biological processes are indicated in the figure.

First, we analyzed proteins expressed in *ex vivo* cultured human dermal fibroblasts by a proteomic approach using quantitative mass spectrometry (MS). Using a label-free MS approach, we identified a total of 2409 proteins analyzing 15 primary fibroblast cultures obtained from each five young (20-30), middle aged (40-50) and old (60-70) female donors. Due to high reproducibility of our label-free MS analysis we were able to consider 1607 proteins for quantitative analysis. Statistical analysis revealed 43 proteins with a significant age-associated expression (p ≤ 0.05), whereof 20 and 23 proteins exhibit positive and negative correlation with age, respectively (Tab. 1 and Tab. S1). However, principal component analysis revealed an almost homogeneous protein expression pattern of the three age groups with a low separation within the aged samples (Fig. S1). By detailed analysis of the proteome data we revealed that over 47% (755 proteins) of quantified proteins exhibited a constant abundance across all age groups and thus likely account for this homogeneous protein expression pattern (Fig. S2). By means of enrichment analysis we found that fibroblasts maintain protein abundance for biological processes such as ‘translation’, ‘metabolism of proteins’ and ‘metabolism of RNA’ during aging (Tab. S2). To reveal proteomic changes beyond the housekeeping processes, we analyzed the proteome data considering proteins’ abundances. Therefore, we ranked the quantified proteins into four abundance classes over a dynamic abundance range of approximately five orders of magnitude (Fig. 1). This approach revealed that groups of proteins linked to specific biological processes demonstrated coordinated abundance changes during aging. For instance, a group of five proteins (MTCO2, NDUFA5, NDUFA9, NDUFA10, NDUFS6) linked to the biological process ‘mitochondrial ATP synthesis coupled electron transport’ appeared among the semi-abundant proteins (class III) for the young and middle age group, but among the low-abundant proteins in the old aged donors (class IV; Tab. S3-5). For the other abundance classes (class I-II) we found no age-associated changes. Due to observed age-associated changs of mitochondrial proteins we performed a detailed analysis of proteins related to mitochondria. Cluster analysis revealed 23 age-associated mitochondrial proteins (p ≤ 0.1; 14 negative and 9 positive). Under the proteins with negative age-correlation the biological processes ‘ATP synthesis’ and ‘cellular respiration’ were significantly enriched. The biological processes ‘response to stress’ and ‘catabolism’ were enriched for proteins with a positive age-correlation (Fig. S3; Tab. S6). Proteome-wide characterization revealed that *in situ* aged fibroblasts exhibit a moderate age-associated cellular phenotype with a large number of proteins exhibiting a constant abundance across the three age groups.

**Table 1:** Significantly altered proteins with age Quantitative label-free proteome analysis of *in situ* aged fibroblasts' proteome revealed 43 proteins that are differentially altered during *in situ* aging (ANOVA: p ≤ 0.05; Pearson correlation: p ≤ 0.05). For ANOVA analysis donors were grouped according to calendar age into groups 20-30, 40-50 and 60-70 years, with five individual donors in each group.

Accession	Gene	Description	p-value	Fold change	Regulation
**P08133**	ANXA6	Annexin A6	0.001	1.5	up
**P47897**	QARS	Glutaminyl-tRNA synthetase	0.002	1.3	up
**P11717**	IGF2R	Cation-independent mannose-6-phosphate receptor	0.003	2.8	up
**Q6IBS0**	TWF2	Twinfilin-2	0.005	1.4	down
**P46782**	RPS5	40S ribosomal protein S5	0.007	2.2	down
**O43143**	DHX15	Putative pre-mRNA-splicing factor ATP-dependent RNA helicase DHX15	0.008	1.4	up
**Q99460**	PSMD1	26S proteasome non-ATPase regulatory subunit 1	0.012	1.4	down
**P62273**	RPS29	40S ribosomal protein S29	0.012	1.3	up
**Q9UMS6**	SYNPO2	Synaptopodin-2	0.012	1.8	up
**P40227**	CCT6A	T-complex protein 1 subunit zeta	0.012	1.7	down
**P08758**	ANXA5	Annexin A5	0.012	1.4	up
**Q9Y3I0**	C22orf28	tRNA-splicing ligase RtcB homolog	0.013	2.8	down
**Q13242**	SRSF9	Serine/arginine-rich splicing factor 9	0.013	1.2	up
**P60660**	MYL6	Myosin light polypeptide 6	0.017	1.2	up
**Q15435**	PPP1R7	Protein phosphatase 1 regulatory subunit 7	0.022	1.6	down
**Q9Y224**	C14orf166	UPF0568 protein C14orf166	0.022	1.9	down
**O00487**	PSMD14	26S proteasome non-ATPase regulatory subunit 14	0.022	1.4	down
**P34897**	SHMT2	Serine hydroxymethyltransferase, mitochondrial	0.022	1.7	down
**P08107**	HSPA1A	Heat shock 70 kDa protein 1A/1B	0.022	1.7	up
**Q92499**	DDX1	ATP-dependent RNA helicase DDX1	0.023	1.3	down
**P62158**	CALM1	Calmodulin	0.024	2.7	up
**O94973**	AP2A2	AP-2 complex subunit alpha-2	0.027	1.6	down
**Q92974**	ARHGEF2	Rho guanine nucleotide exchange factor 2	0.028	2.6	down
**P09525**	ANXA4	Annexin A4	0.030	1.3	up
**P27105**	STOM	Erythrocyte band 7 integral membrane protein	0.031	2.0	up
**O00571**	DDX3X	ATP-dependent RNA helicase DDX3X	0.034	1.3	down
**P04632**	CAPNS1	Calpain small subunit 1	0.034	1.6	down
**P15559**	NQO1	NAD(P)H dehydrogenase [quinone] 1	0.035	3.2	up
**Q96FQ6**	S100A16	Protein S100-A16	0.036	1.4	down
**P09497**	CLTB	Clathrin light chain B	0.037	1.5	up
**P80723**	BASP1	Brain acid soluble protein 1	0.037	1.6	up
**Q13217**	DNAJC3	DnaJ homolog subfamily C member 3	0.037	2.6	down
**O95782**	AP2A1	AP-2 complex subunit alpha-1	0.039	1.4	down
**P62753**	RPS6	40S ribosomal protein S6	0.039	1.3	up
**P41250**	GARS	Glycyl-tRNA synthetase	0.039	1.2	down
**Q9NZN4**	EHD2	EH domain-containing protein 2	0.039	1.4	up
**Q9Y3B8**	REXO2	Oligoribonuclease, mitochondrial	0.040	2.5	down
**P07996**	THBS1	Thrombospondin-1	0.044	1.5	up
**P30419**	NMT1	Glycylpeptide N-tetradecanoyltransferase 1	0.045	1.4	down
**Q01518**	CAP1	Adenylyl cyclase-associated protein 1	0.048	1.1	down
**P54652**	HSPA2	Heat shock-related 70 kDa protein 2	0.048	1.5	up
**Q96QV6**	HIST1H2AA	Histone H2A type 1-A	0.049	3.8	down
**P17987**	TCP1	T-complex protein 1 subunit alpha	0.050	1.2	down

### Transcriptome-wide characterization of *in situ* aged and *ex vivo* cultured dermal fibroblasts

**Figure 2:**
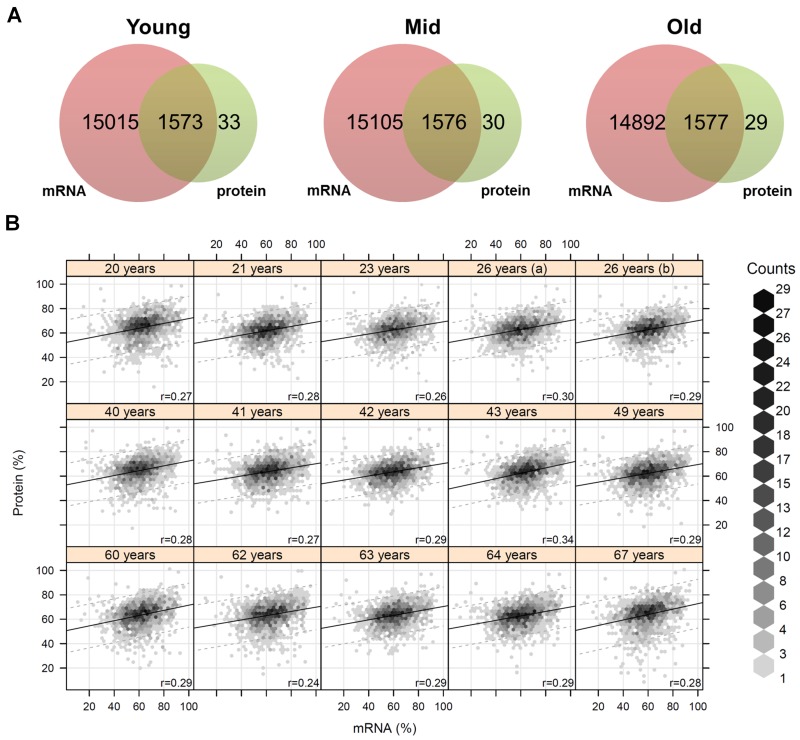
Coverage and correlation of mRNA and protein data (**A**) For each age group the quantified transcripts and proteins were compared. Almost all quantified proteins were also present at the mRNA level. (**B**) Correlation analysis of mRNA and protein abundances for each individual. The mRNA/protein pairs were drawn as hexagons whereby accumulations of pairs are indicated in different colors. Correlation analysis revealed a correlation of about 28% on average between proteins and mRNAs.

Next, we considered gene expression data from the same *in situ* aged fibroblasts already published by Kalfalah and colleagues [10]. It was reported that about 17,000 transcripts were successfully quantified, whereof a total of 137 genes exhibited a significant age-associated differential expression (Tab. S7). Comparison between the proteome and transcriptome data revealed that for almost all proteins (98%) gene expression data were available, but on the contrary the coverage for the 137 age-associated gene by proteome data was quite low (Fig. 2A). Only eight of the 137 differentially changed genes were successfully quantified at the proteomic level and exhibited no agreement with the respective mRNA data concerning age-associated differential expression. Due to intraspecies comparison with an 87% and 98% correlation within the proteome and transcriptome data, respectively, we can exclude technical problems for the minor overlap of the expression data (Fig. S3). The interspecies comparison with about 30% correlation between proteins and mRNAs data was in the expected range (Fig. 2B; Fig. S4). Further detailed analysis revealed that over 63% (10665 quantified mRNAs) of the analyzed genes exhibited constant expression across the different age groups (Fig. S5). In contrast, for all of the 43 age-associated proteins we obtained gene expression data and found out that 77% (33 proteins) of the corresponding transcripts were not changed during aging. Comparison of these age-associated proteins with transcriptome data revealed that age-associated proteins were not differentially regulated at the transcript level.

### MicroRNA profiling of *in situ* aged and *ex vivo* cultured dermal fibroblasts

**Figure 3:**
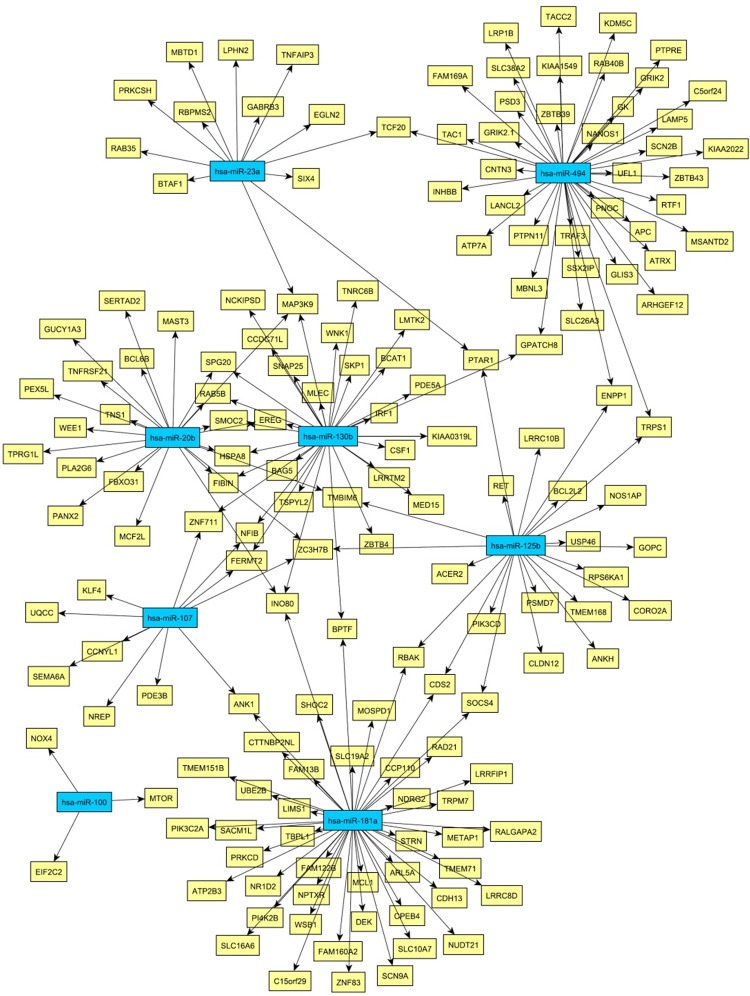
Network of miRNA-mRNA relationships obtained by the combination of target predictions and a penalized regression analysis integrating miRNA and mRNA expression measurements Only miRNAs were considered whose expression levels were associated with age. The miRNA nodes are colored blue and the identified target genes yellow.

**Figure 4:**
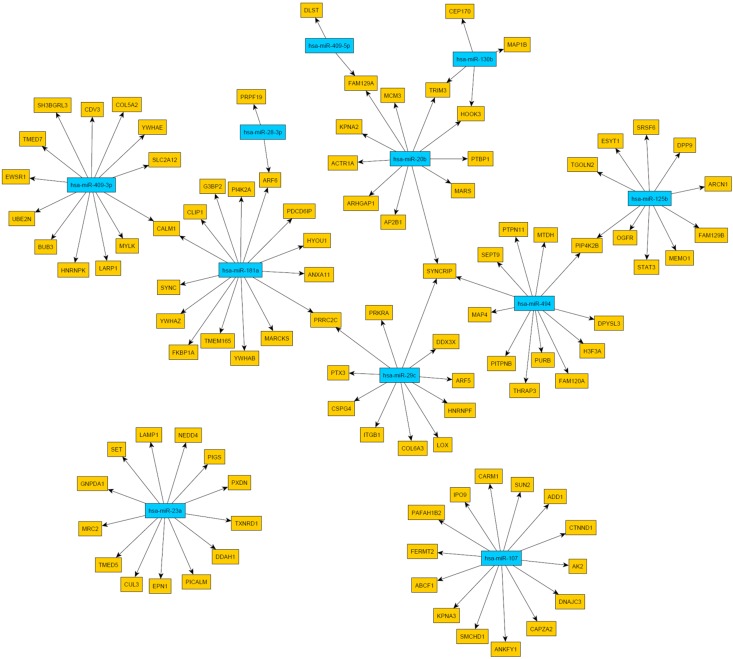
Network of miRNA-protein relationships obtained by the combination of target predictions and a penalized regression analysis integrating miRNA and protein expression measurements Only miRNAs were considered whose expression levels were associated with age. The miRNA nodes are colored blue and the identified target genes yellow.

Then, we were interested whether age-associated changes in miRNA expression might be related to the proteome data and transcriptome data listed above. Microarray-based miRNA profiling revealed 12 miRNAs showing evidence of age-related differential expression (Tab. 2) [11]. To determine if these miRNAs play a role in mRNA abundance, we compared their targets with the mRNAs from Kalfalah and colleagues [10]. This comparison revealed 164 mRNAs as candidate targets of the 12 candidate miRNAs (Fig. 3; Tab. S8). However, none of the targeted mRNAs were found to be significantly altered with age. Moreover 59% (96 mRNAs) exhibited a constant expression during aging. At the protein level we detected three proteins with age-associated alteration that possibly may be explained by miRNA mediated regulation (Tab. 2; Fig. 4). DNAJC3, DDX3X and CALM1 exhibited an anti-correlation with miR-107, miR-29c as well as with miR181a and miR-409-3p, respectively.

**Table 2:** Highest ranked miRNAs whose expression profiles correlate most with age [11] Positive p-values indicate correlation and negative p-values anti-correlation with age. Matched mRNAs were obtained from the published data set of Kalfalah *et al*. [10]. For matching of the miRNAs with proteins, the identified proteins of label-free proteome analysis were used. Matched proteins which were also identified as altered during ageing were indicated in bold.

miRNA	correlation	p-value	Matched mRNA	Matched Protein (alteration on protein level)
**miR-100**	0.458 (up)	0.086	EIF2C2, MTOR, NOX4	
**miR-107**	0.578 (up)	0.024	ANK1, C5ORF13, CCNYL1, FERMT2, KLF4, NFIB, PDE3B, SEMA6A, UQCC, ZC3H7B, ZNF711	ABCF1, ADD1, AK2, ANKFY1, CAPZA2, CARM1, CTNND1, **DNAJC3 (down)**, FERMT2, IPO9, KPNA3, PAFAH1B2, SMCHD1, SUN2
**miR-125b**	−0.503 (down)	0.056	ANKH, BCL2L2, CDS2, CLDN12, CORO2A, ENPP1, GOPC, LOC645978, LRRC10B, NOS1AP, PIK3CD, PSMD7, PTAR1, RBAK, RET, RPS6KA1, SOCS4, TMBIM6, TMEM168, TRPS1, USP46, ZC3H7B	ARCN1, DPP9, ESYT1, FAM129B, MEMO1, OGFR, PIP4K2B, SRSF6, STAT3, TGOLN2
**miR-130b**	0.503 (up)	0.056	BAG5, BCAT1, BPTF, CSF1, EREG, FERMT2, FIBIN, FLJ36031, GPATCH8, HSPA8, INO80, IRF1, KIAA0319L, LMTK2, LRRTM2, MAP3K9, MED15, MLEC, NCKIPSD, NFIB, PDE5A, RAB5B, SKP1, SMOC2, SNAP25, SPG20, TNRC6B, TSPYL2, WNK1, ZBTB4, ZNF711	CEP170, HOOK3, MAP1B, TRIM3
**miR-181a**	−0.58 (down)	0.023	ANK1, ARL5A, ATP2B3, BPTF, C15ORF29, CCP110, CDH13, CDS2, CPEB4, CTTNBP2NL, DEK, FAM122B, FAM13B, FAM160A2, INO80, LIMS1, LRRC8D, LRRFIP1, MCL1, METAP1, MOSPD1, NDRG2, NPTXR, NR1D2, NUDT21, PI4K2B, PIK3C2A, PRKCD, RAD21, RALGAPA2, RBAK, SACM1L, SCN9A, SHOC2, SLC10A7, SLC16A6, SLC19A2, SOCS4, STRN, TBPL1, TMEM151B, TMEM71, TRPM7, UBE2B, WSB1, ZNF83	ANXA11, ARF6, **CALM1 (up)**, CLIP1, FKBP1A, G3BP2, HYOU1, MARCKS, PDCD6IP, PI4K2A, PRRC2C, SYNC, TMEM165, YWHAB, YWHAZ
**miR-20b**	0.441 (up)	0.100	BCL6B, EREG, FBXO31, FIBIN, GUCY1A3, HSPA8, INO80, MAP3K9, MAST3, MCF2L, PANX2, PEX5L, PLA2G6, RAB5B, SERTAD2, SMOC2, SPG20, TMBIM6, TNFRSF21, TNS1, TPRG1L, WEE1, ZC3H7B	ACTR1A, AP2B1, ARHGAP1, FAM129A, HOOK3, KPNA2, MARS, MCM3, PTBP1, SYNCRIP, TRIM3
**miR-23a**	0.499 (up)	0.058	BTAF1, EGLN2, GABRB3, GABRB3, MAP3K9, MBTD1, PRKCSH, PTAR1, RAB35, RBPMS2, SIX4, TCF20, TNFAIP3	CUL3, DDAH1, EPN1, GNPDA1, LAMP1, MRC2, NEDD4, PICALM, PIGS, PXDN, SET, TMED5, TXNRD1
**miR-494**	−0.564 (down)	0.029	APC, ARHGEF12, ATP7A, ATRX, C11ORF61, C20ORF103, C5ORF24, CNTN3, ENPP1, FAM169A, GK3P, GLIS3, GPATCH8, GRIK2, INHBB, KDM5C, KIAA0776, KIAA1549, KIAA2022, LANCL2, LOC344593, LRP1B, MBNL3, NANOS1, PNOC, PSD3, PTPRE, RAB40B, RTF1, SCN2B, SLC26A3, SLC38A2, SSX2IP, TAC1, TACC2, TCF20, TRAF3, TRPS1, ZBTB39, ZBTB43	SEPT9, DPYSL3, FAM120A, H3F3A, MAP4, MTDH, PIP4K2B, PITPNB, PTPN11, PURB, SYNCRIP, THRAP3
**miR-29c**	0.488 (up)	0.065		ARF5, COL6A3, CSPG4, **DDX3X (down)**, HNRNPF, ITGB1, LOX, PRKRA, PRRC2C, PTX3, SYNCRIP ARF6, PRPF19
**miR-28-3p**	0.454 (up)	0.089		ARF6, PRPF19
**miR-409-3p**	−0.461 (down)	0.083		BUB3, **CALM1 (up)**, CDV3, COL5A2, EWSR1, HNRNPK, LARP1, MYLK, SH3BGRL3, SLC2A12, TMED7, UBE2N, YWHAE
**miR-409-5p**	−0.522 (down)	0.046		DLST, FAM129A

### Age-associated biological processes

**Figure 5:**
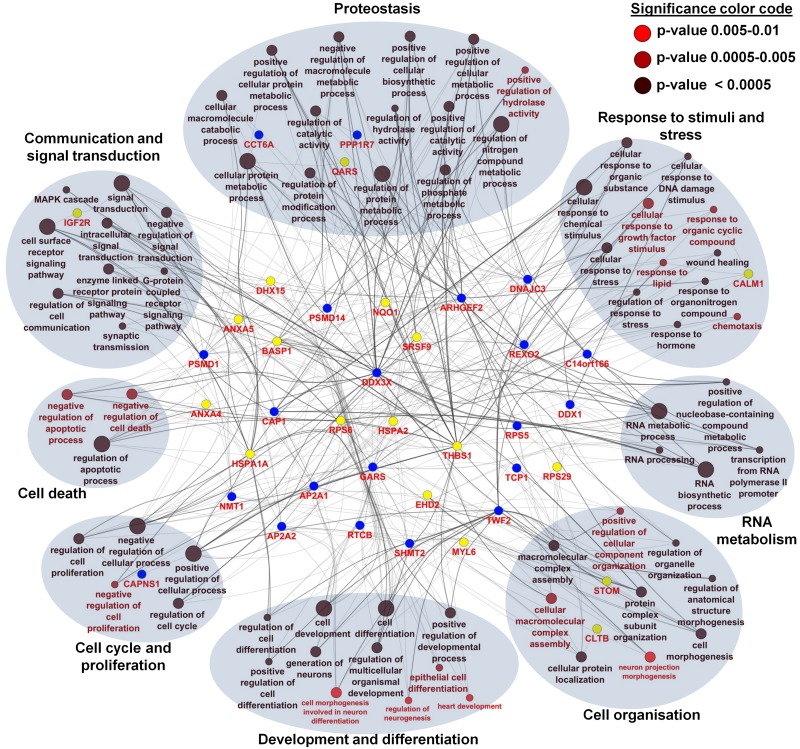
Network and enrichment analysis of age-associated altered proteins using Gene Ontology categories for biological processes For the 43 age-associated altered proteins, a total of 71 unique biological processes were identified as highly enriched (p ≤ 0.01). Different p-values are color coded as follows: p < 0.0005 (black), p = 0.0005-0.005 (deep red), p = 0.005-0.01 (bright red). Proteins were color coded according to their regulation profile: increasing (yellow) and decreasing (blue). Terms describing a similar biological process were grouped according to GO slim categories. A total of eight main categories of GO terms were identified: proteostasis (8 proteins), response to stimuli and stress (9 proteins), development and differentiation (7 proteins), cell organization (15 proteins), cell communication and signal transduction (13 proteins), RNA metabolism (17 proteins), cell cycle and proliferation (25 proteins) and cell death (13 proteins).

With the integration of data from proteome, transcriptome and miRNA analysis we assessed a broad spectrum of candidate genes/proteins involved in aging processes. Next, we were interested to reveal the biological processes which allow us to predict aging processes at the functional level. Kalfalah and colleagues reported on 117 age-related biological processes based on the transcriptome data (Tab. S9) [10]. From the proteome data, we deduced for the 43 age-associated proteins 71 unique biological processes as being significantly enriched (p ≤ 0.01; Tab. S10). We found eight main categories of GO terms: ‘proteostasis’, ‘response to stimuli and stress’, ‘development and differentiation’, ‘cell organization’, ‘cell communica-tion and signal transduction’, ‘RNA metabolism’, ‘cell cycle and proliferation’ and ‘cell death’ (Fig. 5). Remarkably, comparison of biological processes related to the identified age-associated mRNA and protein candidates revealed ‘cell cycle’, ‘development’, ‘transcription’, ‘translation’, ‘regulation of actin cytoskeleton’, ‘proteasome’, ‘RNA and protein metabolism’, ‘signaling’, ‘RNA biosynthesis’, ‘oxidative phosphorylation’, ‘respiratory electron transport’ and ‘signal transduction’ as highly enriched in both transcriptome and proteome data. Interestingly, we revealed most of these processes by the enrichment analysis of 164 target mRNAs of the 12 identified age-associated candidate miRNAs (Tab. S11). Although not confirmed at the protein level or by mRNA alteration, enrichment analysis showed that identical age-associated biological processes such as ‘signal transduction’, ‘cell communication’, ‘cell death’, ‘RNA and protein metabolism’, ‘cell communication’, ‘response to stimuli’, ‘development’, ‘proliferation’, ‘cellular component organization’ and ‘RNA and protein biosynthesis’ were highly enriched. The comparisons between different data sets by the deduced biological processes resulted in a significant overlap and led to a biological processes-oriented picture of aging in fibroblasts not expected from the single gene/protein level.

## DISCUSSION

Deciphering the molecular and cellular mechanisms of aging *in vivo* is far from complete. Here, we analyzed an *ex vivo* model of *in situ* aged human dermal fibroblasts by means of an unbiased proteome-wide approach and integrated the data with those obtained by mRNA and miRNA expression profiling. Previous studies proposed that the aging process of an organ is mainly determined by stromal changes, as exemplified by the role of dermal fibroblasts and the dermal extracellular matrix in skin aging [2]. As the stroma is a highly stationary compartment, homeostasis is primarily based on cellular adaptation and damage clearance and thereby prone to age-related changes. As the major cell source in the stroma and a long-lived cell system, fibroblasts are able to accumulate aging-associated alterations and consequently adapt their cellular functions. An *ex vivo* model of *in situ* aged fibroblasts has been suggested to closer represent the *in vivo* situation of aging than the commonly used senescence-related *in vitro* aging models [12].

Based on the integration of comprehensive data sets from expression studies at the protein, mRNA and miRNA levels, we present here a synoptic view of molecular changes associated with the aging of human dermal fibroblasts *in situ* (in the skin). We demonstrate that 47% and 63% of the proteins and transcripts expressed in these cells exhibited a constant abundance across different donor age groups. A further observation supporting the moderate age-associated cellular phenotype was the low number of significantly changed mRNAs, proteins and miRNAs. In our previous study, a total of 117 mRNAs [10] and 12 miRNAs [11] revealed age-dependent alteration in the same dermal fibroblast cultures investigated here. Our present analyses showed 43 proteins with altered expression in these cells according to the different donor age groups. Remarkably, we found no overlap between the mRNA and protein expression data for these 43 proteins. This could be due to the fact that individual proteins or transcripts may not meet the threshold for statistical significance as the used technologies have different noise levels. On the other hand, it has been shown and confirmed by our data that in mammalian cells approximately only one third of the mRNA abundance is reflected in the proteome [13]. However, the fact that 77% of the age-associated proteins were not linked to expression changes of the corresponding transcripts suggested that most of the age-associated alterations detected at the proteome level are likely caused by other processes, such as post-transcriptional regulation, translation efficiency, protein stability or modifications, rather than by differential regulation of gene expression. The analysis of miRNA expression data suggest that the age-associated abundance changes observed for three candidate proteins (DNAJC3, DDX3X, CALM1) are caused by post-transcriptional regulation through corresponding miRNAs, i.e. miR-107, miR-29c, miR-181a and miR-409-3p, which may target the transcripts of these proteins and showed an inverse expression pattern compared to these proteins in our fibroblast cultures. Furthermore, considering the abundance of the proteins we showed that the age-associated alterations occur mostly in the groups of proteins with middle to low abundance. The observed age-associated decrease of low abundant proteins in fibroblast cultures was linked to electron transport chain, oxidative phosphorylation and cellular respiration. This is in agreement with our previous work showing that a decline in cell proliferation and protein synthesis of *in situ* aged fibroblasts is a consequence of inadequate mito-nuclear signaling [10].

It has been shown that the majority of fibroblasts *in situ* are in a reversible growth arrested state and only a minority is senescent [14, 15]. Moreover, it has been pointed out that cell cycle arrest is a common feature of senescent and quiescent cells, while cellular senescence emerges from cellular quiescence by geroconversion, which is an irreversible process that is driven by p53- and mTOR-signaling and entails the loss of proliferative potential and the acquisition of cellular hallmarks of aging [16]. The data presented here are in good agreement with this concept. Age-associated alterations of the proteome, transcriptome and miRNAome of fibroblasts observed here, indicated an age-associated decline in proliferative capacity and age-associated features of geroconversion manifesting in the acquisition of several hallmarks of fibroblast aging [4] encompassing altered proteostasis, altered cell-cell signaling, hypertrophy and altered cell organization, altered stress response, altered RNA metabolism and translation.

### Age-associated loss of proliferative potential

The limited proliferating potential of *in vitro* cultured cells has been widely investigated in the context of cellular aging [4]. We identified six proteins known to be involved in cell cycle regulation as showing age-associated expression changes in dermal fibroblasts. These included reduced expression of the two subunits TCP1 and CCT6A of the T-complex protein 1 that plays an important role in the transition from S to M phase [17]. Another protein with age-associated decreased expression was CAPNS1 which is the regulatory subunit of the calcium-regulated non-lysosomal thiol-protease. Knock-down of CAPNS1 inhibited proliferation and cell growth [18]. Similarly, DNAJC3, an inhibitor of the kinase PKR, showed reduced expression in aged fibroblasts. Reduced expression of DNAJC3 resulted in increased activity of PKR and phosphorylation of EIF2A, whereby protein translation and cell growth is down-regulated [19]. The protein DDX3X, a multifunctional ATP-dependent RNA helicase involved in several steps of gene expression, also demonstrated lower expression in fibroblasts from aged versus young donors. DDX3X is important for translation of Cyclin E1 mRNA and thereby may stimulate cell cycle progression [20]. In contrast THBS1 protein abundance increased with age in cultured fibroblasts. Previous data indicate that THBS1 may cause cell cycle arrest of endothelial cells by increasing the amounts of p21 and unphosphorylated RB1 [21]. Collectively, our findings on aging-associated expression of cell cycle regulatory proteins are in line with a role of reduced proliferation capacity of aged fibroblast discussed in skin aging and provide candidate proteins for further functional validation of this concept.

### Age-associated features of geroconversion

#### Age-associated alterations of proteostasis

Our analyses revealed a role of alterations in proteostasis in fibroblast aging. The central role of proteostasis has been intensively analyzed in the context of aging, whereas defects of proteostasis may commonly lead to misfolding, aggregation, and accumulation of proteins resulting in cellular damage and tissue dysfunction [1]. We identified five chaperones and co-chaperones with altered expression in *in situ* aged fibroblasts, which fits to the role of chaperones as part of the proteostasis network during aging. Expression of the co-chaperone DNAJC3 was found to be decreased with age. DNAJC3 plays an important role in the unfolded protein response during ER stress [22]. The chaperones TCP1 and CCT6A, which are involved in the folding of cytoskeleton proteins, also showed decreased expression levels in fibroblasts from older donors. In contrast, expression of certain chaperones of the Hsp70 family (HSPA1A/B and HSPA2) increased with age. Both proteins are involved in protein folding and unfolded protein response [23]. The abundance change of chaperones of the Hsp70 family could be seen as a response of the aged fibroblasts to cope with increasing amount of unfolded proteins due to a decline of proteasome activity. We found that two proteasomal proteins, PSMD1 and PSMD14, which are involved in the ATP-dependent degradation of ubiquitinated proteins, demonstrated reduced expression in aged fibroblasts. The decrease of regulatory and catalytically components of the proteasome may result in an accumulation of age-associated toxic aggregates. The proteins related to protein folding and proteasome activity that demonstrated age-associated differential expression in our proteome analyses thus point to a dominant role of impaired proteostasis in *in situ* aged dermal fibroblasts.

#### Age-associated alterations of cell organization and cytoskeletal architecture

Impaired cell organization and cytoskeletal alterations lead to morphological changes such as an increased cell volume and cell surface [4]. We identified CAP1 as showing decreased expression in aged fibroblasts. CAP1 accelerates the depolymerization of F-actin, regenerates polymeriuable G-actin and recycles cofilin, which binds to F-actin and exhibits pH-sensitive F-actin depolymerizing activity [24]. Down-regulation of CAP1 results in accumulation of F-actin, decrease of G-actin, changes in cofilin phosphorylation, larger cell size and altered cell motility [25]. This is in line with the findings of an increase in filamentous F-actin and a decrease of G-actin in *in situ* aged fibroblasts [26]. We also found increased expression of SYNPO2, a protein that stabilizes F-actin by bundling it to fibrils. SYNPO2 up-regulation may lead to an increase of actin filaments and thereby to increased stiffness [26]. A third interesting protein involved in actin dynamics is TWF2, whose expression was decreased during *in situ* aging. TWF2 inhibits actin polymerization by sequestering G-actin [27]. Down-regulation of TWF2 may result in an increased polymerization of G-actin to F-actin filaments and a decline in cell motility. In the context of cellular transport and complex assembly, we identified nine proteins related to endocytosis as showing age-associated expression differences, including reduced expression of two essential subunits of the AP-2 adaptor complex (AP2A1 and AP2A2), which is necessary for the assembly of vesicles. Because fibroblasts have been estimated to internalize more than 200% of their entire surface area each hour [28], a decrease in clathrin-mediated endocytosis may lead to impaired cellular and pericellular homeostasis in aged skin.

#### Cell death in aging

Impaired regulation of apoptosis may lead to an accumulation of damaged and nonfunctional cells in aging [4]. An important aspect of the apoptotic machinery is the endocytic uptake of IGFBP3 which targets nuclear regulators of apoptosis [29]. We identified several proteins related to endocytosis as showing age-associated expression differences (see above). This may cause decreased uptake of IGFBP3, which in turn may contribute to apoptosis resistance of fibroblasts [30]. Other authors found that IGFBP3 accumulates at senescence in the conditioned medium of human fibroblasts and not in the nucleus where it promotes apoptosis [31]. We also identified proteins directly involved in apoptosis. Expression of HSPA1A/B, which protects proteins against aggregation, was found as being increased with age. HSPA1A/B may prevent active CASP3 from cleaving the transcription factor GATA-1 and inducing apoptosis [32]. Higher levels of HSPA1A/B thus may inhibit CASP3-mediated apoptosis and cause resistance of senescent fibroblasts to apoptotic cell death by down-regulating CASP3 [30]. One may speculate that age-associated impaired apoptosis may lead to an accumulation of nonfunctional fibroblasts which are no longer able to maintain a proper stroma. Instead, they release inflammatory cytokines and disturb the extracellular matrix. Thus, our data support the conclusion that cell death via apoptosis is diminished in dermal fibroblasts during *in situ* aging.

#### Stress response in aging

Activated stress response has been suggested to be a part of the aging process [33]. The ER is sensitive to the accumulation of misfolded proteins, and relays stress signals to the ER mitochondria calcium cycle [34]. In addition, alterations in cellular calcium homeostasis correlate with the occurrence of ER stress and increased susceptibility to protein folding stress [35]. There is evidence that ER calcium homeostasis plays an important role in maintaining cells, since depletion of ER calcium causes growth arrest and cell death [36]. We found eight proteins with age-associated altered expression that are functionally related to calcium stimuli and unfolded protein response. DNAJC3 is an important player in unfolded protein response and its decreased expression leads to an increased accumulation of the transcription factors ATF4 and CHOP, followed by suppressed PPARG transcription [22]. Due to the suppression of PPARG, ER stress can enhance pro-inflammatory NF-κB activation, which in turn leads to an increase of IL8 production [37]. An increased level of IL8 is closely associated with cellular senescence [9] and aging in dermal fibroblasts (Waldera-Lupa et al., unpublished data). We also identified several calcium sensitive proteins, which demonstrated age-associated expression changes. Thus, *in situ* aged fibroblasts undergo an alteration in calcium homeostasis and an increase of unfolded/misfolded proteins leading to an activation of ER stress response.

#### Age-associated alterations of cell-cell communication

##### Alterations in EGFR-signaling

Fibroblasts are known to undergo phenotypic changes when they change from their normal, relatively quiescent state to a proliferative and contractile phenotype, in which they are referred to as myofibroblasts. Differentiation to myofibroblasts can be mediated by TGF-β1 and also involves EGFR signaling [38]. In our study, we found reduced expression of ARHGEF2, a downstream effector of EGFR signaling, in fibroblasts from aged donors. ARHGEF2 is an activator of Rho-GTPases and involved in barrier permeability, cell motility and innate immune response [39]. The decrease of ARHGEF2 suggests an impaired EGFR signaling and a loss of cell motility, which in turn leads to a reduction of wound healing capabilities of aged skin. This confirms previous findings showing that the reduction of EGFR resulting decreases differentiation capacity and cell motility of fibroblasts [40]. We found three additional proteins (BASP1, CAP1, TWF2) with a potential role in development and differentiation of fibroblasts, which demonstrated age-associated expression changes. Among these, CAP1 is crucial for the epidermal permeability barrier and is, thereby, indispensable for skin development [41].

##### Alterations of signal molecule secretion

An interactive communication exists between fibroblasts and their environment [4]. We recently investigated the age-associated secretory phenotype of *in situ* aged fibroblasts, which was characterized by altered secretion of a various matrix metalloproteases, cytokines and other proteins (Waldera-Lupa et al., unpublished data). However, little is known about the intracellular pathways leading to the age-associated secretory phenotype of *in situ* aged fibroblasts. Recently, NF-κB signaling was found to be activated in several tissues with aging [42]. One of the proteins involved in NF-κB signaling is ANXA6, whose expression increased with age. Overexpression of ANXA6 results in the activation of NF-κB [43]. We also found two target proteins of NF-κB, HSPA1A/B and THBS1, as being up-regulated in fibroblasts from aged donors. This might be a direct response to stress mediated by inflammation factors, such as cytokines and is in agreement with our secretome study, where we found increased secretion of several cytokines with age. In addition, our study suggests that mTOR signaling, a key modulator of geroconversion, aging and age-related disease [44, 45], is involved in fibroblast aging. We identified increased age-associated expression of SRSF9, a promoter of mTOR activation. Thus, our data support a role of mTOR and NF-κB signaling in fibroblast aging and the development of a fibroblast-specific age-associated secretory phenotype.

#### RNA metabolism and translation in aging

Impaired function of the RNA machinery plays an important role in aging [46]. Our proteome study revealed decreased expression of a modulator of RNA-polymerase II activity, CGI-99, in aged fibroblasts. Further, we found evidence that RNA-processing may be altered during fibroblast aging. In fact other authors reported that expression patterns of alternatively spliced mRNA change during aging [4]. We identified age-associated expression changes of four proteins (SRSF9, DDX1, DDX3X, DHX15) involved in RNA-splicing, with three of the proteins are functioning as RNA-helicases and therefore are important for translation. For example, an age-associated decrease of DDX3X expression may result in decreased protein synthesis and impaired protein homeostasis (see above). A further indication of impaired translation during aging is provided by altered expression levels of ribosomal proteins, of which we identified three (RPS5, RPS6, RPS29) in our study. The observed up-regulation of HASPA1A/B, a regulator of AUF1, is of interest since AUF1 is involved cytokine mRNA degradation, which is blocked by binding to HASPA1A [47]. Thus, HASPA1A/B up-regulation may increase cytokine mRNA abundance and hence cytokine production. This is in agreement with our previous data on the aging-associated secretome of dermal fibroblasts (Waldera-Lupa et al., unpublished data). A further aspect of RNA-degradation is the elimination of harmful RNAs generated at DNA double-strand breaks (DSB). The helicase DDX1 plays an RNA clearance role at DSB sites, thereby facilitating the template-guided repair of transcriptionally active regions of the genome [48]. Moreover, we identified the oligoribonuclease REXO2 as being down-regulated during aging. REXO2 is involved in the degradation of small single-stranded RNA and DNA oligomers and in the recycling of nucleotides [49]. Decrease of DDX1 and REXO2 thus might lead to the synthesis and accumulation of defect or misfolded proteins in aged fibroblasts.

## METHODS

### Cell isolation and culture

Cell isolation and culture were performed as previously described [10]

### Sample preparation for MS-analysis

Human dermal fibroblasts were cultivated under standardized conditions until passage three as described above. Cells were harvested, homogenized and lysed in urea-buffer (2 M thiourea, 7 M urea, 30 mM Tris-HCL, pH 8.0). Subsequently, total protein amount was determined using BCA protein assay (Thermo Scientific Pierce, Rockford, USA) and 10 μg of each sample were proteolytic digested with trypsin (Promega, Mannheim, Germany). Here, the proteins were incubated with 10 mM dithiothreitol at 56 °C for 45 min in Amicon Ultra filters (3 kDa cut-off; Millipore, Billerica, USA). Subsequently, 0.55 M iodoacetamide was added to the mixture and incubated for 30 min at room temperature and light protected. The mixture was centrifuged for 30 min at 14,000 x g and 4 °C. Next, 50 mM ammonium hydrogen carbonate was added and again centrifuged for 60 min at 14,000 x g and 4 °C. Proteins were recovered and 1:50 trypsin was added. Digestion was performed at 37 °C for 12 h. Afterwards, 0.1% trifluoroactic acid (TFA) was added to stop the digestion.

### Mass spectrometric analysis

Mass spectrometric analyses of proteomes were carried out using highly reproducible and stable LC-MS/MS system and a label-free approach for quantification. Peptides of each sample were analyzed with a nano-HPLC/ESI-MS system composed of an RSLCnano HPLC and a LTQ Orbitrap Velos (Thermo Fisher Scientific, Bremen, Germany) mass spectrometer equipped with a nano-electrospray ion source. Each sample was loaded onto a trap C_18_ trapping column (2 cm × 100 *μ*m × 5*μ*m, 100 Å, Thermo Fisher Scientific) and desalted with 0.1% TFA for 10 minutes. Peptides were eluted from the trap, separated with an analytical column (Acclaim PepMap RSLC C_18_; 25 cm × 75 *μ*m × 2 *μ*m, 100 Å, Thermo Fisher Scientific) for 120 minutes with a flow rate of 300 nL/min, and sprayed into the MS. The Orbitrap parameters were as follows: spray voltage, 1.4 kV; ion transfer tube temperature, 275 °C; collision gas, helium; collision gas pressure, 1.3 mTorr; normalized collision energy for MS/MS, 35%. MS-spectra were acquired in the Orbitrap with a mass range of 300-1500 m/z and a resolution of 60,000. The Orbitrap was operated in a TOP4 data-dependent mode to automatically switch between MS and MS/MS acquisition. MS/MS-spectra were acquired applying gas-phase fractionation. Therefore, five *m/z* areas were used for precursor selection: 300-450 *m/z*, 450-550 *m/z*, 550-650 *m/z*, 650-800 *m/z* and 800-1500 *m/z* (for more information see Fig. S6). Polysiloxane (445.120030 Th) was used as lock mass. For MS/MS, ions were isolated with an isolation width of 2 *m/z* and fragmented using collision-induced dissociation. MS/MS-spectra were acquired in the linear ion trap in centroid mode. Target ions selected for MS/MS were dynamically excluded for 45 sec. The ion selection thresholds were 500 counts for MS/MS. An activation Q of 0.25 and activation time of 10 ms was applied in MS/MS acquisitions.

### Protein identification and quantification

For protein identification, the Proteome Discoverer (version 1.3, Thermo Fisher Scientific) and MASCOT search engine (version 2.4.1, Matrix Science, London, UK) were used. MS/MS-spectra were searched against the UniProtKB database (date 04/03/2013). Search parameters were as follows: enzyme, trypsin; missed cleavage sites, 2; taxonomy, homo sapiens; precursor mass tolerance, 10 ppm; fragment mass tolerance, 0.4 Da; oxidation of methionine as dynamic modification; carbamido-methylation of cysteine as fixed modification. The false discovery rate was set to 1% (p ≤ 0.01). Label-free quantification of proteins was carried out using Progenesis LC-MS (Nonlinear Dynamics, Newcastle upon Tyne, UK) as describes previously [50]. A minimum of 2 unique peptides were required for quantification. Only proteins identified in 14 of 15 samples were regarded for quantification. Statistical analysis for the proteomes was performed using two approaches: multiple t-tests (ANOVA) and Pearson correlation. For ANOVA analysis a significance threshold of 5% (p ≤ 0.05) was applied. Pearson correlation was performed without any grouping with a significance threshold of 5% (p ≤ 0.05). Stability of protein and mRNA abundance was determined as described elsewhere [51]. Therefore, protein/mRNA intensities were logarithmized (log10) and for each protein/mRNA the absolute mean difference between the age-groups (‘mean young’ – ‘mean middle’; ‘mean young’ – ‘mean old’; ‘mean middle’ – ‘mean old’) was calculated. Afterwards, the ‘two one-sided test’ for equivalence (TOST) was applied to test for similarity of the groups (e.g. H_1_: |‘mean young’ – ‘mean middle’| < equivalence range (ɛ)) [52] [51] [50] [50] [48]. As equivalence range, the three-fold standard deviation (ɛ = 0.3) was used. Proteins/mRNAs whose p-value was significant (p ≤ 0.05) in all of the three pairwise comparisons were marked as unchanged.

### Functional annotation, network and enrichment analysis of proteins

Gene set analysis of unchanged proteins was carried out using Consensus Path DB [53]. The entire identified proteome was used as background list. Enrichment was applied on Gene Ontology, Reactome, Wikipathways and Pathway Interaction Database biological processes. We discarded gene sets that were redundant, had ≤ 5 members or a p-value above 0.01. Enrichment analyses of abundance classes and mitochondria related proteins were carried out using DAVID [54] and the Gene Ontology Biological Process categorization. We discarded processes that had a p-value above 0.05. Network and enrichment analysis was carried out using Cytoscape environment [55] and ClueGo plug-in [56]. Parameters for network analyses were applied as follows: Ontology source, Gene Ontology biological processes or cellular components (date 12/10/2013); statistical test, enrichment/depletion (two-sided hypergeometric test); p-value correction, Benjamini-Hochberg; p-value restriction, p ≤ 0.01; network specificity, ‘medium’; GO term restriction ‘min level = 4′, ‘max level = 8′, min percentage = 4.0; use GO term fusion; use GO term grouping; GO term connection restriction, kappa score ≥ 0.5. The entire identified proteome was used as reference set.

### mRNA/protein correlation analysis

Correlation analysis between measured mRNA-expression levels and protein abundance was performed using R ( http://www.r-project.org). The mRNA intensities where log2 scaled, the lowest 25% based on the median intensities as well as probes with no corresponding EntrezID where discarded. In the case of multiple probes corresponding to a single Entrez GeneID the mean intensity was used for further analysis. The maximum mRNA intensity was set to 100%. For each identified protein the sum intensities were divided by the underlying number of quantified peptides. The protein data was log2 scaled and the maximum value was set to 100%.

### miRNA network and enrichment analysis

The age-associated miRNAs reported by Röck et al. [11] were used to build up a miRNA-gene regulatory network as follows: We built up an initial network for these miRNAs based on target predictions, which were derived from TargetScan [57]. We then integrated gene expression measurements in order to find relationships between miRNAs and genes, which can be deduced by the data. We fitted a linear model with the gene expression data as response and the expression data of its predicted targeting miRNAs as predictor variables. We then performed a multiple linear regression analysis for each gene with elastic net penalty in order to select for miRNAs that have an influence on the gene expression data. For this purpose, we used the *glmnet* package for the R [58] and introduced a negativity constraint to select only for negative miRNA effects. The penalty parameter was determined by 10-fold cross-validation. We finally obtained two networks by applying this procedure to the mRNA and the protein expression data, respectively.

## SUPPLEMENTARY TABLES AND FIGURES




