# Supplementary material for: Proteome-wide analysis reveals an age-associated cellular phenotype of in situ aged human fibroblasts
Source: Aging (Albany NY). 2014 Nov 2;6(10):856–72. doi: 10.18632/aging.100698 (PMC4247387; doi:10.18632/aging.100698)
Supplement: Supplementary file 1 [file aging-06-856-s001.pdf]

SUPPLEMENTARY TABLES

Please follow the link in Full Text version of this manuscript to see the Supplementary Tables listed below:

Table S1. Significantly altered proteins with age.
Table S2. Enrichment analysis of biological processes of proteins with constant expression during aging.
Table S3. Enrichment analysis of abundance classes of young donor' proteins.
Table S4. Enrichment analysis of abundance classes of middle donor' proteins.
Table S5. Enrichment analysis of abundance classes of old donor' proteins.
Table S6. Enrichment analysis of age-associated decreasing (14) and increasing (9) mitochondrial proteins obtained from cluster analysis ($p \leq 0.1$).
Table S7. Significantly altered genes with age published by Kalfalah et al. [10].
Table S8. Genes contained in the miRNA/mRNA network.
Table S9. Biological processes significantly altered with age published by Kalfalah et al. [10].
Table S10. Enriched biological processes of proteins found with an age-associated alteration.
Table S11. Significantly enriched biological processes of the genes identified in the miRNA/mRNA network

SUPPLEMENTARY FIGURES

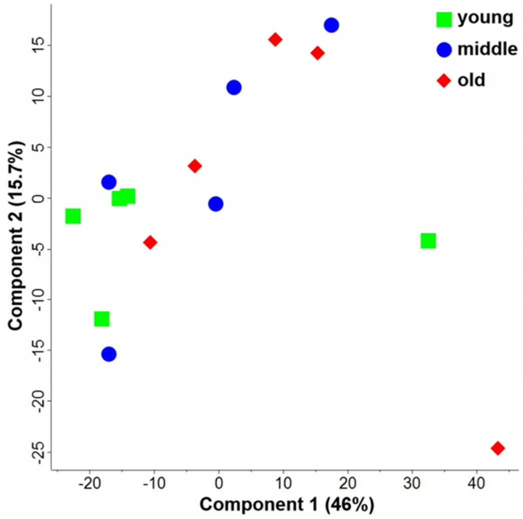

Figure S1. Principle component analysis (PCA) of quantified proteins. The individual age groups are indicated with different colors and signs: young, green squares; middle-aged, blue circles; old, red diamonds. Principal component revealed a heterogeneous protein expression pattern in fibroblasts of the three age groups.

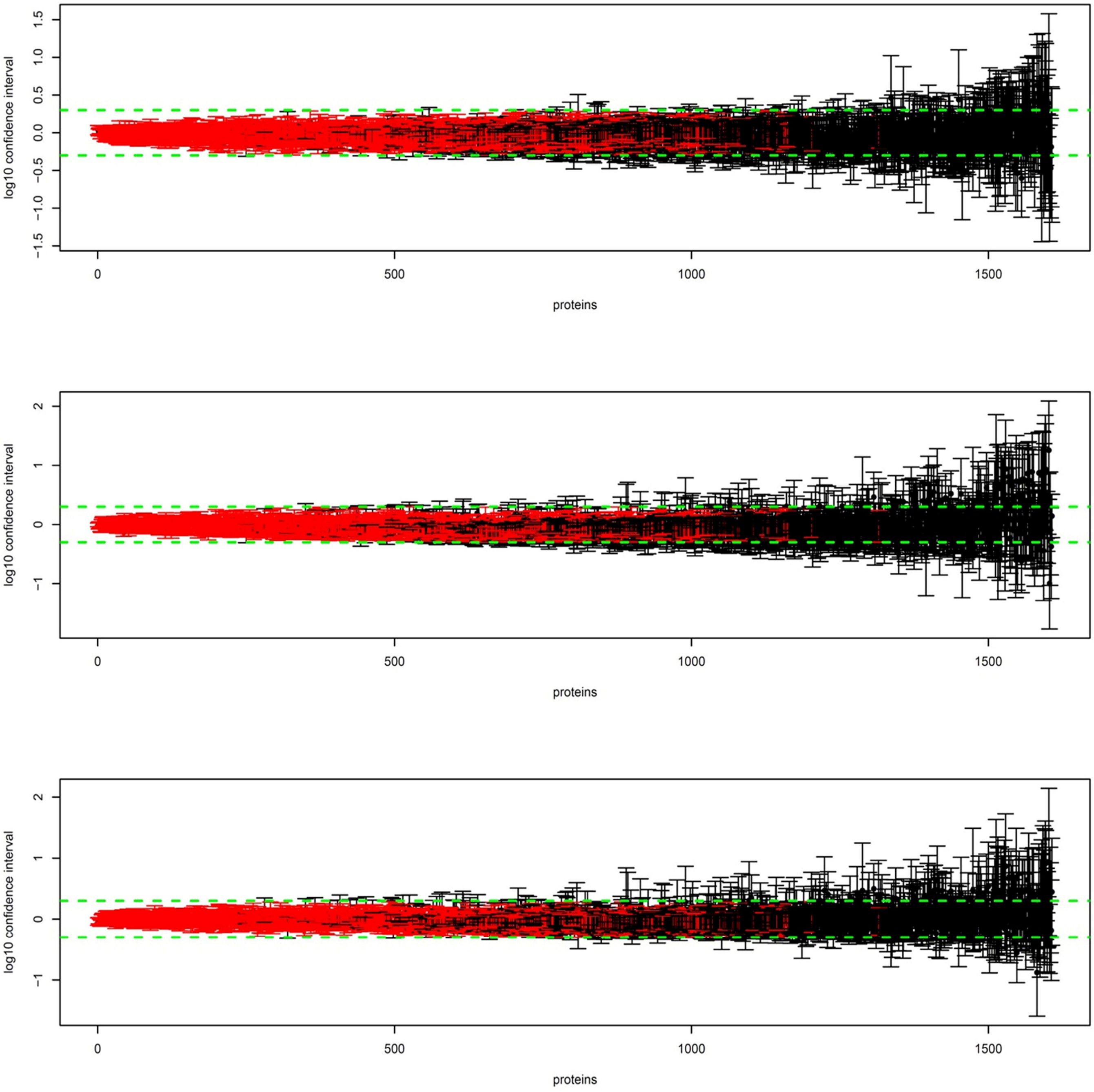

Figure S2. Distributions of confidence intervals of quantifiable proteins. Green line indicates upper and lower limits calculated from the technical variance (three times standard deviation). Significantly unchanged proteins during aging are indicated in red. Remarkably, over 47 % (755 proteins) of quantified proteins exhibited a stable abundance in cultivated fibroblasts across the different donor age groups.

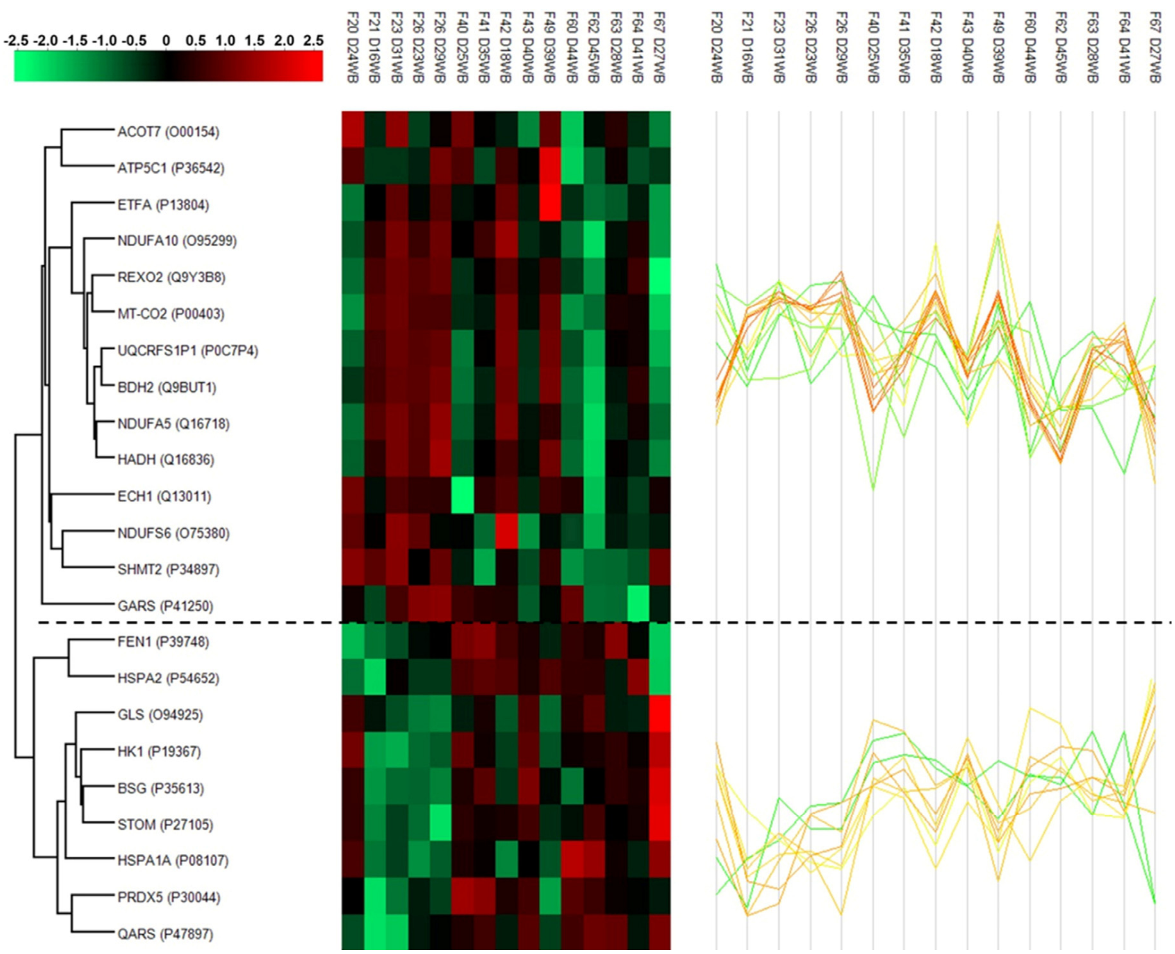

Figure S3. Cluster analysis of expression of mitochondria-related proteins. Cluster analysis of proteins related to mitochondria revealed 14 proteins showing decreasing and 9 proteins showing increasing expression levels with increasing age ($p \le 0.1$).

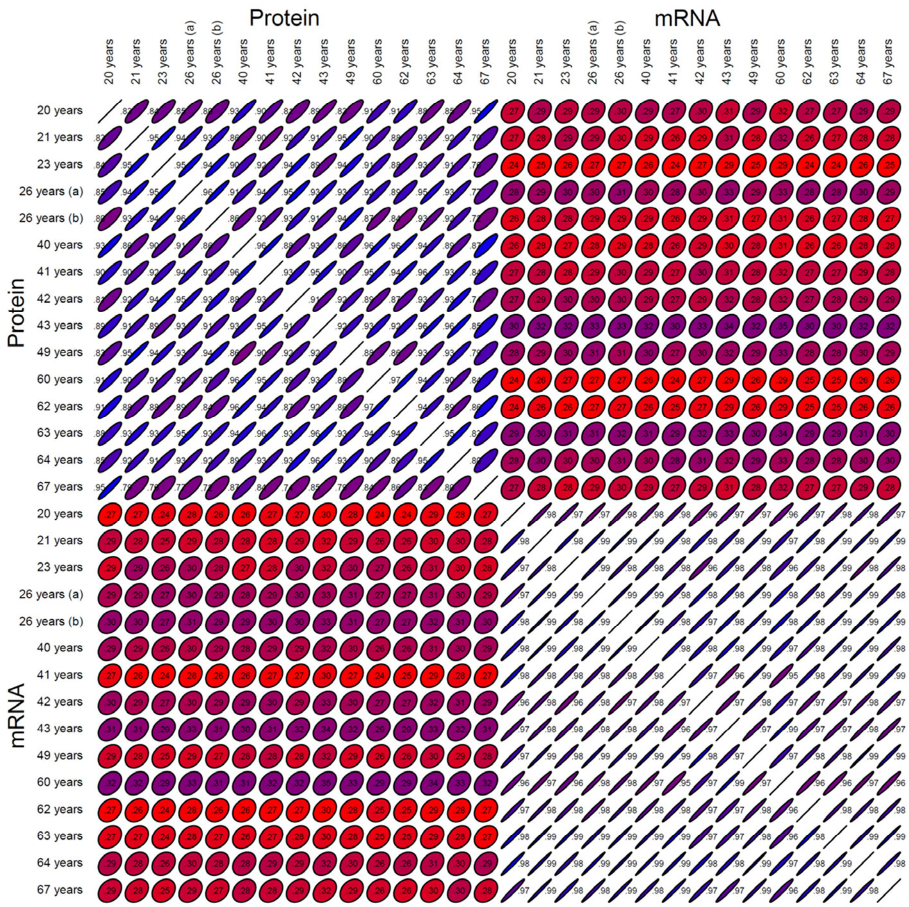

Figure S3. Correlation analysis of mRNA and protein abundances for quantified mRNA/protein pairs. Correlation coefficients are indicated by the ellipse shape. In each column the color code is sorted from highest (blue) to lowest (red) value. Neither inside age group in mRNA or protein data nor in same-sample comparison was an increased correlation observed.

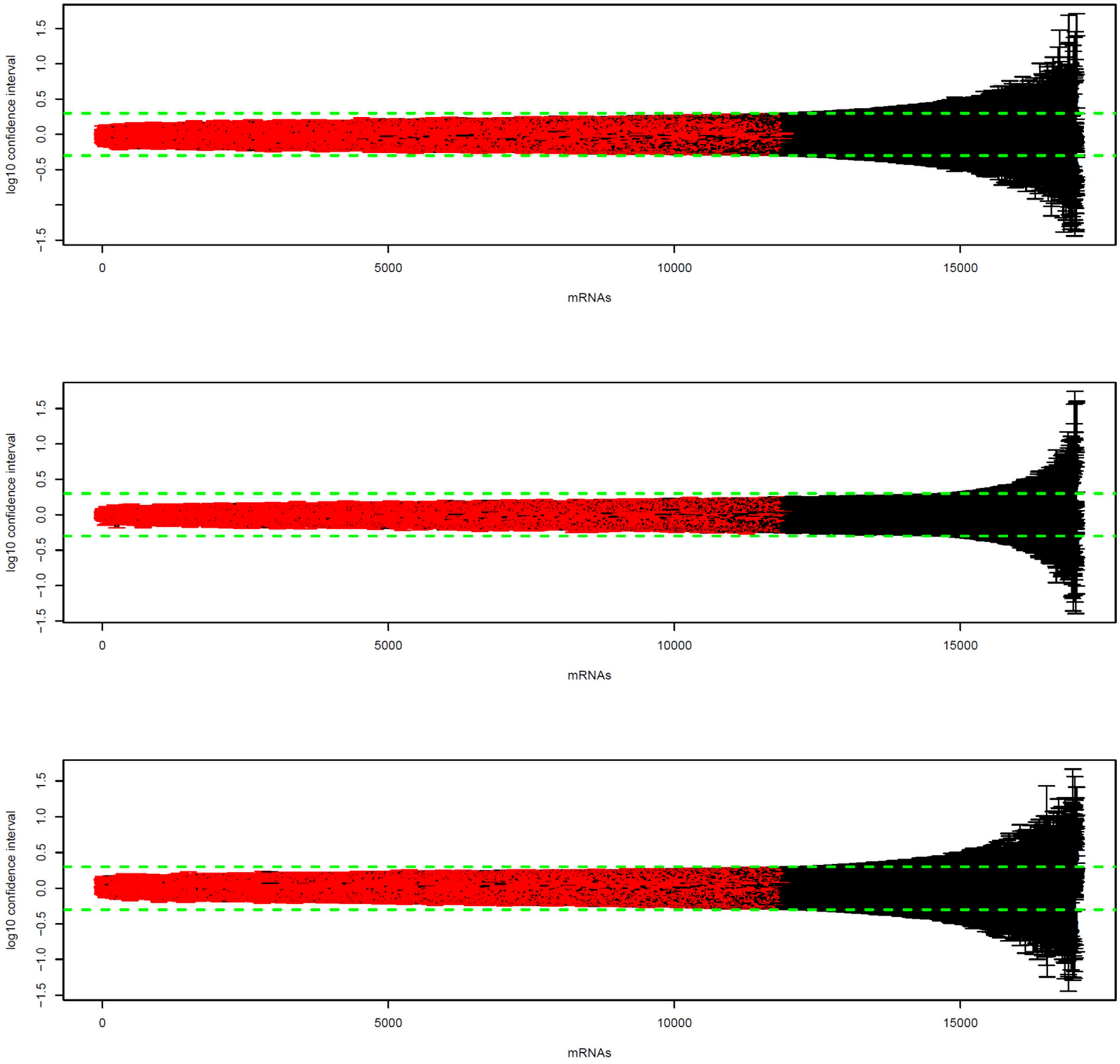

Figure S3. Distributions of confidence intervals of quantifiable mRNAs. Green line indicates upper and lower limits calculated from the technical variance (three times standard deviation). Significantly unchanged mRNAs across the different age groups are indicated in red. Remarkably, over 63 % (10665 mRNAs) of quantified mRNAs exhibited a stable abundance in fibroblasts across the distinct age groups.

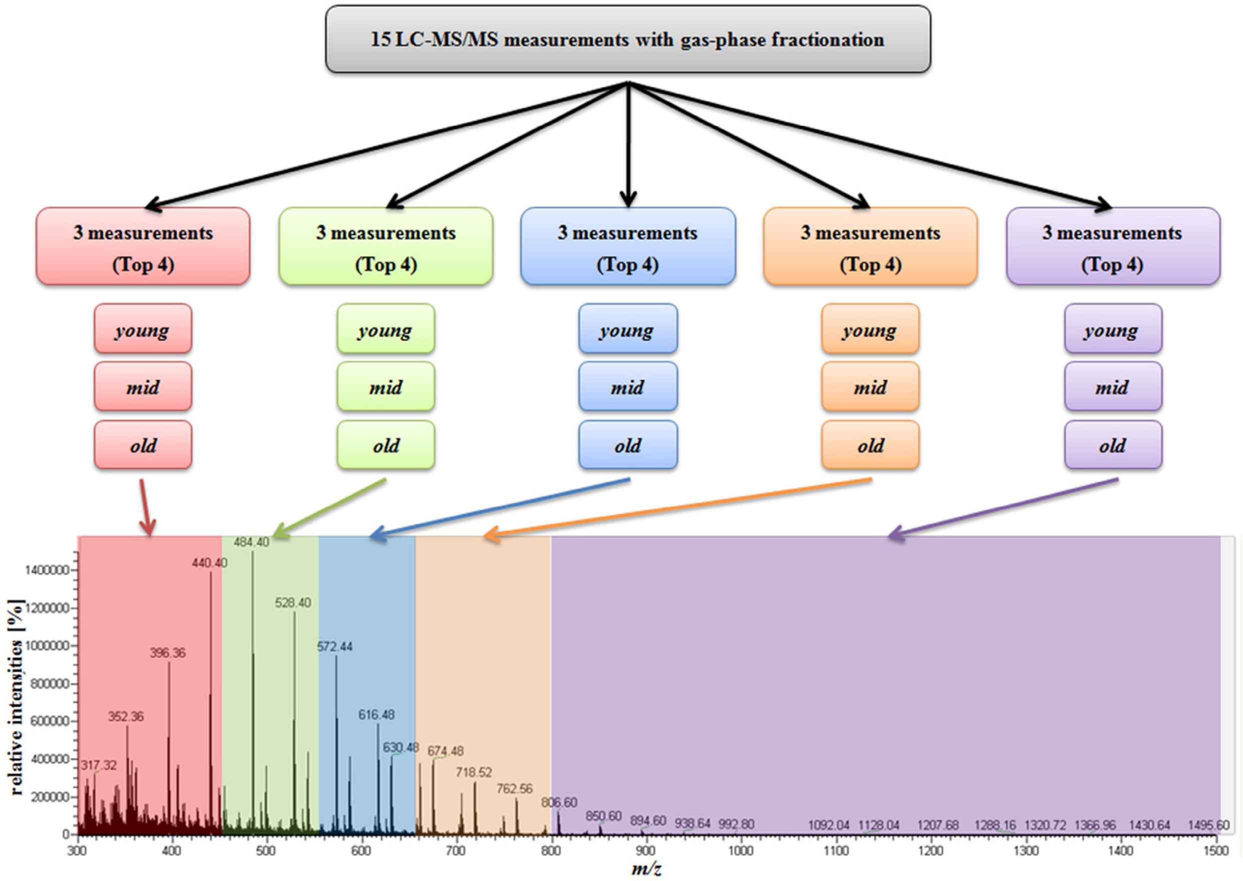

Figure S6. Overview of *m/z* ranges for LC-MS/MS analysis using gas-phase fractionation. The *m/z* segments for precursor selection are color coded as follows: (red: 300-450 *m/z*, green: 450-550 *m/z*, blue: 550-650 *m/z*, orange: 650-800 *m/z*, purple: 800-1500 *m/z*). Each segment was used for three measurements of each age group.
