# Supplementary material for: Proteome-wide analysis reveals an age-associated cellular phenotype of in situ aged human fibroblasts
Source: Aging (Albany NY). 2014 Nov 2;6(10):856–72. doi: 10.18632/aging.100698 (PMC4247387; doi:10.18632/aging.100698)
Supplement: Supplementary file 2 [file aging-06-856-s002.docx]

**SUPPLEMENTARY TABLES**

**Content**

**Tab. S1** Significantly altered proteins with age.

**Tab. S2** Enrichment analysis of biological processes of proteins with constant expression during aging.

**Tab. S3** Enrichment analysis of abundance classes of young donor' proteins.

**Tab. S4** Enrichment analysis of abundance classes of middle donor' proteins.

**Tab. S5** Enrichment analysis of abundance classes of old donor' proteins.

**Tab. S6** Enrichment analysis of age-associated decreasing (14) and increasing (9) mitochondrial proteins obtained from cluster analysis (p ≤ 0.1).

**Tab. S7** Significantly altered genes with age published by Kalfalah et al. [10].

**Tab. S8** Genes contained in the miRNA/mRNA network.

**Tab. S9** Biological processes significantly altered with age published by Kalfalah et al. [10].

**Tab. S10** Enriched biological processes of proteins found with an age-associated alteration.

**Tab. S11** Significantly enriched biological processes of the genes identified in the miRNA/mRNA network

**Table S1 Significantly altered proteins with age.** Quantitative label-free proteome analysis of in situ aged fibroblasts' proteome revealed 43 proteins that are differentially altered during in situ ageing (ANOVA: p ≤ 0.05; Pearson correlation: p ≤ 0.05). For ANOVA analysis donors were grouped according to calendar age into groups 20-30, 40-50 and 60-70 years, with five individual donors in each group.

| Accession | Gene | Description | p-value | Fold change | Regulation | Confidence score | Peptide count | Peptides quantitation | Lowest condition | Highest condition | Constant expression during ageing |
| --- | --- | --- | --- | --- | --- | --- | --- | --- | --- | --- | --- |
| P08133 | ANXA6 | Annexin A6 | 0.001 | 1.5 | up | 1985 | 33 | 30 | Young | Old | Yes |
| P47897 | QARS | Glutaminyl-tRNA synthetase | 0.002 | 1.3 | up | 216 | 6 | 6 | Young | Old | Yes |
| P11717 | IGF2R | Cation-independent mannose-6-phosphate receptor | 0.003 | 2.8 | up | 77 | 2 | 2 | Young | Old | Yes |
| Q6IBS0 | TWF2 | Twinfilin-2 | 0.005 | 1.4 | down | 223 | 4 | 3 | Old | Young | Yes |
| P46782 | RPS5 | 40S ribosomal protein S5 | 0.007 | 2.2 | down | 134 | 2 | 2 | Old | Young | Yes |
| O43143 | DHX15 | Putative pre-mRNA-splicing factor ATP-dependent RNA helicase DHX15 | 0.008 | 1.4 | up | 136 | 3 | 3 | Young | Old | Yes |
| Q99460 | PSMD1 | 26S proteasome non-ATPase regulatory subunit 1 | 0.012 | 1.4 | down | 362 | 7 | 5 | Old | Young | Yes |
| P62273 | RPS29 | 40S ribosomal protein S29 | 0.012 | 1.3 | up | 140 | 2 | 2 | Young | Old | Yes |
| Q9UMS6 | SYNPO2 | Synaptopodin-2 | 0.012 | 1.8 | up | 96 | 2 | 2 | Young | Old | No |
| P40227 | CCT6A | T-complex protein 1 subunit zeta | 0.012 | 1.7 | down | 860 | 12 | 7 | Old | Young | Yes |
| P08758 | ANXA5 | Annexin A5 | 0.012 | 1.4 | up | 1967 | 22 | 22 | Young | Old | Yes |
| Q9Y3I0 | C22orf28 | tRNA-splicing ligase RtcB homolog | 0.013 | 2.8 | down | 251 | 5 | 3 | Old | Young | Yes |
| Q13242 | SRSF9 | Serine/arginine-rich splicing factor 9 | 0.013 | 1.2 | up | 89 | 3 | 2 | Young | Old | Yes |
| P60660 | MYL6 | Myosin light polypeptide 6 | 0.017 | 1.2 | up | 597 | 7 | 7 | Young | Old | Yes |
| Q15435 | PPP1R7 | Protein phosphatase 1 regulatory subunit 7 | 0.022 | 1.6 | down | 290 | 4 | 4 | Old | Mid | Yes |
| Q9Y224 | C14orf166 | UPF0568 protein C14orf166 | 0.022 | 1.9 | down | 398 | 6 | 6 | Old | Young | Yes |
| O00487 | PSMD14 | 26S proteasome non-ATPase regulatory subunit 14 | 0.022 | 1.4 | down | 184 | 4 | 4 | Old | Young | Yes |
| P34897 | SHMT2 | Serine hydroxymethyltransferase, mitochondrial | 0.022 | 1.7 | down | 145 | 3 | 3 | Old | Young | Yes |
| P08107 | HSPA1A | Heat shock 70 kDa protein 1A/1B | 0.022 | 1.7 | up | 1160 | 19 | 9 | Young | Old | Yes |
| Q92499 | DDX1 | ATP-dependent RNA helicase DDX1 | 0.023 | 1.3 | down | 332 | 8 | 7 | Old | Young | Yes |
| P62158 | CALM1 | Calmodulin | 0.024 | 2.7 | up | 238 | 3 | 3 | Young | Old | Yes |
| O94973 | AP2A2 | AP-2 complex subunit alpha-2 | 0.027 | 1.6 | down | 347 | 6 | 3 | Old | Young | Yes |
| Q92974 | ARHGEF2 | Rho guanine nucleotide exchange factor 2 | 0.028 | 2.6 | down | 65 | 2 | 2 | Old | Mid | Yes |
| P09525 | ANXA4 | Annexin A4 | 0.030 | 1.3 | up | 369 | 5 | 5 | Young | Mid | Yes |
| P27105 | STOM | Erythrocyte band 7 integral membrane protein | 0.031 | 2.0 | up | 237 | 4 | 3 | Young | Old | No |
| O00571 | DDX3X | ATP-dependent RNA helicase DDX3X | 0.034 | 1.3 | down | 576 | 10 | 10 | Old | Young | No |
| P04632 | CAPNS1 | Calpain small subunit 1 | 0.034 | 1.6 | down | 287 | 4 | 3 | Old | Mid | Yes |
| P15559 | NQO1 | NAD(P)H dehydrogenase [quinone] 1 | 0.035 | 3.2 | up | 122 | 3 | 3 | Young | Old | No |
| Q96FQ6 | S100A16 | Protein S100-A16 | 0.036 | 1.4 | down | 99 | 2 | 2 | Old | Young | No |
| P09497 | CLTB | Clathrin light chain B | 0.037 | 1.5 | up | 199 | 6 | 3 | Young | Old | No |
| P80723 | BASP1 | Brain acid soluble protein 1 | 0.037 | 1.6 | up | 1070 | 13 | 13 | Young | Old | Yes |
| Q13217 | DNAJC3 | DnaJ homolog subfamily C member 3 | 0.037 | 2.6 | down | 137 | 2 | 2 | Old | Young | Yes |
| O95782 | AP2A1 | AP-2 complex subunit alpha-1 | 0.039 | 1.4 | down | 541 | 10 | 6 | Old | Young | Yes |
| P62753 | RPS6 | 40S ribosomal protein S6 | 0.039 | 1.3 | up | 122 | 3 | 3 | Young | Old | Yes |
| P41250 | GARS | Glycyl-tRNA synthetase | 0.039 | 1.2 | down | 1077 | 17 | 15 | Old | Young | No |
| Q9NZN4 | EHD2 | EH domain-containing protein 2 | 0.039 | 1.4 | up | 922 | 19 | 17 | Young | Mid | Yes |
| Q9Y3B8 | REXO2 | Oligoribonuclease, mitochondrial | 0.040 | 2.5 | down | 158 | 2 | 2 | Old | Young | Yes |
| P07996 | THBS1 | Thrombospondin-1 | 0.044 | 1.5 | up | 780 | 15 | 14 | Young | Old | No |
| P30419 | NMT1 | Glycylpeptide N-tetradecanoyltransferase 1 | 0.045 | 1.4 | down | 88 | 2 | 2 | Old | Young | Yes |
| Q01518 | CAP1 | Adenylyl cyclase-associated protein 1 | 0.048 | 1.1 | down | 1234 | 18 | 17 | Old | Young | Yes |
| P54652 | HSPA2 | Heat shock-related 70 kDa protein 2 | 0.048 | 1.5 | up | 866 | 13 | 2 | Young | Mid | No |
| Q96QV6 | HIST1H2AA | Histone H2A type 1-A | 0.049 | 3.8 | down | 375 | 5 | 2 | Old | Young | No |
| P17987 | TCP1 | T-complex protein 1 subunit alpha | 0.050 | 1.2 | down | 723 | 13 | 11 | Old | Mid | Yes |

**Table S2 Enrichment analysis of biological processes of proteins with constant expression during aging.** The entire identified proteome was used as background list. Enrichment was applied on Gene Ontology, Reactome, Wikipathways and Pathway Interaction Database biological processes. We discarded gene sets that were redundant, had < 5 members or a p-value above 0.01.

| Biological process | Set size | Candidates | p-value | q-value |
| --- | --- | --- | --- | --- |
| Translation | 160(115) | 82 (71.3%) | 5.66E-10 | 5.47E-08 |
| Cap-dependent Translation Initiation | 124(92) | 68 (73.9%) | 1.63E-09 | 5.47E-08 |
| Eukaryotic Translation Initiation | 124(92) | 68 (73.9%) | 1.63E-09 | 5.47E-08 |
| Eukaryotic Translation Elongation | 100(77) | 59 (76.6%) | 2.04E-09 | 5.47E-08 |
| GTP hydrolysis and joining of the 60S ribosomal subunit | 117(91) | 67 (73.6%) | 2.90E-09 | 5.47E-08 |
| L13a-mediated translational silencing of Ceruloplasmin expression | 115(91) | 67 (73.6%) | 2.90E-09 | 5.47E-08 |
| 3, -UTR-mediated translational regulation | 116(91) | 67 (73.6%) | 2.90E-09 | 5.47E-08 |
| Formation of a pool of free 40S subunits | 105(81) | 61 (75.3%) | 3.56E-09 | 5.50E-08 |
| Nonsense Mediated Decay Independent of the Exon Junction Complex | 99(75) | 57 (76.0%) | 6.80E-09 | 8.89E-08 |
| Metabolism of proteins | 532(194) | 122 (62.9%) | 8.87E-09 | 9.89E-08 |
| Eukaryotic Translation Termination | 93(72) | 55 (76.4%) | 9.31E-09 | 9.89E-08 |
| Regulation of gene expression in beta cells | 105(71) | 54 (76.1%) | 1.69E-08 | 1.69E-07 |
| Nonsense Mediated Decay Enhanced by the Exon Junction Complex | 110(78) | 58 (74.4%) | 2.04E-08 | 1.80E-07 |
| Nonsense-Mediated Decay | 110(78) | 58 (74.4%) | 2.04E-08 | 1.80E-07 |
| Peptide chain elongation | 94(73) | 55 (75.3%) | 2.22E-08 | 1.80E-07 |
| Cytoplasmic Ribosomal Proteins | 88(70) | 53 (75.7%) | 3.06E-08 | 2.36E-07 |
| Viral mRNA Translation | 93(72) | 54 (75.0%) | 3.98E-08 | 2.94E-07 |
| SRP-dependent cotranslational protein targeting to membrane | 118(87) | 62 (71.3%) | 9.65E-08 | 6.84E-07 |
| Insulin Synthesis and Processing | 130(89) | 63 (70.8%) | 1.14E-07 | 7.73E-07 |
| Metabolism of mRNA | 178(99) | 68 (68.7%) | 2.27E-07 | 1.49E-06 |
| Gene Expression | 1064(267) | 152 (56.9%) | 1.55E-06 | 9.73E-06 |
| Translation initiation complex formation | 61(50) | 38 (76.0%) | 2.76E-06 | 1.57E-05 |
| Activation of the mRNA upon binding of the cap-binding complex and eIFs, and subsequent binding to 43S | 63(50) | 38 (76.0%) | 2.76E-06 | 1.57E-05 |
| Metabolism of RNA | 226(111) | 72 (64.9%) | 3.06E-06 | 1.68E-05 |
| Ribosomal scanning and start codon recognition | 61(49) | 37 (75.5%) | 4.95E-06 | 2.55E-05 |
| Processing of Capped Intron-Containing Pre-mRNA | 35(18) | 17 (94.4%) | 7.88E-06 | 3.94E-05 |
| Formation of the ternary complex, and subsequently, the 43S complex | 55(43) | 33 (76.7%) | 9.13E-06 | 4.31E-05 |
| mRNA Processing | 126(54) | 39 (72.2%) | 1.69E-05 | 7.55E-05 |
| mRNA Splicing - Major Pathway | 107(53) | 37 (69.8%) | 9.42E-05 | 0.000391 |
| mRNA Splicing | 107(53) | 37 (69.8%) | 9.42E-05 | 0.000391 |
| Cytosolic tRNA aminoacylation | 24(22) | 18 (81.8%) | 0.000287 | 0.00113 |
| tRNA Aminoacylation | 42(23) | 18 (78.3%) | 0.000783 | 0.00283 |
| proteasome complex | 24(21) | 16 (76.2%) | 0.00258 | 0.00914 |

**Table S3 Enrichment analysis of abundance classes of young donor' proteins.** Enrichment analysis was carried out using DAVID and Gene Ontology biological processes. We discarded processes that had a p-value above 0.05.

| GO ID | Term | Count | % | P-Value | Fisher Exact | Fold Enrichment | Class |
| --- | --- | --- | --- | --- | --- | --- | --- |
| GO:0006414 | translational elongation | 36 | 9.4 | 4.1E-06 | 1.4E-06 | 2.1 | I |
| GO:0006457 | protein folding | 28 | 7.3 | 1.7E-05 | 4.9E-06 | 2.2 | I |
| GO:0007010 | cytoskeleton organization | 40 | 10.5 | 5.0E-05 | 2.1E-05 | 1.8 | I |
| GO:0030029 | actin filament-based process | 29 | 7.6 | 1.4E-04 | 5.0E-05 | 2.0 | I |
| GO:0048870 | cell motility | 29 | 7.6 | 2.6E-04 | 9.8E-05 | 1.9 | I |
| GO:0030036 | actin cytoskeleton organization | 28 | 7.3 | 2.8E-04 | 1.1E-04 | 1.9 | I |
| GO:0006007 | glucose catabolic process | 16 | 4.2 | 4.2E-04 | 1.0E-04 | 2.5 | I |
| GO:0019320 | hexose catabolic process | 16 | 4.2 | 4.2E-04 | 1.0E-04 | 2.5 | I |
| GO:0006412 | translation | 50 | 13.1 | 8.8E-04 | 4.8E-04 | 1.5 | I |
| GO:0043067 | regulation of programmed cell death | 45 | 11.8 | 9.8E-04 | 5.2E-04 | 1.6 | I |
| GO:0046365 | monosaccharide catabolic process | 16 | 4.2 | 1.0E-03 | 2.9E-04 | 2.3 | I |
| GO:0010941 | regulation of cell death | 45 | 11.8 | 1.2E-03 | 6.3E-04 | 1.5 | I |
| GO:0042981 | regulation of apoptosis | 44 | 11.5 | 1.3E-03 | 7.2E-04 | 1.5 | I |
| GO:0006096 | glycolysis | 13 | 3.4 | 1.5E-03 | 3.4E-04 | 2.5 | I |
| GO:0046164 | alcohol catabolic process | 16 | 4.2 | 1.6E-03 | 4.6E-04 | 2.2 | I |
| GO:0051789 | response to protein stimulus | 14 | 3.7 | 1.6E-03 | 4.0E-04 | 2.4 | I |
| GO:0006986 | response to unfolded protein | 12 | 3.1 | 2.2E-03 | 5.1E-04 | 2.6 | I |
| GO:0032535 | regulation of cellular component size | 20 | 5.2 | 2.5E-03 | 9.2E-04 | 2.0 | I |
| GO:0030834 | regulation of actin filament depolymerization | 9 | 2.4 | 2.5E-03 | 3.7E-04 | 3.1 | I |
| GO:0051129 | negative regulation of cellular component organization | 16 | 4.2 | 3.3E-03 | 1.1E-03 | 2.1 | I |
| GO:0044275 | cellular carbohydrate catabolic process | 16 | 4.2 | 3.3E-03 | 1.1E-03 | 2.1 | I |
| GO:0043623 | cellular protein complex assembly | 16 | 4.2 | 3.3E-03 | 1.1E-03 | 2.1 | I |
| GO:0043244 | regulation of protein complex disassembly | 12 | 3.1 | 3.6E-03 | 9.0E-04 | 2.4 | I |
| GO:0016052 | carbohydrate catabolic process | 17 | 4.5 | 4.3E-03 | 1.5E-03 | 2.0 | I |
| GO:0006006 | glucose metabolic process | 19 | 5.0 | 4.9E-03 | 1.9E-03 | 1.9 | I |
| GO:0060548 | negative regulation of cell death | 23 | 6.0 | 5.3E-03 | 2.3E-03 | 1.7 | I |
| GO:0043069 | negative regulation of programmed cell death | 23 | 6.0 | 5.3E-03 | 2.3E-03 | 1.7 | I |
| GO:0070271 | protein complex biogenesis | 32 | 8.4 | 6.1E-03 | 3.2E-03 | 1.6 | I |
| GO:0006461 | protein complex assembly | 32 | 8.4 | 6.1E-03 | 3.2E-03 | 1.6 | I |
| GO:0051258 | protein polymerization | 8 | 2.1 | 7.0E-03 | 1.2E-03 | 3.0 | I |
| GO:0008064 | regulation of actin polymerization or depolymerization | 12 | 3.1 | 8.3E-03 | 2.4E-03 | 2.2 | I |
| GO:0030832 | regulation of actin filament length | 12 | 3.1 | 8.3E-03 | 2.4E-03 | 2.2 | I |
| GO:0043066 | negative regulation of apoptosis | 22 | 5.8 | 9.5E-03 | 4.3E-03 | 1.7 | I |
| GO:0019725 | cellular homeostasis | 23 | 6.0 | 1.0E-02 | 4.9E-03 | 1.7 | I |
| GO:0010033 | response to organic substance | 36 | 9.4 | 1.0E-02 | 5.8E-03 | 1.5 | I |
| GO:0032956 | regulation of actin cytoskeleton organization | 13 | 3.4 | 1.1E-02 | 3.7E-03 | 2.1 | I |
| GO:0032970 | regulation of actin filament-based process | 13 | 3.4 | 1.1E-02 | 3.7E-03 | 2.1 | I |
| GO:0045454 | cell redox homeostasis | 13 | 3.4 | 1.1E-02 | 3.7E-03 | 2.1 | I |
| GO:0010639 | negative regulation of organelle organization | 11 | 2.9 | 1.3E-02 | 3.7E-03 | 2.2 | I |
| GO:0043242 | negative regulation of protein complex disassembly | 9 | 2.4 | 1.3E-02 | 3.1E-03 | 2.5 | I |
| GO:0045664 | regulation of neuron differentiation | 9 | 2.4 | 1.3E-02 | 3.1E-03 | 2.5 | I |
| GO:0007051 | spindle organization | 6 | 1.6 | 1.6E-02 | 2.2E-03 | 3.4 | I |
| GO:0043066 | anti-apoptosis | 16 | 4.2 | 1.9E-02 | 8.1E-03 | 1.8 | I |
| GO:0030835 | negative regulation of actin filament depolymerization | 7 | 1.8 | 1.9E-02 | 3.7E-03 | 2.9 | I |
| GO:0050767 | regulation of neurogenesis | 10 | 2.6 | 1.9E-02 | 5.6E-03 | 2.2 | I |
| GO:0046496 | nicotinamide nucleotide metabolic process | 8 | 2.1 | 2.0E-02 | 4.7E-03 | 2.6 | I |
| GO:0019362 | pyridine nucleotide metabolic process | 8 | 2.1 | 2.0E-02 | 4.7E-03 | 2.6 | I |
| GO:0009820 | alkaloid metabolic process | 8 | 2.1 | 2.0E-02 | 4.7E-03 | 2.6 | I |
| GO:0009266 | response to temperature stimulus | 8 | 2.1 | 2.0E-02 | 4.7E-03 | 2.6 | I |
| GO:0006733 | oxidoreduction coenzyme metabolic process | 8 | 2.1 | 2.0E-02 | 4.7E-03 | 2.6 | I |
| GO:0006769 | nicotinamide metabolic process | 8 | 2.1 | 2.0E-02 | 4.7E-03 | 2.6 | I |
| GO:0019318 | hexose metabolic process | 19 | 5.0 | 2.1E-02 | 9.8E-03 | 1.7 | I |
| GO:0051493 | regulation of cytoskeleton organization | 17 | 4.5 | 2.2E-02 | 9.6E-03 | 1.7 | I |
| GO:0010035 | response to inorganic substance | 18 | 4.7 | 2.4E-02 | 1.1E-02 | 1.7 | I |
| GO:0032271 | regulation of protein polymerization | 11 | 2.9 | 2.5E-02 | 8.5E-03 | 2.1 | I |
| GO:0033043 | regulation of organelle organization | 19 | 5.0 | 2.6E-02 | 1.2E-02 | 1.6 | I |
| GO:0001666 | response to hypoxia | 10 | 2.6 | 2.7E-02 | 8.7E-03 | 2.1 | I |
| GO:0045596 | negative regulation of cell differentiation | 10 | 2.6 | 2.7E-02 | 8.7E-03 | 2.1 | I |
| GO:0051960 | regulation of nervous system development | 10 | 2.6 | 2.7E-02 | 8.7E-03 | 2.1 | I |
| GO:0043488 | regulation of mRNA stability | 6 | 1.6 | 3.0E-02 | 5.3E-03 | 3.0 | I |
| GO:0043487 | regulation of RNA stability | 6 | 1.6 | 3.0E-02 | 5.3E-03 | 3.0 | I |
| GO:0010975 | regulation of neuron projection development | 7 | 1.8 | 3.1E-02 | 7.1E-03 | 2.6 | I |
| GO:0043933 | macromolecular complex subunit organization | 41 | 10.8 | 3.5E-02 | 2.3E-02 | 1.3 | I |
| GO:0070482 | response to oxygen levels | 10 | 2.6 | 3.7E-02 | 1.3E-02 | 2.0 | I |
| GO:0042592 | homeostatic process | 28 | 7.3 | 3.7E-02 | 2.2E-02 | 1.4 | I |
| GO:0065003 | macromolecular complex assembly | 39 | 10.2 | 3.9E-02 | 2.6E-02 | 1.3 | I |
| GO:0043933 | cellular macromolecular complex subunit organization | 27 | 7.1 | 4.1E-02 | 2.4E-02 | 1.4 | I |
| GO:0019748 | secondary metabolic process | 9 | 2.4 | 4.1E-02 | 1.3E-02 | 2.1 | I |
| GO:0051494 | negative regulation of cytoskeleton organization | 9 | 2.4 | 4.1E-02 | 1.3E-02 | 2.1 | I |
| GO:0010638 | positive regulation of organelle organization | 8 | 2.1 | 4.4E-02 | 1.3E-02 | 2.2 | I |
| GO:0044087 | regulation of cellular component biogenesis | 13 | 3.4 | 4.4E-02 | 1.9E-02 | 1.8 | I |
| GO:0009611 | response to wounding | 19 | 5.0 | 4.5E-02 | 2.3E-02 | 1.6 | I |
| GO:0050818 | regulation of coagulation | 5 | 1.3 | 4.7E-02 | 7.5E-03 | 3.2 | I |
| GO:0032844 | regulation of homeostatic process | 5 | 1.3 | 4.7E-02 | 7.5E-03 | 3.2 | I |
| GO:0030199 | collagen fibril organization | 7 | 1.8 | 4.8E-02 | 1.3E-02 | 2.4 | I |
| GO:0050878 | regulation of body fluid levels | 7 | 1.8 | 4.8E-02 | 1.3E-02 | 2.4 | I |
| GO:0051146 | striated muscle cell differentiation | 7 | 1.8 | 4.8E-02 | 1.3E-02 | 2.4 | I |
| GO:2000146 | negative regulation of cell motility | 7 | 1.8 | 4.8E-02 | 1.3E-02 | 2.4 | I |
| GO:0000226 | microtubule cytoskeleton organization | 10 | 2.6 | 4.9E-02 | 1.8E-02 | 2.0 | I |
| GO:0051693 | actin filament capping | 6 | 1.6 | 4.9E-02 | 1.1E-02 | 2.7 | I |
| GO:0009408 | response to heat | 6 | 1.6 | 4.9E-02 | 1.1E-02 | 2.7 | I |
| GO:0034614 | cellular response to reactive oxygen species | 6 | 1.6 | 4.9E-02 | 1.1E-02 | 2.7 | I |
| GO:0051495 | positive regulation of cytoskeleton organization | 6 | 1.6 | 4.9E-02 | 1.1E-02 | 2.7 | I |
| GO:0051130 | positive regulation of cellular component organization | 12 | 3.1 | 5.0E-02 | 2.1E-02 | 1.8 | I |
| GO:0006412 | translation | 50 | 12.5 | 5.7E-04 | 3.1E-04 | 1.5 | II |
| GO:0032270 | positive regulation of cellular protein metabolic process | 23 | 5.8 | 1.1E-03 | 4.2E-04 | 1.9 | II |
| GO:0050905 | neuromuscular process | 7 | 1.8 | 1.9E-03 | 1.5E-04 | 4.0 | II |
| GO:0031396 | regulation of protein ubiquitination | 19 | 4.8 | 2.2E-03 | 7.7E-04 | 2.0 | II |
| GO:0031401 | positive regulation of protein modification process | 21 | 5.3 | 2.4E-03 | 9.3E-04 | 1.9 | II |
| GO:0010604 | positive regulation of macromolecule metabolic process | 33 | 8.3 | 2.7E-03 | 1.4E-03 | 1.6 | II |
| GO:0006414 | translational elongation | 28 | 7.0 | 5.3E-03 | 2.6E-03 | 1.6 | II |
| GO:0050885 | neuromuscular process controlling balance | 6 | 1.5 | 6.8E-03 | 6.1E-04 | 3.9 | II |
| GO:0044092 | negative regulation of molecular function | 24 | 6.0 | 7.4E-03 | 3.4E-03 | 1.7 | II |
| GO:0019941 | modification-dependent protein catabolic process | 31 | 7.8 | 7.6E-03 | 4.0E-03 | 1.6 | II |
| GO:0043632 | modification-dependent macromolecule catabolic process | 31 | 7.8 | 7.6E-03 | 4.0E-03 | 1.6 | II |
| GO:0043086 | negative regulation of catalytic activity | 22 | 5.5 | 9.6E-03 | 4.4E-03 | 1.7 | II |
| GO:0031399 | regulation of protein modification process | 23 | 5.8 | 1.0E-02 | 4.9E-03 | 1.7 | II |
| GO:0030163 | protein catabolic process | 32 | 8.0 | 1.1E-02 | 6.1E-03 | 1.5 | II |
| GO:0006357 | regulation of transcription from RNA polymerase II promoter | 18 | 4.5 | 1.3E-02 | 5.4E-03 | 1.8 | II |
| GO:0006511 | ubiquitin-dependent protein catabolic process | 24 | 6.0 | 1.3E-02 | 6.7E-03 | 1.6 | II |
| GO:0044257 | cellular protein catabolic process | 31 | 7.8 | 1.7E-02 | 9.8E-03 | 1.5 | II |
| GO:0051603 | proteolysis involved in cellular protein catabolic process | 31 | 7.8 | 1.7E-02 | 9.8E-03 | 1.5 | II |
| GO:0022402 | cell cycle process | 34 | 8.5 | 1.8E-02 | 1.1E-02 | 1.4 | II |
| GO:0010498 | proteasomal protein catabolic process | 19 | 4.8 | 1.8E-02 | 8.1E-03 | 1.7 | II |
| GO:0043161 | proteasomal ubiquitin-dependent protein catabolic process | 19 | 4.8 | 1.8E-02 | 8.1E-03 | 1.7 | II |
| GO:0032269 | negative regulation of cellular protein metabolic process | 22 | 5.5 | 2.1E-02 | 1.1E-02 | 1.6 | II |
| GO:0032268 | regulation of cellular protein metabolic process | 33 | 8.3 | 2.1E-02 | 1.2E-02 | 1.4 | II |
| GO:0031400 | negative regulation of protein modification process | 18 | 4.5 | 3.0E-02 | 1.5E-02 | 1.6 | II |
| GO:0051248 | negative regulation of protein metabolic process | 22 | 5.5 | 3.0E-02 | 1.6E-02 | 1.5 | II |
| GO:0009057 | macromolecule catabolic process | 36 | 9.0 | 3.5E-02 | 2.2E-02 | 1.4 | II |
| GO:0044093 | positive regulation of molecular function | 31 | 7.8 | 4.5E-02 | 2.8E-02 | 1.4 | II |
| GO:0007040 | lysosome organization | 5 | 1.3 | 4.5E-02 | 6.9E-03 | 3.3 | II |
| GO:0009065 | glutamine family amino acid catabolic process | 5 | 1.3 | 4.5E-02 | 6.9E-03 | 3.3 | II |
| GO:0006508 | proteolysis | 41 | 10.3 | 4.6E-02 | 3.1E-02 | 1.3 | II |
| GO:0009063 | cellular amino acid catabolic process | 6 | 1.5 | 4.6E-02 | 9.9E-03 | 2.7 | II |
| GO:0006508 | proteolysis | 41 | 10.5 | 1.6E-02 | 1.0E-02 | 1.4 | III |
| GO:0042775 | mitochondrial ATP synthesis coupled electron transport | 8 | 2.0 | 2.0E-02 | 4.7E-03 | 2.6 | III |
| GO:0006886 | intracellular protein transport | 31 | 7.9 | 2.1E-02 | 1.3E-02 | 1.5 | III |
| GO:0046112 | nucleobase biosynthetic process | 5 | 1.3 | 3.5E-02 | 5.1E-03 | 3.5 | III |
| GO:0042440 | pigment metabolic process | 6 | 1.5 | 3.5E-02 | 7.0E-03 | 2.9 | III |
| GO:0044265 | cellular macromolecule catabolic process | 32 | 8.2 | 4.4E-02 | 2.8E-02 | 1.4 | III |
| GO:0012501 | programmed cell death | 25 | 6.6 | 3.9E-03 | 1.8E-03 | 1.8 | IV |
| GO:0010942 | positive regulation of cell death | 20 | 5.3 | 5.3E-03 | 2.3E-03 | 1.9 | IV |
| GO:0008219 | cell death | 29 | 7.7 | 5.6E-03 | 2.9E-03 | 1.6 | IV |
| GO:0006631 | fatty acid metabolic process | 11 | 2.9 | 2.5E-02 | 9.1E-03 | 2.1 | IV |
| GO:0006796 | phosphate metabolic process | 28 | 7.4 | 3.2E-02 | 1.9E-02 | 1.5 | IV |
| GO:0006915 | induction of apoptosis | 13 | 3.4 | 4.5E-02 | 2.0E-02 | 1.8 | IV |
| GO:0012502 | induction of programmed cell death | 13 | 3.4 | 4.5E-02 | 2.0E-02 | 1.8 | IV |
| GO:0022406 | membrane docking | 5 | 1.3 | 4.9E-02 | 9.2E-03 | 3.3 | IV |

**Table S4 Enrichment analysis of abundance classes of middle donor' proteins.** Enrichment analysis was carried out using DAVID and Gene Ontology biological processes. We discarded processes that had a p-value above 0.05.

| GO ID | Term | Count | % | P-Value | Fisher Exact | Fold Enrichment | Class |
| --- | --- | --- | --- | --- | --- | --- | --- |
| GO:0006414 | translational elongation | 37 | 9.4 | 2.1E-06 | 7.0E-07 | 2.1 | I |
| GO:0006457 | protein folding | 28 | 7.1 | 2.5E-05 | 7.5E-06 | 2.2 | I |
| GO:0007010 | cytoskeleton organization | 40 | 10.2 | 8.1E-05 | 3.5E-05 | 1.8 | I |
| GO:0048870 | cell motility | 30 | 7.6 | 1.4E-04 | 5.1E-05 | 1.9 | I |
| GO:0030029 | actin filament-based process | 29 | 7.4 | 2.0E-04 | 7.5E-05 | 1.9 | I |
| GO:0007015 | actin filament organization | 14 | 3.6 | 2.2E-04 | 4.1E-05 | 2.8 | I |
| GO:0030036 | actin cytoskeleton organization | 28 | 7.1 | 4.0E-04 | 1.5E-04 | 1.9 | I |
| GO:0043067 | regulation of programmed cell death | 46 | 11.7 | 7.8E-04 | 4.1E-04 | 1.6 | I |
| GO:0010941 | regulation of cell death | 46 | 11.7 | 9.5E-04 | 5.1E-04 | 1.5 | I |
| GO:0042981 | regulation of apoptosis | 45 | 11.4 | 1.1E-03 | 5.7E-04 | 1.5 | I |
| GO:0006096 | glycolysis | 13 | 3.3 | 1.8E-03 | 4.2E-04 | 2.5 | I |
| GO:0051789 | response to protein stimulus | 14 | 3.6 | 1.9E-03 | 5.0E-04 | 2.4 | I |
| GO:0006007 | glucose catabolic process | 15 | 3.8 | 2.0E-03 | 5.5E-04 | 2.3 | I |
| GO:0019320 | hexose catabolic process | 15 | 3.8 | 2.0E-03 | 5.5E-04 | 2.3 | I |
| GO:0007051 | spindle organization | 7 | 1.8 | 2.4E-03 | 1.9E-04 | 3.8 | I |
| GO:0006412 | translation | 49 | 12.4 | 2.6E-03 | 1.5E-03 | 1.5 | I |
| GO:0006986 | response to unfolded protein | 12 | 3.0 | 2.7E-03 | 6.2E-04 | 2.5 | I |
| GO:0030834 | regulation of actin filament depolymerization | 9 | 2.3 | 2.8E-03 | 4.4E-04 | 3.0 | I |
| GO:0060548 | negative regulation of cell death | 24 | 6.1 | 3.0E-03 | 1.3E-03 | 1.8 | I |
| GO:0043069 | negative regulation of programmed cell death | 24 | 6.1 | 3.0E-03 | 1.3E-03 | 1.8 | I |
| GO:0032535 | regulation of cellular component size | 20 | 5.1 | 3.2E-03 | 1.2E-03 | 1.9 | I |
| GO:0044275 | cellular carbohydrate catabolic process | 16 | 4.1 | 4.1E-03 | 1.4E-03 | 2.1 | I |
| GO:0051129 | negative regulation of cellular component organization | 16 | 4.1 | 4.1E-03 | 1.4E-03 | 2.1 | I |
| GO:0043623 | cellular protein complex assembly | 16 | 4.1 | 4.1E-03 | 1.4E-03 | 2.1 | I |
| GO:0043244 | regulation of protein complex disassembly | 12 | 3.0 | 4.3E-03 | 1.1E-03 | 2.4 | I |
| GO:0046365 | monosaccharide catabolic process | 15 | 3.8 | 4.3E-03 | 1.3E-03 | 2.1 | I |
| GO:0043066 | negative regulation of apoptosis | 23 | 5.8 | 5.4E-03 | 2.4E-03 | 1.7 | I |
| GO:0016052 | carbohydrate catabolic process | 17 | 4.3 | 5.4E-03 | 1.9E-03 | 2.0 | I |
| GO:0046164 | alcohol catabolic process | 15 | 3.8 | 6.0E-03 | 2.0E-03 | 2.1 | I |
| GO:0006006 | glucose metabolic process | 19 | 4.8 | 6.1E-03 | 2.4E-03 | 1.9 | I |
| GO:0019725 | cellular homeostasis | 24 | 6.1 | 6.2E-03 | 2.8E-03 | 1.7 | I |
| GO:0050767 | regulation of neurogenesis | 11 | 2.8 | 6.4E-03 | 1.6E-03 | 2.4 | I |
| GO:0051258 | protein polymerization | 8 | 2.0 | 7.9E-03 | 1.4E-03 | 2.9 | I |
| GO:0070271 | protein complex biogenesis | 32 | 8.1 | 8.4E-03 | 4.5E-03 | 1.5 | I |
| GO:0006461 | protein complex assembly | 32 | 8.1 | 8.4E-03 | 4.5E-03 | 1.5 | I |
| GO:0043066 | anti-apoptosis | 17 | 4.3 | 9.6E-03 | 3.8E-03 | 1.9 | I |
| GO:0030832 | regulation of actin filament length | 12 | 3.0 | 9.7E-03 | 2.9E-03 | 2.2 | I |
| GO:0008064 | regulation of actin polymerization or depolymerization | 12 | 3.0 | 9.7E-03 | 2.9E-03 | 2.2 | I |
| GO:0051960 | regulation of nervous system development | 11 | 2.8 | 9.9E-03 | 2.7E-03 | 2.3 | I |
| GO:0051493 | regulation of cytoskeleton organization | 18 | 4.6 | 1.1E-02 | 4.8E-03 | 1.8 | I |
| GO:0032970 | regulation of actin filament-based process | 13 | 3.3 | 1.3E-02 | 4.5E-03 | 2.0 | I |
| GO:0032956 | regulation of actin cytoskeleton organization | 13 | 3.3 | 1.3E-02 | 4.5E-03 | 2.0 | I |
| GO:0045454 | cell redox homeostasis | 13 | 3.3 | 1.3E-02 | 4.5E-03 | 2.0 | I |
| GO:0010033 | response to organic substance | 36 | 9.1 | 1.4E-02 | 8.2E-03 | 1.4 | I |
| GO:0010639 | negative regulation of organelle organization | 11 | 2.8 | 1.4E-02 | 4.4E-03 | 2.2 | I |
| GO:0033043 | regulation of organelle organization | 20 | 5.1 | 1.5E-02 | 6.8E-03 | 1.7 | I |
| GO:0042592 | homeostatic process | 30 | 7.6 | 1.5E-02 | 8.2E-03 | 1.5 | I |
| GO:0043242 | negative regulation of protein complex disassembly | 9 | 2.3 | 1.5E-02 | 3.6E-03 | 2.5 | I |
| GO:0045664 | regulation of neuron differentiation | 9 | 2.3 | 1.5E-02 | 3.6E-03 | 2.5 | I |
| GO:0010638 | positive regulation of organelle organization | 9 | 2.3 | 1.5E-02 | 3.6E-03 | 2.5 | I |
| GO:0000226 | microtubule cytoskeleton organization | 11 | 2.8 | 2.1E-02 | 6.7E-03 | 2.1 | I |
| GO:0030835 | negative regulation of actin filament depolymerization | 7 | 1.8 | 2.1E-02 | 4.2E-03 | 2.8 | I |
| GO:0008015 | blood circulation | 10 | 2.5 | 2.2E-02 | 6.6E-03 | 2.2 | I |
| GO:0003013 | circulatory system process | 10 | 2.5 | 2.2E-02 | 6.6E-03 | 2.2 | I |
| GO:0009266 | response to temperature stimulus | 8 | 2.0 | 2.2E-02 | 5.4E-03 | 2.5 | I |
| GO:0051130 | positive regulation of cellular component organization | 13 | 3.3 | 2.4E-02 | 9.1E-03 | 1.9 | I |
| GO:0019318 | hexose metabolic process | 19 | 4.8 | 2.6E-02 | 1.2E-02 | 1.6 | I |
| GO:0032271 | regulation of protein polymerization | 11 | 2.8 | 2.8E-02 | 9.9E-03 | 2.0 | I |
| GO:0010035 | response to inorganic substance | 18 | 4.6 | 2.9E-02 | 1.4E-02 | 1.6 | I |
| GO:0043933 | macromolecular complex subunit organization | 42 | 10.7 | 3.0E-02 | 2.0E-02 | 1.3 | I |
| GO:0043933 | cellular macromolecular complex subunit organization | 28 | 7.1 | 3.0E-02 | 1.7E-02 | 1.4 | I |
| GO:0001666 | response to hypoxia | 10 | 2.5 | 3.1E-02 | 1.0E-02 | 2.1 | I |
| GO:0045596 | negative regulation of cell differentiation | 10 | 2.5 | 3.1E-02 | 1.0E-02 | 2.1 | I |
| GO:0043488 | regulation of mRNA stability | 6 | 1.5 | 3.3E-02 | 5.9E-03 | 2.9 | I |
| GO:0043487 | regulation of RNA stability | 6 | 1.5 | 3.3E-02 | 5.9E-03 | 2.9 | I |
| GO:0065003 | macromolecular complex assembly | 40 | 10.2 | 3.4E-02 | 2.2E-02 | 1.3 | I |
| GO:0010975 | regulation of neuron projection development | 7 | 1.8 | 3.4E-02 | 8.0E-03 | 2.6 | I |
| GO:0060284 | regulation of cell development | 11 | 2.8 | 3.8E-02 | 1.4E-02 | 1.9 | I |
| GO:0034622 | cellular macromolecular complex assembly | 25 | 6.3 | 4.2E-02 | 2.4E-02 | 1.4 | I |
| GO:0070482 | response to oxygen levels | 10 | 2.5 | 4.2E-02 | 1.5E-02 | 2.0 | I |
| GO:0051235 | maintenance of location | 9 | 2.3 | 4.6E-02 | 1.5E-02 | 2.1 | I |
| GO:0051494 | negative regulation of cytoskeleton organization | 9 | 2.3 | 4.6E-02 | 1.5E-02 | 2.1 | I |
| GO:0032268 | regulation of cellular protein metabolic process | 41 | 10.0 | 1.8E-04 | 8.3E-05 | 1.7 | II |
| GO:0031398 | positive regulation of protein ubiquitination | 21 | 5.1 | 2.9E-04 | 8.7E-05 | 2.2 | II |
| GO:0006412 | translation | 52 | 12.7 | 4.5E-04 | 2.4E-04 | 1.5 | II |
| GO:0051247 | positive regulation of protein metabolic process | 25 | 6.1 | 5.3E-04 | 1.9E-04 | 2.0 | II |
| GO:0031401 | positive regulation of protein modification process | 23 | 5.6 | 5.9E-04 | 2.0E-04 | 2.0 | II |
| GO:0051444 | negative regulation of ubiquitin-protein ligase activity | 19 | 4.7 | 6.4E-04 | 1.9E-04 | 2.2 | II |
| GO:0051352 | negative regulation of ligase activity | 19 | 4.7 | 6.4E-04 | 1.9E-04 | 2.2 | II |
| GO:0031397 | negative regulation of protein ubiquitination | 19 | 4.7 | 6.4E-04 | 1.9E-04 | 2.2 | II |
| GO:0031145 | anaphase-promoting complex-dependent proteasomal ubiquitin-dependent protein catabolic process | 19 | 4.7 | 6.4E-04 | 1.9E-04 | 2.2 | II |
| GO:0051436 | negative regulation of ubiquitin-protein ligase activity during mitotic cell cycle | 19 | 4.7 | 6.4E-04 | 1.9E-04 | 2.2 | II |
| GO:0031399 | regulation of protein modification process | 27 | 6.6 | 6.4E-04 | 2.5E-04 | 1.9 | II |
| GO:0032269 | negative regulation of cellular protein metabolic process | 26 | 6.4 | 1.6E-03 | 6.7E-04 | 1.8 | II |
| GO:0019941 | modification-dependent protein catabolic process | 34 | 8.3 | 1.9E-03 | 9.1E-04 | 1.6 | II |
| GO:0043632 | modification-dependent macromolecule catabolic process | 34 | 8.3 | 1.9E-03 | 9.1E-04 | 1.6 | II |
| GO:0051248 | negative regulation of protein metabolic process | 26 | 6.4 | 2.7E-03 | 1.2E-03 | 1.7 | II |
| GO:0010604 | positive regulation of macromolecule metabolic process | 34 | 8.3 | 2.8E-03 | 1.4E-03 | 1.6 | II |
| GO:0030163 | protein catabolic process | 35 | 8.6 | 3.1E-03 | 1.6E-03 | 1.6 | II |
| GO:0031400 | negative regulation of protein modification process | 21 | 5.1 | 4.2E-03 | 1.7E-03 | 1.8 | II |
| GO:0009057 | macromolecule catabolic process | 41 | 10.0 | 4.6E-03 | 2.6E-03 | 1.5 | II |
| GO:0044265 | cellular macromolecule catabolic process | 39 | 9.6 | 4.9E-03 | 2.7E-03 | 1.5 | II |
| GO:0051603 | proteolysis involved in cellular protein catabolic process | 34 | 8.3 | 5.1E-03 | 2.7E-03 | 1.5 | II |
| GO:0044257 | cellular protein catabolic process | 34 | 8.3 | 5.1E-03 | 2.7E-03 | 1.5 | II |
| GO:0006511 | ubiquitin-dependent protein catabolic process | 26 | 6.4 | 5.4E-03 | 2.5E-03 | 1.7 | II |
| GO:0044092 | negative regulation of molecular function | 25 | 6.1 | 6.0E-03 | 2.8E-03 | 1.7 | II |
| GO:0043086 | negative regulation of catalytic activity | 23 | 5.6 | 7.4E-03 | 3.4E-03 | 1.7 | II |
| GO:0006414 | translational elongation | 28 | 6.9 | 9.9E-03 | 5.1E-03 | 1.6 | II |
| GO:0043161 | proteasomal ubiquitin-dependent protein catabolic process | 20 | 4.9 | 1.3E-02 | 5.7E-03 | 1.7 | II |
| GO:0010498 | proteasomal protein catabolic process | 20 | 4.9 | 1.3E-02 | 5.7E-03 | 1.7 | II |
| GO:0010605 | negative regulation of macromolecule metabolic process | 37 | 9.1 | 1.4E-02 | 8.1E-03 | 1.4 | II |
| GO:0003006 | reproductive developmental process | 10 | 2.5 | 1.5E-02 | 4.3E-03 | 2.3 | II |
| GO:0050905 | neuromuscular process | 6 | 1.5 | 1.8E-02 | 2.5E-03 | 3.3 | II |
| GO:0043085 | positive regulation of catalytic activity | 30 | 7.4 | 1.9E-02 | 1.1E-02 | 1.5 | II |
| GO:0009310 | amine catabolic process | 7 | 1.7 | 2.2E-02 | 4.3E-03 | 2.8 | II |
| GO:0006508 | proteolysis | 44 | 10.8 | 2.4E-02 | 1.6E-02 | 1.3 | II |
| GO:0044093 | positive regulation of molecular function | 33 | 8.1 | 2.8E-02 | 1.7E-02 | 1.4 | II |
| GO:0006413 | translational initiation | 10 | 2.5 | 3.2E-02 | 1.0E-02 | 2.1 | II |
| GO:0022402 | cell cycle process | 34 | 8.3 | 3.3E-02 | 2.1E-02 | 1.4 | II |
| GO:0048610 | reproductive cellular process | 8 | 2.0 | 3.5E-02 | 9.6E-03 | 2.3 | II |
| GO:0006357 | regulation of transcription from RNA polymerase II promoter | 17 | 4.2 | 4.1E-02 | 2.0E-02 | 1.6 | II |
| GO:0034660 | ncRNA metabolic process | 17 | 4.2 | 5.0E-02 | 2.5E-02 | 1.6 | II |
| GO:0042775 | mitochondrial ATP synthesis coupled electron transport | 9 | 2.4 | 3.4E-03 | 6.1E-04 | 3.1 | III |
| GO:0045333 | cellular respiration | 14 | 3.7 | 1.0E-02 | 3.8E-03 | 2.1 | III |
| GO:0015980 | energy derivation by oxidation of organic compounds | 17 | 4.5 | 1.5E-02 | 6.4E-03 | 1.8 | III |
| GO:0006120 | mitochondrial electron transport. NADH to ubiquinone | 7 | 1.8 | 2.6E-02 | 5.9E-03 | 2.8 | III |
| GO:0046907 | intracellular transport | 47 | 12.3 | 2.7E-02 | 1.8E-02 | 1.3 | III |
| GO:0042440 | pigment metabolic process | 6 | 1.6 | 2.9E-02 | 5.4E-03 | 3.1 | III |
| GO:0046112 | nucleobase biosynthetic process | 5 | 1.3 | 3.0E-02 | 4.1E-03 | 3.7 | III |
| GO:0006886 | intracellular protein transport | 29 | 7.6 | 3.4E-02 | 2.1E-02 | 1.4 | III |
| GO:0070727 | cellular macromolecule localization | 30 | 7.9 | 3.7E-02 | 2.2E-02 | 1.4 | III |
| GO:0033365 | protein localization in organelle | 12 | 3.1 | 4.1E-02 | 1.7E-02 | 1.9 | III |
| GO:0006913 | nucleocytoplasmic transport | 14 | 3.7 | 4.1E-02 | 1.8E-02 | 1.8 | III |
| GO:0051169 | nuclear transport | 14 | 3.7 | 4.1E-02 | 1.8E-02 | 1.8 | III |
| GO:0012501 | programmed cell death | 24 | 6.6 | 5.9E-03 | 2.8E-03 | 1.7 | IV |
| GO:0008219 | cell death | 28 | 7.7 | 7.5E-03 | 3.9E-03 | 1.6 | IV |
| GO:0006631 | fatty acid metabolic process | 11 | 3.0 | 2.1E-02 | 7.4E-03 | 2.2 | IV |
| GO:0006796 | phosphate metabolic process | 27 | 7.4 | 4.0E-02 | 2.4E-02 | 1.4 | IV |

**Table S5 Enrichment analysis of abundance classes of old donor' proteins.** Enrichment analysis was carried out using DAVID and Gene Ontology biological processes. We discarded processes that had a p-value above 0.05.

| GO ID | Term | Count | % | P-Value | Fisher Exact | Fold Enrichment | Class |
| --- | --- | --- | --- | --- | --- | --- | --- |
| GO:0006414 | translational elongation | 37 | 9.1 | 5.1E-06 | 1.7E-06 | 2.0 | I |
| GO:0006457 | protein folding | 28 | 6.9 | 4.7E-05 | 1.5E-05 | 2.1 | I |
| GO:0007010 | cytoskeleton organization | 41 | 10.1 | 7.5E-05 | 3.2E-05 | 1.8 | I |
| GO:0030029 | actin filament-based process | 30 | 7.4 | 1.4E-04 | 5.0E-05 | 1.9 | I |
| GO:0048870 | cell motility | 30 | 7.4 | 2.6E-04 | 1.0E-04 | 1.9 | I |
| GO:0030036 | actin cytoskeleton organization | 29 | 7.1 | 2.7E-04 | 1.0E-04 | 1.9 | I |
| GO:0007015 | actin filament organization | 14 | 3.4 | 3.2E-04 | 6.1E-05 | 2.7 | I |
| GO:0051129 | negative regulation of cellular component organization | 18 | 4.4 | 5.8E-04 | 1.6E-04 | 2.3 | I |
| GO:0019320 | hexose catabolic process | 16 | 3.9 | 7.8E-04 | 2.0E-04 | 2.3 | I |
| GO:0006007 | glucose catabolic process | 16 | 3.9 | 7.8E-04 | 2.0E-04 | 2.3 | I |
| GO:0043067 | regulation of programmed cell death | 47 | 11.5 | 8.7E-04 | 4.6E-04 | 1.5 | I |
| GO:0010941 | regulation of cell death | 47 | 11.5 | 1.1E-03 | 5.7E-04 | 1.5 | I |
| GO:0042981 | regulation of apoptosis | 46 | 11.3 | 1.2E-03 | 6.3E-04 | 1.5 | I |
| GO:0010639 | negative regulation of organelle organization | 13 | 3.2 | 1.5E-03 | 3.3E-04 | 2.5 | I |
| GO:0043244 | regulation of protein complex disassembly | 13 | 3.2 | 1.5E-03 | 3.3E-04 | 2.5 | I |
| GO:0006412 | translation | 51 | 12.5 | 1.7E-03 | 9.8E-04 | 1.5 | I |
| GO:0032535 | regulation of cellular component size | 21 | 5.2 | 1.8E-03 | 6.7E-04 | 1.9 | I |
| GO:0044275 | cellular carbohydrate catabolic process | 17 | 4.2 | 1.9E-03 | 6.0E-04 | 2.1 | I |
| GO:0046365 | monosaccharide catabolic process | 16 | 3.9 | 1.9E-03 | 5.6E-04 | 2.2 | I |
| GO:0060548 | negative regulation of cell death | 25 | 6.1 | 2.0E-03 | 8.3E-04 | 1.8 | I |
| GO:0043069 | negative regulation of programmed cell death | 25 | 6.1 | 2.0E-03 | 8.3E-04 | 1.8 | I |
| GO:0006096 | glycolysis | 13 | 3.2 | 2.4E-03 | 6.0E-04 | 2.4 | I |
| GO:0051789 | response to protein stimulus | 14 | 3.4 | 2.6E-03 | 7.2E-04 | 2.3 | I |
| GO:0051493 | regulation of cytoskeleton organization | 20 | 4.9 | 2.6E-03 | 9.6E-04 | 1.9 | I |
| GO:0046164 | alcohol catabolic process | 16 | 3.9 | 2.8E-03 | 8.7E-04 | 2.1 | I |
| GO:0016052 | carbohydrate catabolic process | 18 | 4.4 | 2.8E-03 | 9.5E-04 | 2.0 | I |
| GO:0007051 | spindle organization | 7 | 1.7 | 2.9E-03 | 2.4E-04 | 3.7 | I |
| GO:0030834 | regulation of actin filament depolymerization | 9 | 2.2 | 3.5E-03 | 5.7E-04 | 2.9 | I |
| GO:0006006 | glucose metabolic process | 20 | 4.9 | 3.6E-03 | 1.4E-03 | 1.9 | I |
| GO:0006986 | response to unfolded protein | 12 | 2.9 | 3.6E-03 | 8.7E-04 | 2.4 | I |
| GO:0001666 | response to hypoxia | 12 | 2.9 | 3.6E-03 | 8.7E-04 | 2.4 | I |
| GO:0043066 | negative regulation of apoptosis | 24 | 5.9 | 3.7E-03 | 1.6E-03 | 1.8 | I |
| GO:0030832 | regulation of actin filament length | 13 | 3.2 | 3.8E-03 | 1.0E-03 | 2.3 | I |
| GO:0008064 | regulation of actin polymerization or depolymerization | 13 | 3.2 | 3.8E-03 | 1.0E-03 | 2.3 | I |
| GO:0033043 | regulation of organelle organization | 22 | 5.4 | 4.2E-03 | 1.7E-03 | 1.8 | I |
| GO:0019725 | cellular homeostasis | 25 | 6.1 | 4.4E-03 | 2.0E-03 | 1.7 | I |
| GO:0043242 | negative regulation of protein complex disassembly | 10 | 2.5 | 4.5E-03 | 9.2E-04 | 2.7 | I |
| GO:0051494 | negative regulation of cytoskeleton organization | 11 | 2.7 | 5.2E-03 | 1.2E-03 | 2.5 | I |
| GO:0043066 | anti-apoptosis | 18 | 4.4 | 5.3E-03 | 2.0E-03 | 1.9 | I |
| GO:0070482 | response to oxygen levels | 12 | 2.9 | 5.6E-03 | 1.5E-03 | 2.3 | I |
| GO:0032956 | regulation of actin cytoskeleton organization | 14 | 3.4 | 5.9E-03 | 1.9E-03 | 2.1 | I |
| GO:0032970 | regulation of actin filament-based process | 14 | 3.4 | 5.9E-03 | 1.9E-03 | 2.1 | I |
| GO:0008015 | blood circulation | 11 | 2.7 | 8.3E-03 | 2.2E-03 | 2.3 | I |
| GO:0003013 | circulatory system process | 11 | 2.7 | 8.3E-03 | 2.2E-03 | 2.3 | I |
| GO:0032271 | regulation of protein polymerization | 12 | 2.9 | 1.3E-02 | 3.9E-03 | 2.1 | I |
| GO:0042592 | homeostatic process | 31 | 7.6 | 1.3E-02 | 7.1E-03 | 1.5 | I |
| GO:0045596 | negative regulation of cell differentiation | 11 | 2.7 | 1.3E-02 | 3.6E-03 | 2.2 | I |
| GO:0034614 | cellular response to reactive oxygen species | 7 | 1.7 | 1.4E-02 | 2.3E-03 | 3.0 | I |
| GO:0070271 | protein complex biogenesis | 32 | 7.9 | 1.4E-02 | 7.7E-03 | 1.5 | I |
| GO:0006461 | protein complex assembly | 32 | 7.9 | 1.4E-02 | 7.7E-03 | 1.5 | I |
| GO:0043623 | cellular protein complex assembly | 15 | 3.7 | 1.5E-02 | 5.9E-03 | 1.9 | I |
| GO:0045454 | cell redox homeostasis | 13 | 3.2 | 1.7E-02 | 6.1E-03 | 2.0 | I |
| GO:0019318 | hexose metabolic process | 20 | 4.9 | 1.7E-02 | 7.9E-03 | 1.7 | I |
| GO:0010638 | positive regulation of organelle organization | 9 | 2.2 | 1.8E-02 | 4.6E-03 | 2.4 | I |
| GO:0045664 | regulation of neuron differentiation | 9 | 2.2 | 1.8E-02 | 4.6E-03 | 2.4 | I |
| GO:0051235 | maintenance of location | 10 | 2.5 | 1.8E-02 | 5.3E-03 | 2.2 | I |
| GO:0010035 | response to inorganic substance | 19 | 4.7 | 1.9E-02 | 8.5E-03 | 1.7 | I |
| GO:0010033 | response to organic substance | 36 | 8.8 | 2.3E-02 | 1.4E-02 | 1.4 | I |
| GO:0030835 | negative regulation of actin filament depolymerization | 7 | 1.7 | 2.5E-02 | 5.1E-03 | 2.7 | I |
| GO:0000226 | microtubule cytoskeleton organization | 11 | 2.7 | 2.6E-02 | 8.8E-03 | 2.0 | I |
| GO:0000302 | response to reactive oxygen species | 10 | 2.5 | 2.7E-02 | 8.5E-03 | 2.1 | I |
| GO:0030833 | regulation of actin filament polymerization | 10 | 2.5 | 2.7E-02 | 8.5E-03 | 2.1 | I |
| GO:0050767 | regulation of neurogenesis | 10 | 2.5 | 2.7E-02 | 8.5E-03 | 2.1 | I |
| GO:0006769 | nicotinamide metabolic process | 8 | 2.0 | 2.7E-02 | 6.7E-03 | 2.4 | I |
| GO:0046496 | nicotinamide nucleotide metabolic process | 8 | 2.0 | 2.7E-02 | 6.7E-03 | 2.4 | I |
| GO:0009820 | alkaloid metabolic process | 8 | 2.0 | 2.7E-02 | 6.7E-03 | 2.4 | I |
| GO:0006733 | oxidoreduction coenzyme metabolic process | 8 | 2.0 | 2.7E-02 | 6.7E-03 | 2.4 | I |
| GO:0019362 | pyridine nucleotide metabolic process | 8 | 2.0 | 2.7E-02 | 6.7E-03 | 2.4 | I |
| GO:0009266 | response to temperature stimulus | 8 | 2.0 | 2.7E-02 | 6.7E-03 | 2.4 | I |
| GO:0044087 | regulation of cellular component biogenesis | 14 | 3.4 | 2.8E-02 | 1.2E-02 | 1.8 | I |
| GO:0051130 | positive regulation of cellular component organization | 13 | 3.2 | 3.1E-02 | 1.2E-02 | 1.8 | I |
| GO:0043254 | regulation of protein complex assembly | 12 | 2.9 | 3.3E-02 | 1.3E-02 | 1.9 | I |
| GO:0043933 | macromolecular complex subunit organization | 43 | 10.6 | 3.3E-02 | 2.2E-02 | 1.3 | I |
| GO:0065003 | macromolecular complex assembly | 41 | 10.1 | 3.5E-02 | 2.3E-02 | 1.3 | I |
| GO:0043488 | regulation of mRNA stability | 6 | 1.5 | 3.7E-02 | 7.1E-03 | 2.8 | I |
| GO:0043487 | regulation of RNA stability | 6 | 1.5 | 3.7E-02 | 7.1E-03 | 2.8 | I |
| GO:0042274 | ribosomal small subunit biogenesis | 6 | 1.5 | 3.7E-02 | 7.1E-03 | 2.8 | I |
| GO:0042743 | hydrogen peroxide metabolic process | 6 | 1.5 | 3.7E-02 | 7.1E-03 | 2.8 | I |
| GO:0051960 | regulation of nervous system development | 10 | 2.5 | 3.8E-02 | 1.3E-02 | 2.0 | I |
| GO:0009611 | response to wounding | 20 | 4.9 | 3.8E-02 | 2.0E-02 | 1.5 | I |
| GO:0051258 | protein polymerization | 7 | 1.7 | 4.0E-02 | 9.8E-03 | 2.5 | I |
| GO:0010975 | regulation of neuron projection development | 7 | 1.7 | 4.0E-02 | 9.8E-03 | 2.5 | I |
| GO:0030837 | negative regulation of actin filament polymerization | 7 | 1.7 | 4.0E-02 | 9.8E-03 | 2.5 | I |
| GO:0043933 | cellular macromolecular complex subunit organization | 28 | 6.9 | 4.5E-02 | 2.7E-02 | 1.4 | I |
| GO:0005996 | monosaccharide metabolic process | 20 | 4.9 | 4.6E-02 | 2.4E-02 | 1.5 | I |
| GO:0006412 | translation | 51 | 12.5 | 7.5E-04 | 4.1E-04 | 1.5 | II |
| GO:0032270 | positive regulation of cellular protein metabolic process | 23 | 5.6 | 1.9E-03 | 7.4E-04 | 1.9 | II |
| GO:0031396 | regulation of protein ubiquitination | 19 | 4.6 | 3.4E-03 | 1.3E-03 | 1.9 | II |
| GO:0031399 | regulation of protein modification process | 25 | 6.1 | 3.5E-03 | 1.5E-03 | 1.7 | II |
| GO:0006414 | translational elongation | 28 | 6.8 | 9.2E-03 | 4.7E-03 | 1.6 | II |
| GO:0044092 | negative regulation of molecular function | 24 | 5.9 | 1.2E-02 | 5.8E-03 | 1.6 | II |
| GO:0051248 | negative regulation of protein metabolic process | 24 | 5.9 | 1.2E-02 | 5.8E-03 | 1.6 | II |
| GO:0003006 | reproductive developmental process | 10 | 2.4 | 1.5E-02 | 4.1E-03 | 2.3 | II |
| GO:0043086 | negative regulation of catalytic activity | 22 | 5.4 | 1.5E-02 | 7.1E-03 | 1.6 | II |
| GO:0050905 | neuromuscular process | 6 | 1.5 | 1.8E-02 | 2.4E-03 | 3.3 | II |
| GO:0006511 | ubiquitin-dependent protein catabolic process | 24 | 5.9 | 2.1E-02 | 1.1E-02 | 1.6 | II |
| GO:0031400 | negative regulation of protein modification process | 19 | 4.6 | 2.1E-02 | 9.7E-03 | 1.7 | II |
| GO:0019941 | modification-dependent protein catabolic process | 30 | 7.3 | 2.4E-02 | 1.4E-02 | 1.5 | II |
| GO:0043632 | modification-dependent macromolecule catabolic process | 30 | 7.3 | 2.4E-02 | 1.4E-02 | 1.5 | II |
| GO:0030163 | protein catabolic process | 31 | 7.6 | 3.3E-02 | 2.0E-02 | 1.4 | II |
| GO:0048610 | reproductive cellular process | 8 | 2.0 | 3.4E-02 | 9.3E-03 | 2.3 | II |
| GO:0010605 | negative regulation of macromolecule metabolic process | 35 | 8.6 | 3.6E-02 | 2.2E-02 | 1.4 | II |
| GO:0009057 | macromolecule catabolic process | 37 | 9.0 | 3.7E-02 | 2.3E-02 | 1.3 | II |
| GO:0044265 | cellular macromolecule catabolic process | 35 | 8.6 | 4.0E-02 | 2.6E-02 | 1.4 | II |
| GO:0044257 | cellular protein catabolic process | 30 | 7.3 | 4.8E-02 | 3.0E-02 | 1.4 | II |
| GO:0051603 | proteolysis involved in cellular protein catabolic process | 30 | 7.3 | 4.8E-02 | 3.0E-02 | 1.4 | II |
| GO:0022402 | cell cycle process | 33 | 8.1 | 5.0E-02 | 3.2E-02 | 1.3 | II |
| GO:0042440 | pigment metabolic process | 6 | 1.6 | 2.5E-02 | 4.6E-03 | 3.2 | III |
| GO:0046112 | nucleobase biosynthetic process | 5 | 1.4 | 2.7E-02 | 3.5E-03 | 3.8 | III |
| GO:0046907 | intracellular transport | 45 | 12.2 | 3.8E-02 | 2.6E-02 | 1.3 | III |
| GO:0046148 | pigment biosynthetic process | 5 | 1.4 | 4.6E-02 | 8.0E-03 | 3.3 | III |
| GO:0006119 | oxidative phosphorylation | 13 | 3.6 | 5.1E-03 | 1.6E-03 | 2.3 | IV |
| GO:0042775 | mitochondrial ATP synthesis coupled electron transport | 8 | 2.2 | 5.6E-03 | 1.0E-03 | 3.3 | IV |
| GO:0006796 | phosphate metabolic process | 30 | 8.3 | 6.2E-03 | 3.3E-03 | 1.6 | IV |
| GO:0006631 | fatty acid metabolic process | 12 | 3.3 | 6.9E-03 | 2.1E-03 | 2.4 | IV |
| GO:0016310 | phosphorylation | 25 | 6.9 | 7.9E-03 | 3.9E-03 | 1.7 | IV |
| GO:0006120 | mitochondrial electron transport. NADH to ubiquinone | 7 | 1.9 | 1.1E-02 | 2.0E-03 | 3.3 | IV |
| GO:0022904 | respiratory electron transport chain | 8 | 2.2 | 1.8E-02 | 4.5E-03 | 2.7 | IV |
| GO:0008219 | cell death | 26 | 7.2 | 2.5E-02 | 1.4E-02 | 1.5 | IV |
| GO:0016265 | death | 26 | 7.2 | 2.5E-02 | 1.4E-02 | 1.5 | IV |
| GO:0012501 | programmed cell death | 22 | 6.1 | 3.4E-02 | 1.9E-02 | 1.5 | IV |
| GO:0001889 | liver development | 5 | 1.4 | 4.4E-02 | 8.1E-03 | 3.4 | IV |

**Table S6 Enrichment analysis of age-associated decreasing (14) and increasing (9) mitochondrial proteins obtained from cluster analysis (p ≤ 0.1).** Enrichment analysis was carried out using DAVID and Gene Ontology biological processes. We discarded processes that had a p-value above 0.05. A total of 19 biological processes were significantly decreased and 16 biological processes were significantly increased with age.

| GO ID | GO Term | Count | % | p-value | Regulation |
| --- | --- | --- | --- | --- | --- |
| GO:0006091 | generation of precursor metabolites and energy | 8 | 57.1 | 3.80E-09 | down |
| GO:0022900 | electron transport chain | 6 | 42.9 | 3.80E-08 | down |
| GO:0055114 | oxidation reduction | 8 | 57.1 | 5.10E-07 | down |
| GO:0006119 | oxidative phosphorylation | 5 | 35.7 | 1.50E-06 | down |
| GO:0042773 | ATP synthesis coupled electron transport | 4 | 28.6 | 1.60E-05 | down |
| GO:0042775 | mitochondrial ATP synthesis coupled electron transport | 4 | 28.6 | 1.60E-05 | down |
| GO:0022904 | respiratory electron transport chain | 4 | 28.6 | 2.50E-05 | down |
| GO:0045333 | cellular respiration | 4 | 28.6 | 8.60E-05 | down |
| GO:0015980 | energy derivation by oxidation of organic compounds | 4 | 28.6 | 2.80E-04 | down |
| GO:0008152 | metabolic process | 14 | 100.0 | 3.40E-04 | down |
| GO:0006120 | mitochondrial electron transport, NADH to ubiquinone | 3 | 21.4 | 6.60E-04 | down |
| GO:0044237 | cellular metabolic process | 13 | 92.9 | 8.50E-04 | down |
| GO:0019752 | carboxylic acid metabolic process | 5 | 35.7 | 1.30E-03 | down |
| GO:0043436 | oxoacid metabolic process | 5 | 35.7 | 1.30E-03 | down |
| GO:0006082 | organic acid metabolic process | 5 | 35.7 | 1.30E-03 | down |
| GO:0042180 | cellular ketone metabolic process | 5 | 35.7 | 1.40E-03 | down |
| GO:0006810 | transport | 8 | 57.1 | 4.60E-03 | down |
| GO:0016310 | phosphorylation | 5 | 35.7 | 4.80E-03 | down |
| GO:0051234 | establishment of localization | 8 | 57.1 | 4.90E-03 | down |
| GO:0042221 | response to chemical stimulus | 5 | 50.0 | 5.90E-03 | up |
| GO:0010035 | response to inorganic substance | 3 | 30.0 | 7.10E-03 | up |
| GO:0010033 | response to organic substance | 4 | 40.0 | 8.80E-03 | up |
| GO:0003006 | reproductive developmental process | 3 | 30.0 | 1.10E-02 | up |
| GO:0006950 | response to stress | 5 | 50.0 | 1.60E-02 | up |
| GO:0051591 | response to cAMP | 2 | 20.0 | 2.60E-02 | up |
| GO:0007286 | spermatid development | 2 | 20.0 | 3.40E-02 | up |
| GO:0048515 | spermatid differentiation | 2 | 20.0 | 3.60E-02 | up |
| GO:0009056 | catabolic process | 4 | 40.0 | 3.90E-02 | up |
| GO:0006401 | RNA catabolic process | 2 | 20.0 | 4.10E-02 | up |
| GO:0006986 | response to unfolded protein | 2 | 20.0 | 4.40E-02 | up |
| GO:0019752 | carboxylic acid metabolic process | 3 | 30.0 | 4.60E-02 | up |
| GO:0043436 | oxoacid metabolic process | 3 | 30.0 | 4.60E-02 | up |
| GO:0006082 | organic acid metabolic process | 3 | 30.0 | 4.70E-02 | up |
| GO:0050896 | response to stimulus | 6 | 60.0 | 4.70E-02 | up |
| GO:0042180 | cellular ketone metabolic process | 3 | 30.0 | 4.80E-02 | up |

**Table S7 Significantly altered genes with age published by Kalfalah *et al*. [10].** Statistical analysis of transcriptome data revealed 137 significantly altered genes with age.

| EntrezID | UniProt AC | Gene Symbol | Gene Name | robust p-value | robust q-value |
| --- | --- | --- | --- | --- | --- |
| 340340 | A4D0Q7 | LOC340340 | uncharacterized LOC340340 | 2.78E-05 | 9.59E-03 |
| 161247 | A5D6W6 | FITM1 | fat storage-inducing transmembrane protein 1 | 7.23E-04 | 8.07E-02 |
| 100505591 | A6NJW4 | LRRC3C | leucine rich repeat containing 3C | 1.26E-04 | 2.53E-02 |
| 342931 | A6NLU0 | RFPL4A | ret finger protein-like 4A | 1.95E-04 | 3.40E-02 |
| 23015 | A7E2F4 | GOLGA8A | golgin A8 family, member A | 7.23E-05 | 1.79E-02 |
| 646174 | A8MZG2 | C16orf90 | chromosome 16 open reading frame 90 | 8.10E-05 | 1.96E-02 |
| 1238 | O00590 | CCBP2 | chemokine binding protein 2 | 8.39E-04 | 8.99E-02 |
| 4593 | O15146 | MUSK | muscle, skeletal, receptor tyrosine kinase | 2.21E-06 | 1.60E-03 |
| 1592 | O43174 | CYP26A1 | cytochrome P450, family 26, subfamily A, polypeptide 1 | 2.56E-04 | 3.98E-02 |
| 50846 | O43323 | DHH | desert hedgehog | 9.82E-05 | 2.11E-02 |
| 23520 | O43423 | ANP32C | acidic (leucine-rich) nuclear phosphoprotein 32 family, member C | 1.90E-14 | 4.12E-10 |
| 9506 | O60829 | PAGE4 | P antigen family, member 4 (prostate associated) | 1.87E-07 | 2.40E-04 |
| 1089 | O75871 | CEACAM4 | carcinoembryonic antigen-related cell adhesion molecule 4 | 5.05E-04 | 6.50E-02 |
| 11258 | O75935 | DCTN3 | dynactin 3 (p22) | 1.97E-04 | 3.40E-02 |
| 1797 | O77932 | DOM3Z | dom-3 homolog Z (C. elegans) | 1.17E-07 | 1.69E-04 |
| 343 | O94778 | AQP8 | aquaporin 8 | 9.49E-05 | 2.08E-02 |
| 8723 | O95219 | SNX4 | sorting nexin 4 | 5.88E-05 | 1.57E-02 |
| 3514 | P01834 | IGKC | immunoglobulin kappa constant | 9.42E-04 | 9.76E-02 |
| 3119 | P01920 | HLA-DQB1 | major histocompatibility complex, class II, DQ beta 1 | 8.40E-05 | 1.99E-02 |
| 2638 | P02774 | GC | group-specific component (vitamin D binding protein) | 5.86E-04 | 6.93E-02 |
| 7031 | P04155 | TFF1 | trefoil factor 1 | 6.45E-04 | 7.54E-02 |
| 6343 | P09683 | SCT | secretin | 4.76E-04 | 6.31E-02 |
| 34 | P11310 | ACADM | acyl-CoA dehydrogenase, C-4 to C-12 straight chain | 7.50E-04 | 8.32E-02 |
| 2184 | P16930 | FAH | fumarylacetoacetate hydrolase (fumarylacetoacetase) | 1.60E-04 | 3.03E-02 |
| 23433 | P17081 | RHOQ | ras homolog family member Q | 7.08E-04 | 7.98E-02 |
| 6609 | P17405 | SMPD1 | sphingomyelin phosphodiesterase 1, acid lysosomal | 2.47E-04 | 3.92E-02 |
| 3727 | P17535 | JUND | jun D proto-oncogene | 8.83E-05 | 2.02E-02 |
| 5046 | P29122 | PCSK6 | proprotein convertase subtilisin/kexin type 6 | 1.14E-05 | 5.37E-03 |
| 795 | P29377 | S100G | S100 calcium binding protein G | 5.15E-04 | 6.51E-02 |
| 3682 | P38570 | ITGAE | integrin, alpha E (antigen CD103, human mucosal lymphocyte antigen 1; alpha polypeptide) | 3.87E-05 | 1.15E-02 |
| 9775 | P38919 | EIF4A3 | eukaryotic translation initiation factor 4A3 | 1.53E-08 | 3.33E-05 |
| 6506 | P43004 | SLC1A2 | solute carrier family 1 (glial high affinity glutamate transporter), member 2 | 9.65E-07 | 8.39E-04 |
| 4103 | P43358 | MAGEA4 | melanoma antigen family A, 4 | 1.41E-06 | 1.13E-03 |
| 7080 | P43699 | NKX2-1 | NK2 homeobox 1 | 9.46E-08 | 1.58E-04 |
| 5599 | P45983 | MAPK8 | mitogen-activated protein kinase 8 | 1.11E-05 | 5.37E-03 |
| 5256 | P46019 | PHKA2 | phosphorylase kinase, alpha 2 (liver) | 2.46E-05 | 8.99E-03 |
| 6579 | P46721 | SLCO1A2 | solute carrier organic anion transporter family, member 1A2 | 4.90E-04 | 6.38E-02 |
| 5273 | P48595 | SERPINB10 | serpin peptidase inhibitor, clade B (ovalbumin), member 10 | 6.86E-05 | 1.74E-02 |
| 6878 | P49848 | TAF6 | TAF6 RNA polymerase II, TATA box binding protein (TBP)-associated factor, 80kDa | 2.95E-08 | 5.83E-05 |
| 5563 | P54646 | PRKAA2 | protein kinase, AMP-activated, alpha 2 catalytic subunit | 6.50E-04 | 7.56E-02 |
| 5700 | P62191 | PSMC1 | proteasome (prosome, macropain) 26S subunit, ATPase, 1 | 6.79E-04 | 7.77E-02 |
| 1142 | Q05901 | CHRNB3 | cholinergic receptor, nicotinic, beta 3 (neuronal) | 2.48E-10 | 1.08E-06 |
| 7185 | Q13077 | TRAF1 | TNF receptor-associated factor 1 | 2.37E-04 | 3.83E-02 |
| 5082 | Q13371 | PDCL | phosducin-like | 1.13E-04 | 2.34E-02 |
| 5164 | Q15119 | PDK2 | pyruvate dehydrogenase kinase, isozyme 2 | 8.37E-05 | 1.99E-02 |
| 6565 | Q16348 | SLC15A2 | solute carrier family 15 (H+/peptide transporter), member 2 | 1.07E-04 | 2.23E-02 |
| 2901 | Q16478 | GRIK5 | glutamate receptor, ionotropic, kainate 5 | 2.48E-06 | 1.74E-03 |
| 7360 | Q16851 | UGP2 | UDP-glucose pyrophosphorylase 2 | 2.48E-10 | 1.08E-06 |
| 29094 | Q3ZCW2 | LGALSL | lectin, galactoside-binding-like | 4.03E-04 | 5.59E-02 |
| 645974 | Q5JQF8 | PABPC1L2B | poly(A) binding protein, cytoplasmic 1-like 2B | 3.07E-05 | 1.02E-02 |
| 83479 | Q5T1V6 | DDX59 | DEAD (Asp-Glu-Ala-Asp) box polypeptide 59 | 9.04E-05 | 2.04E-02 |
| 343099 | Q5T9S5 | CCDC18 | coiled-coil domain containing 18 | 3.94E-04 | 5.59E-02 |
| 353142 | Q5TA76 | LCE3A | late cornified envelope 3A | 6.01E-06 | 3.44E-03 |
| 440561 | Q5VXH4 | PRAMEF6 | PRAME family member 6 | 1.58E-04 | 3.01E-02 |
| 387273 | Q6L8G5 | KRTAP5-10 | keratin associated protein 5-10 | 8.78E-04 | 9.27E-02 |
| 147719 | Q6UWN0 | LYPD4 | LY6/PLAUR domain containing 4 | 9.39E-04 | 9.76E-02 |
| 6830 | Q7KZ85 | SUPT6H | suppressor of Ty 6 homolog (S. cerevisiae) | 4.84E-04 | 6.34E-02 |
| 56971 | Q7Z692 | CEACAM19 | carcinoembryonic antigen-related cell adhesion molecule 19 | 1.91E-06 | 1.43E-03 |
| 346673 | Q7Z7C7 | STRA8 | stimulated by retinoic acid 8 | 3.01E-05 | 1.02E-02 |
| 9332 | Q86VB7 | CD163 | CD163 molecule | 1.74E-05 | 7.27E-03 |
| 340602 | Q86X51 | CXorf67 | chromosome X open reading frame 67 | 6.29E-05 | 1.63E-02 |
| 55258 | Q86YJ6 | THNSL2 | threonine synthase-like 2 (S. cerevisiae) | 4.90E-05 | 1.38E-02 |
| 222643 | Q8IV45 | UNC5CL | unc-5 homolog C (C. elegans)-like | 3.67E-05 | 1.15E-02 |
| 125336 | Q8IVV2 | LOXHD1 | lipoxygenase homology domains 1 | 1.77E-05 | 7.28E-03 |
| 196394 | Q8IY45 | AMN1 | antagonist of mitotic exit network 1 homolog (S. cerevisiae) | 1.04E-07 | 1.61E-04 |
| 165082 | Q8IZF5 | GPR113 | G protein-coupled receptor 113 | 7.80E-04 | 8.53E-02 |
| 139378 | Q8IZF6 | GPR112 | G protein-coupled receptor 112 | 3.71E-04 | 5.38E-02 |
| 84441 | Q8IZL2 | MAML2 | mastermind-like 2 (Drosophila) | 1.27E-04 | 2.53E-02 |
| 150696 | Q8N271 | PROM2 | prominin 2 | 1.27E-04 | 2.53E-02 |
| 26301 | Q8N5D6 | GBGT1 | globoside alpha-1,3-N-acetylgalactosaminyltransferase 1 | 2.72E-05 | 9.55E-03 |
| 84524 | Q8N5P1 | ZC3H8 | zinc finger CCCH-type containing 8 | 6.28E-05 | 1.63E-02 |
| 154075 | Q8N6K7 | SAMD3 | sterile alpha motif domain containing 3 | 2.48E-05 | 8.99E-03 |
| 100128374 | Q8N997 | LOC100128374 | uncharacterized LOC100128374 | 3.14E-05 | 1.02E-02 |
| 91133 | Q8NA19 | L3MBTL4 | l(3)mbt-like 4 (Drosophila) | 5.27E-04 | 6.51E-02 |
| 283899 | Q8NBZ0 | INO80E | INO80 complex subunit E | 3.21E-04 | 4.79E-02 |
| 128061 | Q8NDD1 | C1orf131 | chromosome 1 open reading frame 131 | 5.53E-05 | 1.52E-02 |
| 151112 | Q8NEG5 | ZSWIM2 | zinc finger, SWIM-type containing 2 | 6.20E-06 | 3.46E-03 |
| 9376 | Q8TCC7 | SLC22A8 | solute carrier family 22 (organic anion transporter), member 8 | 4.04E-04 | 5.59E-02 |
| 219970 | Q8WU03 | GLYATL2 | glycine-N-acyltransferase-like 2 | 3.43E-06 | 2.23E-03 |
| 54967 | Q8WUE5 | CXorf48 | chromosome X open reading frame 48 | 5.30E-04 | 6.51E-02 |
| 221143 | Q8WVE0 | N6AMT2 | N-6 adenine-specific DNA methyltransferase 2 (putative) | 2.33E-04 | 3.83E-02 |
| 114815 | Q8WY21 | SORCS1 | sortilin-related VPS10 domain containing receptor 1 | 5.44E-04 | 6.62E-02 |
| 89857 | Q8WZ60 | KLHL6 | kelch-like family member 6 | 1.35E-05 | 6.00E-03 |
| 84335 | Q96B36 | AKT1S1 | AKT1 substrate 1 (proline-rich) | 1.92E-05 | 7.54E-03 |
| 286075 | Q96C28 | ZNF707 | zinc finger protein 707 | 2.17E-04 | 3.63E-02 |
| 148156 | Q96NG5 | ZNF558 | zinc finger protein 558 | 2.61E-04 | 4.02E-02 |
| 114770 | Q96PD5 | PGLYRP2 | peptidoglycan recognition protein 2 | 4.81E-04 | 6.34E-02 |
| 319100 | Q96RI8 | TAAR6 | trace amine associated receptor 6 | 2.05E-04 | 3.49E-02 |
| 83852 | Q96T68 | SETDB2 | SET domain, bifurcated 2 | 3.49E-06 | 2.23E-03 |
| 8904 | Q99829 | CPNE1 | copine I | 1.13E-06 | 9.48E-04 |
| 51442 | Q99990 | VGLL1 | vestigial like 1 (Drosophila) | 3.20E-06 | 2.17E-03 |
| 11235 | Q9BUL8 | PDCD10 | programmed cell death 10 | 8.25E-04 | 8.88E-02 |
| 400684 | Q9BVU7 | LOC400684 | uncharacterized LOC400684 | 7.50E-06 | 3.98E-03 |
| 22950 | Q9BWU0 | SLC4A1AP | solute carrier family 4 (anion exchanger), member 1, adaptor protein | 5.02E-04 | 6.50E-02 |
| 81602 | Q9BWV3 | CDADC1 | cytidine and dCMP deaminase domain containing 1 | 1.15E-04 | 2.36E-02 |
| 114897 | Q9BXJ1 | C1QTNF1 | C1q and tumor necrosis factor related protein 1 | 1.09E-08 | 2.62E-05 |
| 83639 | Q9BY14 | TEX101 | testis expressed 101 | 4.47E-04 | 5.97E-02 |
| 83896 | Q9BYR8 | KRTAP3-1 | keratin associated protein 3-1 | 5.21E-04 | 6.51E-02 |
| 83955 | Q9BZK3 | NACAP1 | nascent-polypeptide-associated complex alpha polypeptide pseudogene 1 | 1.94E-05 | 7.54E-03 |
| 80313 | Q9C0I9 | LRRC27 | leucine rich repeat containing 27 | 7.41E-07 | 7.32E-04 |
| 64240 | Q9H222 | ABCG5 | ATP-binding cassette, sub-family G (WHITE), member 5 | 1.86E-04 | 3.29E-02 |
| 79144 | Q9H3Y8 | PPDPF | pancreatic progenitor cell differentiation and proliferation factor homolog (zebrafish) | 1.05E-04 | 2.22E-02 |
| 80264 | Q9H8G1 | ZNF430 | zinc finger protein 430 | 7.24E-04 | 8.07E-02 |
| 3166 | Q9NP08 | HMX1 | H6 family homeobox 1 | 6.90E-04 | 7.81E-02 |
| 5203 | Q9NQP4 | PFDN4 | prefoldin subunit 4 | 1.34E-04 | 2.60E-02 |
| 58491 | Q9NQZ8 | ZNF71 | zinc finger protein 71 | 5.93E-05 | 1.57E-02 |
| 56893 | Q9NRR5 | UBQLN4 | ubiquilin 4 | 4.03E-09 | 1.10E-05 |
| 55847 | Q9NZ45 | CISD1 | CDGSH iron sulfur domain 1 | 2.54E-04 | 3.97E-02 |
| 341416 | Q9NZP2 | OR6C2 | olfactory receptor, family 6, subfamily C, member 2 | 2.54E-04 | 3.97E-02 |
| 9465 | Q9P0M2 | AKAP7 | A kinase (PRKA) anchor protein 7 | 5.76E-04 | 6.89E-02 |
| 53407 | Q9P2W9 | STX18 | syntaxin 18 | 9.48E-07 | 8.39E-04 |
| 51192 | Q9UBR5 | CKLF | chemokine-like factor | 4.37E-04 | 5.90E-02 |
| 148223 | Q9UFG5 | C19orf25 | chromosome 19 open reading frame 25 | 4.75E-06 | 2.79E-03 |
| 9637 | Q9UHY8 | FEZ2 | fasciculation and elongation protein zeta 2 (zygin II) | 9.13E-04 | 9.55E-02 |
| 9783 | Q9UJD0 | RIMS3 | regulating synaptic membrane exocytosis 3 | 1.01E-04 | 2.15E-02 |
| 4733 | Q9Y295 | DRG1 | developmentally regulated GTP binding protein 1 | 1.64E-04 | 3.05E-02 |
| 51372 | Q9Y2S6 | TMA7 | translation machinery associated 7 homolog (S. cerevisiae) | 2.58E-05 | 9.18E-03 |
| 26985 | Q9Y2T2 | AP3M1 | adaptor-related protein complex 3, mu 1 subunit | 9.57E-04 | 9.87E-02 |
| 10809 | Q9Y365 | STARD10 | StAR-related lipid transfer (START) domain containing 10 | 7.56E-04 | 8.35E-02 |
| 730020 | - | LOC730020 | uncharacterized LOC730020 | 1.54E-13 | 1.68E-09 |
| 730081 | - | LOC730081 | uncharacterized LOC730081 | 1.41E-10 | 1.03E-06 |
| 284240 | - | LOC284240 | uncharacterized LOC284240 | 4.28E-08 | 7.76E-05 |
| 285441 | - | LOC285441 | uncharacterized LOC285441 | 3.73E-07 | 4.27E-04 |
| 100130456 | - | LOC100130456 | uncharacterized LOC100130456 | 4.33E-06 | 2.61E-03 |
| 641365 | - | LINC00616 | long intergenic non-protein coding RNA 616 | 1.98E-05 | 7.55E-03 |
| 728073 | - | LOC728073 | uncharacterized LOC728073 | 5.67E-05 | 1.54E-02 |
| 338799 | - | LOC338799 | uncharacterized LOC338799 | 9.25E-05 | 2.05E-02 |
| 100132832 | - | LOC100132832 | PMS2 postmeiotic segregation increased 2 (S. cerevisiae) pseudogene | 1.34E-04 | 2.60E-02 |
| 100288619 | - | LOC100288619 | uncharacterized LOC100288619 | 1.35E-04 | 2.60E-02 |
| 100131490 | - | LOC100131490 | uncharacterized LOC100131490 | 1.73E-04 | 3.16E-02 |
| 286189 | - | LOC286189 | uncharacterized LOC286189 | 1.86E-04 | 3.29E-02 |
| 100128881 | - | LOC100128881 | uncharacterized LOC100128881 | 2.33E-04 | 3.83E-02 |
| 387486 | - | LINC00320 | long intergenic non-protein coding RNA 320 | 3.82E-04 | 5.47E-02 |
| 100131432 | - | LOC100131432 | uncharacterized LOC100131432 | 3.96E-04 | 5.59E-02 |
| 100130301 | - | LOC100130301 | uncharacterized LOC100130301 | 5.76E-04 | 6.89E-02 |
| 100170226 | - | SNAR-C3 | small ILF3/NF90-associated RNA C3 | 6.73E-04 | 7.77E-02 |
| 677794 | - | SNORA2B | small nucleolar RNA, H/ACA box 2B | 8.67E-04 | 9.21E-02 |

**Table S8 Genes contained in the miRNA/mRNA network.** Network analysis revealed 164 mRNAs as targets of significantly altered miRNAs [11].

| Gene Name | Entrez Gene ID | Description | Constant expression during ageing |
| --- | --- | --- | --- |
| ANK1 | 286 | ankyrin 1, erythrocytic | No |
| ANKH | 56172 | ankylosis, progressive homolog (mouse) | Yes |
| APC | 324 | adenomatous polyposis coli | Yes |
| ARHGEF12 | 23365 | Rho guanine nucleotide exchange factor (GEF) 12 | Yes |
| ARL5A | 26225 | ADP-ribosylation factor-like 5A | Yes |
| ATP2B3 | 492 | ATPase, Ca++ transporting, plasma membrane 3 | No |
| ATP7A | 538 | ATPase, Cu++ transporting, alpha polypeptide | Yes |
| ATRX | 546 | alpha thalassemia/mental retardation syndrome X-linked | Yes |
| BAG5 | 9529 | BCL2-associated athanogene 5 | Yes |
| BCAT1 | 586 | branched chain amino-acid transaminase 1, cytosolic | No |
| BCL2L2 | 599 | BCL2-like 2 | Yes |
| BCL6B | 255877 | B-cell CLL/lymphoma 6, member B | No |
| BPTF | 2186 | bromodomain PHD nger transcription factor | No |
| BTAF1 | 9044 | BTAF1 RNA polymerase II, B-TFIID transcription factor-associated, 170kDa (Mot1 homolog, S. cerevisiae) | Yes |
| C11ORF61 | 79684 | Myb/SANT-like DNA-binding domain containing 2 | No |
| C15ORF29 | 79768 | chromosome 15 open reading frame 29 | No |
| C20ORF103 | 24141 | lysosomal-associated membrane protein family, member 5 | No |
| C5ORF13 | 9315 | neuronal regeneration related protein homolog (rat) | No |
| C5ORF24 | 134553 | chromosome 5 open reading frame 24 | Yes |
| CCNYL1 | 151195 | cyclin Y-like 1 | No |
| CDH13 | 1012 | cadherin 13, H-cadherin (heart) | No |
| CDS2 | 8760 | CDP-diacylglycerol synthase (phosphatidate cytidylyltransferase) 2 | Yes |
| CLDN12 | 9069 | claudin 12 | Yes |
| CNTN3 | 5067 | contactin 3 (plasmacytoma associated) | Yes |
| CORO2A | 7464 | coronin, actin binding protein, 2A | Yes |
| CP110 | 9738 | centriolar coiled coil protein 110kDa | No |
| CPEB4 | 80315 | cytoplasmic polyadenylation element binding protein 4 | Yes |
| CSF1 | 1435 | colony stimulating factor 1 (macrophage) | Yes |
| CTTNBP2NL | 55917 | CTTNBP2 N-terminal like | Yes |
| DEK | 7913 | DEK oncogene | No |
| EGLN2 | 112398 | egl nine homolog 2 (C. elegans) | Yes |
| EIF2C2 | 27161 | eukaryotic translation initiation factor 2C, 2 | Yes |
| ENPP1 | 5167 | ectonucleotide pyrophosphatase/phosphodiesterase 1 | Yes |
| EREG | 2069 | epiregulin | No |
| FAM122B | 159090 | family with sequence similarity 122B | Yes |
| FAM13B | 51306 | family with sequence similarity 13, member B | Yes |
| FAM160A2 | 84067 | family with sequence similarity 160, member A2 | Yes |
| FAM169A | 26049 | family with sequence similarity 169, member A | No |
| FBXO31 | 79791 | F-box protein 31 | Yes |
| FERMT2 | 10979 | fermitin family member 2 | Yes |
| FIBIN | 387758 | n bud initiation factor homolog (zebrash) | No |
| FLJ36031 | 168455 | coiled-coil domain containing 71-like | Yes |
| GABRB3 | 2562 | gamma-aminobutyric acid (GABA) A receptor, beta 3 | No |
| GK3P | 2710 | glycerol kinase | No |
| GLIS3 | 169792 | GLIS family zinc nger 3 | No |
| GOPC | 57120 | golgi-associated PDZ and coiled-coil motif containing | Yes |
| GPATCH8 | 23131 | G patch domain containing 8 | Yes |
| GRIK2 | 2898 | glutamate receptor, ionotropic, kainate 2 | No |
| GRIK2 | 2898 | glutamate receptor, ionotropic, kainate 2 | No |
| GUCY1A3 | 2982 | guanylate cyclase 1, soluble, alpha 3 | No |
| HSPA8 | 3312 | heat shock 70kDa protein 8 | Yes |
| INHBB | 3625 | inhibin, beta B | No |
| INO80 | 54617 | INO80 homolog (S. cerevisiae) | Yes |
| IRF1 | 3659 | interferon regulatory factor 1 | No |
| KDM5C | 8242 | lysine (K)-specic demethylase 5C | Yes |
| KIAA0319L | 79932 | KIAA0319-like | Yes |
| KIAA0776 | 23376 | UFM1-specic ligase 1 | Yes |
| KIAA1549 | 57670 | KIAA1549 | Yes |
| KIAA2022 | 340533 | KIAA2022 | No |
| KLF4 | 9314 | Kruppel-like factor 4 (gut) | Yes |
| LANCL2 | 55915 | LanC lantibiotic synthetase component C-like 2 (bacterial) | No |
| LIMS1 | 3987 | LIM and senescent cell antigen-like domains 1 | Yes |
| LMTK2 | 22853 | lemur tyrosine kinase 2 | Yes |
| LOC344593 | 5781 | protein tyrosine phosphatase, non-receptor type 11 | Yes |
| LOC645978 | 340485 | alkaline ceramidase 2 | No |
| LPHN2 | 23266 | latrophilin 2 | No |
| LRP1B | 53353 | low density lipoprotein receptor-related protein 1B | No |
| LRRC10B | 390205 | leucine rich repeat containing 10B | No |
| LRRC8D | 55144 | leucine rich repeat containing 8 family, member D | No |
| LRRFIP1 | 9208 | leucine rich repeat (in FLII) interacting protein 1 | Yes |
| LRRTM2 | 26045 | leucine rich repeat transmembrane neuronal 2 | No |
| MAP3K9 | 4293 | mitogen-activated protein kinase kinase kinase 9 | No |
| MAST3 | 23031 | microtubule associated serine/threonine kinase 3 | No |
| MBNL3 | 55796 | muscleblind-like splicing regulator 3 | Yes |
| MBTD1 | 54799 | mbt domain containing 1 | Yes |
| MCF2L | 23263 | MCF.2 cell line derived transforming sequence-like | Yes |
| MCL1 | 4170 | myeloid cell leukemia sequence 1 (BCL2-related) | Yes |
| MED15 | 51586 | mediator complex subunit 15 | Yes |
| METAP1 | 23173 | methionyl aminopeptidase 1 | Yes |
| MLEC | 9761 | malectin | Yes |
| MOSPD1 | 56180 | motile sperm domain containing 1 | Yes |
| MTOR | 2475 | mechanistic target of rapamycin (serine/threonine kinase) | Yes |
| NANOS1 | 340719 | nanos homolog 1 (Drosophila) | Yes |
| NCKIPSD | 51517 | NCK interacting protein with SH3 domain | Yes |
| NDRG2 | 57447 | NDRG family member 2 | No |
| NFIB | 4781 | nuclear factor I/B | No |
| NOS1AP | 9722 | nitric oxide synthase 1 (neuronal) adaptor protein | No |
| NOX4 | 50507 | NADPH oxidase 4 | No |
| NPTXR | 23467 | neuronal pentraxin receptor | Yes |
| NR1D2 | 9975 | nuclear receptor subfamily 1, group D, member 2 | Yes |
| NUDT21 | 11051 | nudix (nucleoside diphosphate linked moiety X)-type motif 21 | Yes |
| PANX2 | 56666 | pannexin 2 | No |
| PDE3B | 5140 | phosphodiesterase 3B, cGMP-inhibited | No |
| PDE5A | 8654 | phosphodiesterase 5A, cGMP-specic | No |
| PEX5L | 51555 | peroxisomal biogenesis factor 5-like | No |
| PI4K2B | 55300 | phosphatidylinositol 4-kinase type 2 beta | No |
| PIK3C2A | 5286 | phosphoinositide-3-kinase, class 2, alpha polypeptide | Yes |
| PIK3CD | 5293 | phosphoinositide-3-kinase, catalytic, delta polypeptide | No |
| PLA2G6 | 8398 | phospholipase A2, group VI (cytosolic, calcium-independent) | Yes |
| PNOC | 5368 | prepronociceptin | No |
| PRKCD | 5580 | protein kinase C, delta | Yes |
| PRKCSH | 5589 | protein kinase C substrate 80K-H | Yes |
| PSD3 | 23362 | pleckstrin and Sec7 domain containing 3 | Yes |
| PSMD7 | 5713 | proteasome (prosome, macropain) 26S subunit, non-ATPase, 7 | Yes |
| PTAR1 | 375743 | protein prenyltransferase alpha subunit repeat containing 1 | Yes |
| PTPRE | 5791 | protein tyrosine phosphatase, receptor type, E | No |
| RAB35 | 11021 | RAB35, member RAS oncogene family | Yes |
| RAB40B | 10966 | RAB40B, member RAS oncogene family | Yes |
| RAB5B | 5869 | RAB5B, member RAS oncogene family | Yes |
| RAD21 | 5885 | RAD21 homolog (S. pombe) | Yes |
| RALGAPA2 | 57186 | Ral GTPase activating protein, alpha subunit 2 (catalytic) | No |
| RBAK | 57786 | RB-associated KRAB zinc nger | No |
| RBPMS2 | 348093 | RNA binding protein with multiple splicing 2 | No |
| RET | 5979 | ret proto-oncogene | No |
| RPS6KA1 | 6195 | ribosomal protein S6 kinase, 90kDa, polypeptide 1 | No |
| RTF1 | 23168 | Rtf1, Paf1/RNA polymerase II complex component, homolog (S. cerevisiae) | Yes |
| SACM1L | 22908 | SAC1 suppressor of actin mutations 1-like (yeast) | Yes |
| SCN2B | 6327 | sodium channel, voltage-gated, type II, beta subunit | No |
| SCN9A | 6335 | sodium channel, voltage-gated, type IX, alpha subunit | No |
| SEMA6A | 57556 | sema domain, transmembrane domain (TM), and cytoplasmic domain, (semaphorin) 6A | No |
| SERTAD2 | 9792 | SERTA domain containing 2 | Yes |
| SHOC2 | 8036 | soc-2 suppressor of clear homolog (C. elegans) | Yes |
| SIX4 | 51804 | SIX homeobox 4 | No |
| SKP1 | 6500 | S-phase kinase-associated protein 1 | Yes |
| SLC10A7 | 84068 | solute carrier family 10 (sodium/bile acid cotransporter family), member 7 | Yes |
| SLC16A6 | 9120 | solute carrier family 16, member 6 (monocarboxylic acid transporter 7) | No |
| SLC19A2 | 10560 | solute carrier family 19 (thiamine transporter), member 2 | Yes |
| SLC26A3 | 1811 | solute carrier family 26, member 3 | No |
| SLC38A2 | 54407 | solute carrier family 38, member 2 | Yes |
| SMOC2 | 64094 | SPARC related modular calcium binding 2 | No |
| SNAP25 | 6616 | synaptosomal-associated protein, 25kDa | No |
| SOCS4 | 122809 | suppressor of cytokine signaling 4 | Yes |
| SPG20 | 23111 | spastic paraplegia 20 (Troyer syndrome) | Yes |
| SSX2IP | 117178 | synovial sarcoma, X breakpoint 2 interacting protein | Yes |
| STRN | 6801 | striatin, calmodulin binding protein | No |
| TAC1 | 6863 | tachykinin, precursor 1 | No |
| TACC2 | 10579 | transforming, acidic coiled-coil containing protein 2 | Yes |
| TBPL1 | 9519 | TBP-like 1 | Yes |
| TCF20 | 6942 | transcription factor 20 (AR1) | Yes |
| TMBIM6 | 7009 | transmembrane BAX inhibitor motif containing 6 | Yes |
| TMEM151B | 441151 | transmembrane protein 151B | No |
| TMEM168 | 64418 | transmembrane protein 168 | Yes |
| TMEM71 | 137835 | transmembrane protein 71 | Yes |
| TNFAIP3 | 7128 | tumor necrosis factor, alpha-induced protein 3 | No |
| TNFRSF21 | 27242 | tumor necrosis factor receptor superfamily, member 21 | Yes |
| TNRC6B | 23112 | trinucleotide repeat containing 6B | Yes |
| TNS1 | 7145 | tensin 1 | Yes |
| TPRG1L | 127262 | tumor protein p63 regulated 1-like | Yes |
| TRAF3 | 7187 | TNF receptor-associated factor 3 | Yes |
| TRPM7 | 54822 | transient receptor potential cation channel, subfamily M, member 7 | No |
| TRPS1 | 7227 | trichorhinophalangeal syndrome I | Yes |
| TSPYL2 | 64061 | TSPY-like 2 | No |
| UBE2B | 7320 | ubiquitin-conjugating enzyme E2B | Yes |
| UQCC | 55245 | ubiquinol-cytochrome c reductase complex chaperone | Yes |
| USP46 | 64854 | ubiquitin specic peptidase 46 | Yes |
| WEE1 | 7465 | WEE1 homolog (S. pombe) | No |
| WNK1 | 65125 | WNK lysine decient protein kinase 1 | Yes |
| WSB1 | 26118 | WD repeat and SOCS box containing 1 | Yes |
| ZBTB39 | 9880 | zinc nger and BTB domain containing 39 | No |
| ZBTB4 | 57659 | zinc nger and BTB domain containing 4 | Yes |
| ZBTB43 | 23099 | zinc nger and BTB domain containing 43 | Yes |
| ZC3H7B | 23264 | zinc nger CCCH-type containing 7B | Yes |
| ZNF711 | 7552 | zinc nger protein 711 | No |
| ZNF83 | 55769 | zinc nger protein 83 | Yes |

**Table S9 Biological processes significantly altered with age published by Kalfalah *et al*. [10].** Statistical analysis of transcriptome data revealed 117 enriched biological processes altered with age.

| Gene Set | geometric mean of p-values | Mean statistics | p-value | q-value | Gene set size | Regulation |
| --- | --- | --- | --- | --- | --- | --- |
| Cell Cycle | 1.36E-06 | 1.46E-01 | 2.22E-11 | 4.21E-08 | 374 | down |
| Amyloids | 2.92E-05 | 2.83E-01 | 1.14E-10 | 1.08E-07 | 60 | down |
| Meiotic Recombination | 3.74E-05 | 2.82E-01 | 5.47E-10 | 3.45E-07 | 60 | down |
| RNA Polymerase I Promoter Opening | 5.28E-05 | 2.74E-01 | 1.08E-09 | 5.14E-07 | 61 | down |
| Chromosome Maintenance | 1.44E-04 | 2.08E-01 | 1.25E-08 | 4.73E-06 | 111 | down |
| Oxidative phosphorylation - Homo sapiens (human) | 2.17E-03 | 1.76E-01 | 1.77E-08 | 5.04E-06 | 117 | down |
| Electron Transport Chain | 1.47E-03 | 2.15E-01 | 1.86E-08 | 5.04E-06 | 90 | down |
| The citric acid (TCA) cycle and respiratory electron transport | 1.84E-03 | 1.79E-01 | 2.90E-08 | 6.87E-06 | 117 | down |
| Oxidative phosphorylation | 4.07E-03 | 2.37E-01 | 4.24E-08 | 8.92E-06 | 52 | down |
| Packaging Of Telomere Ends | 1.79E-04 | 2.82E-01 | 5.65E-08 | 9.80E-06 | 51 | down |
| Meiosis | 2.59E-04 | 2.36E-01 | 5.69E-08 | 9.80E-06 | 73 | down |
| Translation | 2.43E-03 | 1.61E-01 | 1.31E-07 | 2.07E-05 | 145 | down |
| Respiratory electron transport | 2.77E-03 | 2.36E-01 | 2.12E-07 | 3.09E-05 | 65 | down |
| Cell Cycle, Mitotic | 1.45E-05 | 1.63E-01 | 2.89E-07 | 3.92E-05 | 289 | down |
| 3, -UTR-mediated translational regulation | 6.35E-03 | 1.78E-01 | 2.12E-06 | 2.51E-04 | 104 | down |
| L13a-mediated translational silencing of Ceruloplasmin expression | 6.35E-03 | 1.78E-01 | 2.12E-06 | 2.51E-04 | 104 | down |
| Formation of a pool of free 40S subunits | 6.11E-03 | 1.85E-01 | 2.72E-06 | 2.95E-04 | 94 | down |
| Mitotic M-M/G1 phases | 1.67E-05 | 2.21E-01 | 2.81E-06 | 2.95E-04 | 145 | down |
| Alzheimer,s disease - Homo sapiens (human) | 8.57E-03 | 1.25E-01 | 4.08E-06 | 4.07E-04 | 160 | down |
| GTP hydrolysis and joining of the 60S ribosomal subunit | 8.31E-03 | 1.72E-01 | 4.73E-06 | 4.48E-04 | 104 | down |
| Cap-dependent Translation Initiation | 8.32E-03 | 1.73E-01 | 6.09E-06 | 5.50E-04 | 103 | down |
| Parkinson,s disease - Homo sapiens (human) | 9.48E-03 | 1.68E-01 | 7.28E-06 | 6.27E-04 | 116 | down |
| Nonsense Mediated Decay Independent of the Exon Junction Complex | 8.70E-03 | 1.91E-01 | 8.27E-06 | 6.81E-04 | 90 | down |
| Huntington,s disease - Homo sapiens (human) | 1.48E-02 | 1.21E-01 | 8.63E-06 | 6.82E-04 | 176 | down |
| Beta1 integrin cell surface interactions | 6.40E-03 | 1.95E-01 | 9.05E-06 | 6.86E-04 | 66 | down |
| Metabolism of proteins | 4.91E-03 | 8.37E-02 | 9.53E-06 | 6.94E-04 | 454 | down |
| Meiotic Synapsis | 2.70E-03 | 2.58E-01 | 1.02E-05 | 7.02E-04 | 45 | down |
| SRP-dependent cotranslational protein targeting to membrane | 6.01E-03 | 1.82E-01 | 1.04E-05 | 7.02E-04 | 108 | down |
| Peptide chain elongation | 8.99E-03 | 1.95E-01 | 1.19E-05 | 7.53E-04 | 85 | down |
| RNA Polymerase I Chain Elongation | 1.49E-03 | 2.10E-01 | 1.21E-05 | 7.53E-04 | 77 | down |
| ECM-receptor interaction - Homo sapiens (human) | 1.41E-02 | 1.58E-01 | 1.23E-05 | 7.53E-04 | 83 | down |
| Cytoplasmic Ribosomal Proteins | 9.82E-03 | 1.85E-01 | 1.42E-05 | 8.38E-04 | 88 | down |
| Olfactory transduction - Homo sapiens (human) | 4.95E-03 | 7.83E-02 | 1.96E-05 | 1.12E-03 | 388 | down |
| Viral mRNA Translation | 9.93E-03 | 1.97E-01 | 2.02E-05 | 1.12E-03 | 83 | down |
| Deposition of New CENPA-containing Nucleosomes at the Centromere | 1.09E-03 | 3.19E-01 | 2.28E-05 | 1.20E-03 | 35 | down |
| Nucleosome assembly | 1.09E-03 | 3.19E-01 | 2.28E-05 | 1.20E-03 | 35 | down |
| Ribosome - Homo sapiens (human) | 1.29E-02 | 1.95E-01 | 3.50E-05 | 1.79E-03 | 88 | down |
| Focal Adhesion | 1.47E-02 | 1.08E-01 | 6.83E-05 | 3.40E-03 | 184 | down |
| Systemic lupus erythematosus - Homo sapiens (human) | 4.53E-03 | 1.51E-01 | 7.70E-05 | 3.74E-03 | 130 | down |
| Hemostasis | 2.40E-02 | 6.30E-02 | 8.53E-05 | 3.96E-03 | 458 | down |
| RNA Polymerase I Transcription | 3.60E-03 | 1.89E-01 | 8.57E-05 | 3.96E-03 | 86 | down |
| Focal adhesion - Homo sapiens (human) | 1.89E-02 | 1.06E-01 | 8.87E-05 | 4.00E-03 | 201 | down |
| Axon guidance | 2.29E-02 | 8.32E-02 | 1.74E-04 | 7.27E-03 | 262 | down |
| stathmin and breast cancer resistance to antimicrotubule agents | 2.57E-02 | 2.69E-01 | 1.76E-04 | 7.27E-03 | 24 | down |
| Purine metabolism | 2.63E-02 | 9.00E-02 | 2.05E-04 | 8.28E-03 | 218 | down |
| Regulation of activated PAK-2p34 by proteasome mediated degradation | 2.83E-02 | 2.08E-01 | 2.27E-04 | 8.96E-03 | 45 | down |
| Extracellular matrix organization | 3.70E-02 | 1.32E-01 | 3.13E-04 | 1.20E-02 | 93 | down |
| Unwinding of DNA | 3.30E-03 | 5.50E-01 | 3.17E-04 | 1.20E-02 | 11 | down |
| Aurora B signaling | 3.14E-03 | 2.92E-01 | 3.35E-04 | 1.23E-02 | 41 | down |
| Integrin | 2.68E-02 | 1.26E-01 | 3.38E-04 | 1.23E-02 | 124 | down |
| COPI Mediated Transport | 3.24E-02 | 4.06E-01 | 3.66E-04 | 1.28E-02 | 10 | down |
| Golgi to ER Retrograde Transport | 3.24E-02 | 4.06E-01 | 3.66E-04 | 1.28E-02 | 10 | down |
| DNA strand elongation | 1.64E-03 | 3.53E-01 | 4.30E-04 | 1.48E-02 | 31 | down |
| mcalpain and friends in cell motility | 4.04E-02 | 2.20E-01 | 4.46E-04 | 1.49E-02 | 30 | down |
| Proteasome - Homo sapiens (human) | 3.87E-02 | 2.12E-01 | 4.48E-04 | 1.49E-02 | 44 | down |
| Alcoholism - Homo sapiens (human) | 1.38E-02 | 1.09E-01 | 4.59E-04 | 1.50E-02 | 176 | down |
| Axon guidance - Homo sapiens (human) | 3.56E-02 | 1.13E-01 | 6.28E-04 | 2.02E-02 | 129 | down |
| M Phase | 4.79E-04 | 2.19E-01 | 9.37E-04 | 2.93E-02 | 110 | down |
| Formation of ATP by chemiosmotic coupling | 4.92E-02 | 3.49E-01 | 9.55E-04 | 2.93E-02 | 13 | down |
| Lymphocyte TarBase | 1.24E-02 | 7.40E-02 | 9.59E-04 | 2.93E-02 | 418 | down |
| AKT phosphorylates targets in the cytosol | 4.56E-02 | 3.32E-01 | 1.01E-03 | 3.03E-02 | 13 | down |
| Synthesis of DNA | 4.16E-03 | 5.05E-01 | 1.02E-03 | 3.03E-02 | 13 | down |
| Collagen biosynthesis and modifying enzymes | 4.66E-02 | 1.50E-01 | 1.10E-03 | 3.15E-02 | 65 | down |
| Collagen formation | 4.66E-02 | 1.50E-01 | 1.10E-03 | 3.15E-02 | 65 | down |
| Epithelium TarBase | 1.51E-02 | 8.60E-02 | 1.18E-03 | 3.33E-02 | 278 | down |
| G1/S Transition | 3.75E-03 | 2.47E-01 | 1.35E-03 | 3.71E-02 | 60 | down |
| FOXM1 transcription factor network | 2.12E-02 | 2.29E-01 | 1.35E-03 | 3.71E-02 | 42 | down |
| DNA replication - Homo sapiens (human) | 5.03E-03 | 3.00E-01 | 1.38E-03 | 3.72E-02 | 36 | down |
| phosphorylation of mek1 by cdk5/p35 down regulates the map kinase pathway | 5.76E-02 | 3.05E-01 | 1.51E-03 | 4.04E-02 | 14 | down |
| Degradation of beta-catenin by the destruction complex | 5.31E-02 | 1.78E-01 | 1.55E-03 | 4.08E-02 | 51 | down |
| Proteasome Degradation | 5.42E-02 | 1.51E-01 | 1.61E-03 | 4.18E-02 | 65 | down |
| DNA Replication Pre-Initiation | 4.31E-03 | 3.06E-01 | 1.71E-03 | 4.31E-02 | 35 | down |
| M/G1 Transition | 4.31E-03 | 3.06E-01 | 1.71E-03 | 4.31E-02 | 35 | down |
| Cell-extracellular matrix interactions | 6.60E-02 | 2.77E-01 | 1.83E-03 | 4.56E-02 | 16 | down |
| Melanoma - Homo sapiens (human) | 7.07E-02 | 1.31E-01 | 1.87E-03 | 4.60E-02 | 71 | down |
| S Phase | 3.51E-03 | 2.37E-01 | 1.90E-03 | 4.61E-02 | 66 | down |
| Transcription | 2.87E-02 | 9.80E-02 | 1.96E-03 | 4.69E-02 | 212 | down |
| Amino acid synthesis and interconversion (transamination) | 5.52E-02 | 2.96E-01 | 2.06E-03 | 4.89E-02 | 16 | down |
| Integrin-mediated cell adhesion | 4.74E-02 | 1.18E-01 | 2.12E-03 | 4.97E-02 | 99 | down |
| Glucose metabolism | 7.17E-02 | 1.38E-01 | 2.16E-03 | 4.98E-02 | 64 | down |
| Tryptophan Metabolism | 4.66E-02 | 2.81E-01 | 2.46E-03 | 5.61E-02 | 17 | down |
| Smooth Muscle Contraction | 6.07E-02 | 2.33E-01 | 2.73E-03 | 6.15E-02 | 24 | down |
| Cell Cycle Checkpoints | 7.03E-02 | 2.87E-01 | 2.79E-03 | 6.21E-02 | 16 | down |
| colanic acid building blocks biosynthesis | 7.55E-02 | 2.97E-01 | 2.83E-03 | 6.23E-02 | 13 | down |
| Rho GTPase cycle | 7.11E-02 | 1.05E-01 | 2.97E-03 | 6.39E-02 | 126 | down |
| Signaling by Rho GTPases | 7.11E-02 | 1.05E-01 | 2.97E-03 | 6.39E-02 | 126 | down |
| Mitotic G1-G1/S phases | 6.63E-03 | 1.96E-01 | 3.01E-03 | 6.41E-02 | 84 | down |
| Arrhythmogenic right ventricular cardiomyopathy | 7.76E-02 | 1.34E-01 | 3.50E-03 | 7.38E-02 | 74 | down |
| Regulation of Actin Cytoskeleton | 5.12E-02 | 9.88E-02 | 3.79E-03 | 7.88E-02 | 146 | down |
| Validated transcriptional targets of deltaNp63 isoforms | 8.21E-02 | 1.55E-01 | 3.95E-03 | 8.13E-02 | 47 | down |
| proteasome complex | 5.66E-02 | 2.59E-01 | 4.19E-03 | 8.54E-02 | 24 | down |
| Translation initiation complex formation | 5.05E-02 | 1.97E-01 | 4.28E-03 | 8.63E-02 | 54 | down |
| DNA Replication | 4.71E-03 | 2.80E-01 | 4.33E-03 | 8.63E-02 | 42 | down |
| Signal transduction by L1 | 6.94E-02 | 2.28E-01 | 4.42E-03 | 8.65E-02 | 22 | down |
| Developmental Biology | 3.55E-02 | 7.20E-02 | 4.43E-03 | 8.65E-02 | 355 | down |
| TGF Beta Signaling Pathway | 9.07E-02 | 1.49E-01 | 4.83E-03 | 9.35E-02 | 54 | down |
| PLK1 signaling events | 3.76E-03 | 2.93E-01 | 5.01E-03 | 9.59E-02 | 44 | down |
| Metabolism of mRNA | 8.89E-03 | 1.25E-01 | 5.36E-07 | 1.01E-03 | 170 | up |
| superpathway of cholesterol biosynthesis | 3.17E-03 | 3.53E-01 | 2.48E-06 | 1.95E-03 | 25 | up |
| Nonsense Mediated Decay Enhanced by the Exon Junction Complex | 1.10E-02 | 1.77E-01 | 4.11E-06 | 1.95E-03 | 101 | up |
| Nonsense-Mediated Decay | 1.10E-02 | 1.77E-01 | 4.11E-06 | 1.95E-03 | 101 | up |
| Steroid Biosynthesis | 5.46E-03 | 3.70E-01 | 6.78E-06 | 2.57E-03 | 21 | up |
| Cholesterol Biosynthesis | 1.09E-02 | 3.93E-01 | 1.17E-04 | 2.03E-02 | 17 | up |
| Rheumatoid arthritis - Homo sapiens (human) | 2.66E-02 | 1.44E-01 | 1.18E-04 | 2.03E-02 | 89 | up |
| Intestinal immune network for IgA production - Homo sapiens (human) | 2.82E-02 | 1.95E-01 | 1.93E-04 | 2.19E-02 | 48 | up |
| Type I diabetes mellitus - Homo sapiens (human) | 2.29E-02 | 2.04E-01 | 2.03E-04 | 2.19E-02 | 43 | up |
| Antigen processing and presentation - Homo sapiens (human) | 2.42E-02 | 1.62E-01 | 2.96E-04 | 2.80E-02 | 71 | up |
| Graft-versus-host disease - Homo sapiens (human) | 1.98E-02 | 2.22E-01 | 3.15E-04 | 2.84E-02 | 39 | up |
| Zinc transporters | 5.94E-02 | 2.80E-01 | 9.47E-04 | 6.97E-02 | 17 | up |
| Spliceosome - Homo sapiens (human) | 2.10E-02 | 1.41E-01 | 9.56E-04 | 6.97E-02 | 127 | up |
| superpathway of geranylgeranyldiphosphate biosynthesis I (via mevalonate) | 3.25E-02 | 3.94E-01 | 1.09E-03 | 7.67E-02 | 12 | up |
| mRNA surveillance pathway - Homo sapiens (human) | 5.41E-02 | 1.33E-01 | 1.18E-03 | 7.79E-02 | 89 | up |
| Staphylococcus aureus infection - Homo sapiens (human) | 3.87E-02 | 1.82E-01 | 1.22E-03 | 7.79E-02 | 52 | up |
| Activation of NF-kappaB in B Cells | 6.02E-02 | 2.73E-01 | 1.25E-03 | 7.79E-02 | 18 | up |
| Allograft rejection - Homo sapiens (human) | 3.28E-02 | 2.16E-01 | 1.27E-03 | 7.79E-02 | 37 | up |
| cholesterol biosynthesis I | 2.08E-02 | 4.00E-01 | 1.45E-03 | 8.57E-02 | 13 | up |
| Cholesterol biosynthesis | 3.12E-02 | 3.97E-01 | 1.55E-03 | 8.92E-02 | 12 | up |

**Table S10 Enriched biological processes of proteins found with an age-associated alteration.** To uncover biological processes linked to age-associated proteins in the proteome of *in situ* aged fibroblasts, we performed a detailed network and enrichment analysis using Gene Ontology biological processes. For the 43 age-associated altered proteins, we assigned 71 unique biological processes as highly enriched (p ≤ 0.01). Correction for multiple testing was applied using Benjamini-Hochberg method.

| GO ID | GO Term | Nr. Genes | % Associated Genes | p-value | Corrected p-value | Associated Genes Found |
| --- | --- | --- | --- | --- | --- | --- |
| GO:0007507 | heart development | 3 | 4.8 | 3.5E-03 | 7.8E-03 | [BASP1, GARS, THBS1] |
| GO:0030154 | cell differentiation | 14 | 4.0 | 9.5E-57 | 1.8E-55 | [AP2A1, AP2A2, ARHGEF2, BASP1, CAP1, DHX15, EHD2, GARS, HSPA1A, HSPA2, MYL6, REXO2, RTCB, TWF2] |
| GO:0030855 | epithelial cell differentiation | 4 | 5.7 | 6.5E-04 | 1.6E-03 | [BASP1, DHX15, HSPA2, REXO2] |
| GO:0045595 | regulation of cell differentiation | 5 | 3.5 | 1.9E-15 | 1.1E-14 | [ARHGEF2, EHD2, GARS, HSPA1A, TWF2] |
| GO:0045597 | positive regulation of cell differentiation | 3 | 4.8 | 1.5E-04 | 4.2E-04 | [GARS, HSPA1A, TWF2] |
| GO:0048468 | cell development | 11 | 4.6 | 1.9E-56 | 3.2E-55 | [AP2A1, AP2A2, ARHGEF2, BASP1, CAP1, DHX15, GARS, HSPA2, MYL6, RTCB, TWF2] |
| GO:0048667 | cell morphogenesis involved in neuron differentiation | 6 | 6.1 | 2.9E-03 | 6.5E-03 | [AP2A1, AP2A2, CAP1, GARS, MYL6, TWF2] |
| GO:0048699 | generation of neurons | 7 | 4.1 | 1.9E-13 | 1.0E-12 | [AP2A1, AP2A2, ARHGEF2, CAP1, GARS, MYL6, TWF2] |
| GO:0050767 | regulation of neurogenesis | 3 | 4.9 | 2.2E-03 | 5.1E-03 | [ARHGEF2, GARS, TWF2] |
| GO:2000026 | regulation of multicellular organismal development | 6 | 4.2 | 4.4E-16 | 2.7E-15 | [ARHGEF2, BASP1, GARS, HSPA1A, THBS1, TWF2] |
| GO:0051094 | positive regulation of developmental process | 6 | 6.4 | 2.3E-05 | 7.4E-05 | [BASP1, EHD2, GARS, HSPA1A, THBS1, TWF2] |
| GO:0000902 | cell morphogenesis | 10 | 6.2 | 6.8E-07 | 2.8E-06 | [AP2A1, AP2A2, ARHGEF2, BASP1, CAP1, DHX15, GARS, MYL6, RTCB, TWF2] |
| GO:0033043 | regulation of organelle organization | 4 | 3.4 | 1.1E-04 | 3.3E-04 | [ARHGEF2, NMT1, REXO2, TWF2] |
| GO:0034622 | cellular macromolecular complex assembly | 6 | 3.8 | 1.7E-03 | 4.0E-03 | [DDX1, DDX3X, RPS6, SRSF9, TCP1, TWF2] |
| GO:0048812 | neuron projection morphogenesis | 6 | 5.9 | 2.7E-03 | 6.2E-03 | [AP2A1, AP2A2, CAP1, GARS, MYL6, TWF2] |
| GO:0051130 | positive regulation of cellular component organization | 5 | 4.4 | 8.0E-04 | 2.0E-03 | [EHD2, GARS, NMT1, REXO2, TWF2] |
| GO:0065003 | macromolecular complex assembly | 9 | 3.3 | 5.4E-11 | 2.7E-10 | [DDX1, DDX3X, EHD2, RPS6, SHMT2, SRSF9, STOM, TCP1, TWF2] |
| GO:0071822 | protein complex subunit organization | 10 | 3.0 | 3.2E-15 | 1.9E-14 | [ARHGEF2, DDX3X, EHD2, RPS29, RPS5, RPS6, SHMT2, STOM, TCP1, TWF2] |
| GO:0022603 | regulation of anatomical structure morphogenesis | 5 | 4.9 | 1.4E-04 | 4.1E-04 | [BASP1, EHD2, GARS, THBS1, TWF2] |
| GO:0034613 | cellular protein localization | 10 | 3.3 | 1.3E-07 | 6.1E-07 | [AP2A1, AP2A2, ARHGEF2, CLTB, EHD2, NMT1, REXO2, RPS29, RPS5, RPS6] |
| GO:0006468 | protein phosphorylation | 6 | 4.1 | 3.7E-14 | 2.0E-13 | [ARHGEF2, DNAJC3, GARS, HSPA2, REXO2, THBS1] |
| GO:0031399 | regulation of protein modification process | 7 | 3.8 | 1.4E-11 | 7.2E-11 | [ARHGEF2, DNAJC3, HSPA2, PSMD1, PSMD14, REXO2, THBS1] |
| GO:0032270 | positive regulation of cellular protein metabolic process | 7 | 4.4 | 1.9E-06 | 7.0E-06 | [ARHGEF2, DDX3X, DNAJC3, HSPA2, PSMD1, PSMD14, THBS1] |
| GO:0044267 | cellular protein metabolic process | 18 | 3.0 | 4.3E-49 | 4.7E-48 | [ARHGEF2, CCT6A, DDX1, DDX3X, DNAJC3, GARS, HSPA1A, HSPA2, NMT1, PSMD1, PSMD14, QARS, REXO2, RPS29, RPS5, RPS6, TCP1, THBS1] |
| GO:0051246 | regulation of protein metabolic process | 11 | 3.8 | 4.1E-43 | 4.0E-42 | [ARHGEF2, CAP1, DDX1, DDX3X, DNAJC3, HSPA2, PSMD1, PSMD14, REXO2, RPS5, THBS1] |
| GO:0010605 | negative regulation of macromolecule metabolic process | 8 | 3.6 | 8.7E-30 | 7.5E-29 | [BASP1, DDX3X, DNAJC3, PSMD1, PSMD14, REXO2, SRSF9, THBS1] |
| GO:0019220 | regulation of phosphate metabolic process | 6 | 3.3 | 8.7E-26 | 6.8E-25 | [ARHGEF2, CAP1, DNAJC3, HSPA2, REXO2, THBS1] |
| GO:0031325 | positive regulation of cellular metabolic process | 10 | 3.4 | 5.2E-62 | 1.6E-60 | [ARHGEF2, C14orf166, CAP1, DDX3X, DNAJC3, GARS, HSPA2, PSMD1, PSMD14, THBS1] |
| GO:0031328 | positive regulation of cellular biosynthetic process | 6 | 3.9 | 4.8E-22 | 3.5E-21 | [ARHGEF2, C14orf166, CAP1, DDX3X, GARS, THBS1] |
| GO:0043085 | positive regulation of catalytic activity | 7 | 4.1 | 4.8E-08 | 2.3E-07 | [CAP1, DDX3X, DNAJC3, HSPA2, PSMD1, PSMD14, THBS1] |
| GO:0044265 | cellular macromolecule catabolic process | 6 | 3.1 | 2.0E-05 | 6.5E-05 | [HSPA1A, PSMD1, PSMD14, RPS29, RPS5, RPS6] |
| GO:0050790 | regulation of catalytic activity | 10 | 3.9 | 1.4E-29 | 1.2E-28 | [CAP1, DDX3X, DNAJC3, HSPA2, NQO1, PPP1R7, PSMD1, PSMD14, REXO2, THBS1] |
| GO:0051171 | regulation of nitrogen compound metabolic process | 12 | 3.1 | 2.8E-64 | 1.4E-62 | [ARHGEF2, BASP1, C14orf166, CAP1, DDX1, DDX3X, DNAJC3, GARS, NQO1, PSMD1, PSMD14, SRSF9] |
| GO:0051336 | regulation of hydrolase activity | 5 | 3.4 | 6.0E-07 | 2.5E-06 | [DDX3X, DNAJC3, PSMD14, REXO2, THBS1] |
| GO:0051345 | positive regulation of hydrolase activity | 3 | 3.2 | 4.9E-04 | 1.3E-03 | [DDX3X, DNAJC3, PSMD14] |
| GO:1901566 | organonitrogen compound biosynthetic process | 3 | 3.0 | 1.1E-06 | 4.3E-06 | [CAP1, NQO1, SHMT2] |
| GO:0006366 | transcription from RNA polymerase II promoter | 5 | 3.1 | 9.0E-54 | 1.4E-52 | [ARHGEF2, C14orf166, DDX3X, GARS, SRSF9] |
| GO:0006396 | RNA processing | 5 | 3.0 | 3.9E-05 | 1.2E-04 | [DDX1, DHX15, RPS6, RTCB, SRSF9] |
| GO:0016070 | RNA metabolic process | 17 | 3.3 | 3.6E-53 | 5.1E-52 | [ARHGEF2, BASP1, C14orf166, CAP1, DDX1, DDX3X, DHX15, GARS, HSPA1A, PSMD1, PSMD14, QARS, RPS29, RPS5, RPS6, RTCB, SRSF9] |
| GO:0032774 | RNA biosynthetic process | 11 | 3.1 | 5.8E-66 | 8.9E-64 | [ARHGEF2, BASP1, C14orf166, CAP1, DDX1, DDX3X, GARS, RPS29, RPS5, RPS6, SRSF9] |
| GO:0045935 | positive regulation of nucleobase-containing compound metabolic process | 5 | 3.8 | 2.8E-19 | 2.0E-18 | [ARHGEF2, C14orf166, CAP1, DDX3X, GARS] |
| GO:0000165 | MAPK cascade | 2 | 3.2 | 1.4E-05 | 4.8E-05 | [GARS, THBS1] |
| GO:0007165 | signal transduction | 18 | 3.2 | 8.3E-52 | 9.9E-51 | [ANXA4, ANXA5, AP2A1, AP2A2, ARHGEF2, CAP1, DDX3X, DHX15, DNAJC3, GARS, HSPA1A, IGF2R, NMT1, PSMD1, PSMD14, REXO2, RPS6, THBS1] |
| GO:0007166 | cell surface receptor signaling pathway | 12 | 4.3 | 3.4E-60 | 8.8E-59 | [AP2A1, AP2A2, ARHGEF2, CAP1, DDX3X, DHX15, GARS, HSPA1A, IGF2R, NMT1, RPS6, THBS1] |
| GO:0007167 | enzyme linked receptor protein signaling pathway | 7 | 5.6 | 1.9E-07 | 8.1E-07 | [AP2A1, AP2A2, ARHGEF2, GARS, IGF2R, RPS6, THBS1] |
| GO:0007186 | G-protein coupled receptor signaling pathway | 2 | 6.3 | 7.5E-07 | 3.0E-06 | [DHX15, NMT1] |
| GO:0007268 | synaptic transmission | 4 | 6.3 | 7.7E-06 | 2.7E-05 | [AP2A1, AP2A2, DHX15, NQO1] |
| GO:0009968 | negative regulation of signal transduction | 6 | 5.6 | 1.2E-05 | 4.0E-05 | [AP2A1, AP2A2, ARHGEF2, DDX3X, HSPA1A, THBS1] |
| GO:0010646 | regulation of cell communication | 9 | 3.2 | 2.8E-65 | 2.2E-63 | [AP2A1, AP2A2, ARHGEF2, DDX3X, DHX15, HSPA1A, NMT1, REXO2, THBS1] |
| GO:0035556 | intracellular signal transduction | 9 | 3.0 | 3.1E-64 | 1.2E-62 | [ARHGEF2, DDX3X, GARS, NMT1, PSMD1, PSMD14, REXO2, RPS6, THBS1] |
| GO:0042981 | regulation of apoptotic process | 13 | 6.2 | 1.5E-07 | 6.8E-07 | [ANXA4, ANXA5, ARHGEF2, CAP1, DDX3X, HSPA1A, NMT1, NQO1, PSMD1, PSMD14, REXO2, RPS6, THBS1] |
| GO:0043066 | negative regulation of apoptotic process | 7 | 5.6 | 1.9E-03 | 4.5E-03 | [ANXA4, ANXA5, DDX3X, HSPA1A, PSMD1, PSMD14, THBS1] |
| GO:0060548 | negative regulation of cell death | 8 | 6.0 | 2.1E-03 | 5.0E-03 | [ANXA4, ANXA5, ARHGEF2, DDX3X, HSPA1A, PSMD1, PSMD14, THBS1] |
| GO:0008285 | negative regulation of cell proliferation | 4 | 5.1 | 2.2E-04 | 6.1E-04 | [GARS, HSPA1A, REXO2, THBS1] |
| GO:0042127 | regulation of cell proliferation | 7 | 4.2 | 1.2E-17 | 7.7E-17 | [ARHGEF2, CAPNS1, GARS, HSPA1A, REXO2, SHMT2, THBS1] |
| GO:0051726 | regulation of cell cycle | 6 | 4.9 | 4.4E-05 | 1.3E-04 | [DDX3X, HSPA2, PSMD1, PSMD14, REXO2, THBS1] |
| GO:0048522 | positive regulation of cellular process | 19 | 4.0 | 1.3E-46 | 1.3E-45 | [ARHGEF2, C14orf166, CAP1, CAPNS1, DDX3X, DNAJC3, EHD2, GARS, HSPA1A, HSPA2, NMT1, NQO1, PSMD1, PSMD14, REXO2, RPS6, SHMT2, THBS1, TWF2] |
| GO:0048523 | negative regulation of cellular process | 16 | 3.5 | 1.2E-52 | 1.5E-51 | [ANXA4, ANXA5, AP2A1, AP2A2, ARHGEF2, BASP1, DDX3X, DNAJC3, GARS, HSPA1A, PSMD1, PSMD14, REXO2, SRSF9, THBS1, TWF2] |
| GO:0006935 | chemotaxis | 5 | 5.2 | 2.6E-04 | 6.8E-04 | [AP2A1, AP2A2, CAP1, MYL6, THBS1] |
| GO:0006974 | cellular response to DNA damage stimulus | 4 | 3.5 | 7.2E-06 | 2.6E-05 | [DDX1, PSMD1, PSMD14, REXO2] |
| GO:0009725 | response to hormone | 4 | 3.8 | 3.7E-06 | 1.4E-05 | [CALM1, NQO1, RPS6, THBS1] |
| GO:0010243 | response to organonitrogen compound | 4 | 3.6 | 1.6E-05 | 5.1E-05 | [ARHGEF2, CALM1, GARS, RPS6] |
| GO:0014070 | response to organic cyclic compound | 5 | 5.3 | 8.0E-04 | 2.0E-03 | [CALM1, DDX1, GARS, NQO1, THBS1] |
| GO:0033554 | cellular response to stress | 8 | 3.3 | 3.3E-18 | 2.2E-17 | [ARHGEF2, DDX1, DDX3X, DNAJC3, PSMD1, PSMD14, REXO2, THBS1] |
| GO:0033993 | response to lipid | 4 | 5.6 | 2.1E-04 | 5.9E-04 | [CALM1, NQO1, THBS1, TWF2] |
| GO:0042060 | wound healing | 5 | 4.3 | 1.4E-04 | 4.0E-04 | [ANXA5, CAP1, EHD2, GARS, THBS1] |
| GO:0070887 | cellular response to chemical stimulus | 11 | 3.4 | 6.0E-59 | 1.3E-57 | [AP2A1, AP2A2, ARHGEF2, CAP1, DDX3X, DNAJC3, GARS, NQO1, RPS6, THBS1, TWF2] |
| GO:0071310 | cellular response to organic substance | 9 | 3.5 | 1.8E-39 | 1.7E-38 | [AP2A1, AP2A2, ARHGEF2, CAP1, DNAJC3, GARS, RPS6, THBS1, TWF2] |
| GO:0071363 | cellular response to growth factor stimulus | 6 | 6.7 | 1.7E-03 | 4.0E-03 | [AP2A1, AP2A2, ARHGEF2, GARS, THBS1, TWF2] |
| GO:0080134 | regulation of response to stress | 5 | 3.8 | 7.0E-08 | 3.3E-07 | [AP2A1, AP2A2, ARHGEF2, CAP1, THBS1] |

**Table S11 Significantly enriched biological processes of the genes identified in the miRNA/mRNA network.** Enrichment analysis revealed biological processes such as ‘signal transduction’, ‘cell death’, ‘RNA and protein metabolism’, ‘cell communication’, ‘response to stimuli’, ‘development’ and ‘RNA and protein biosynthesis’ as highly enriched.

| GO ID | Term | p-value | Count | Size |
| --- | --- | --- | --- | --- |
| GO:0065007 | biological regulation | 6.44E-11 | 116 | 8347 |
| GO:0050794 | regulation of cellular process | 4.16E-08 | 64 | 4921 |
| GO:0051248 | negative regulation of protein metabolic process | 4.77E-06 | 13 | 293 |
| GO:0035556 | intracellular signal transduction | 2.25E-05 | 35 | 1785 |
| GO:0016265 | death | 5.27E-05 | 32 | 1627 |
| GO:0080090 | regulation of primary metabolic process | 6.64E-05 | 60 | 4104 |
| GO:0030505 | inorganic diphosphate transport | 9.11E-05 | 2 | 2 |
| GO:0048519 | negative regulation of biological process | 1.56E-04 | 33 | 1982 |
| GO:0009890 | negative regulation of biosynthetic process | 2.07E-04 | 21 | 931 |
| GO:0071375 | cellular response to peptide hormone stimulus | 3.07E-04 | 10 | 274 |
| GO:0051252 | regulation of RNA metabolic process | 3.41E-04 | 45 | 2889 |
| GO:0031326 | regulation of cellular biosynthetic process | 3.65E-04 | 47 | 3177 |
| GO:0010556 | regulation of macromolecule biosynthetic process | 4.42E-04 | 44 | 2971 |
| GO:0031324 | negative regulation of cellular metabolic process | 4.55E-04 | 8 | 206 |
| GO:0032868 | response to insulin stimulus | 6.22E-04 | 9 | 247 |
| GO:0012501 | programmed cell death | 7.50E-04 | 27 | 1480 |
| GO:0033629 | negative regulation of cell adhesion mediated by integrin | 8.94E-04 | 2 | 5 |
| GO:0038034 | signal transduction in absence of ligand | 8.94E-04 | 2 | 5 |
| GO:0043951 | negative regulation of cAMP-mediated signaling | 8.94E-04 | 2 | 5 |
| GO:0007173 | epidermal growth factor receptor signaling pathway | 9.10E-04 | 7 | 161 |
| GO:0010648 | negative regulation of cell communication | 9.14E-04 | 14 | 561 |
| GO:0034329 | cell junction assembly | 9.44E-04 | 7 | 162 |
| GO:0032088 | negative regulation of NF-kappaB transcription factor activity | 9.62E-04 | 4 | 46 |
| GO:0046627 | negative regulation of insulin receptor signaling pathway | 1.01E-03 | 3 | 21 |
| GO:0023057 | negative regulation of signaling | 1.01E-03 | 14 | 563 |
| GO:0051171 | regulation of nitrogen compound metabolic process | 1.02E-03 | 49 | 3395 |
| GO:2000113 | negative regulation of cellular macromolecule biosynthetic process | 1.12E-03 | 5 | 87 |
| GO:0031400 | negative regulation of protein modication process | 1.14E-03 | 5 | 84 |
| GO:0048662 | negative regulation of smooth muscle cell proliferation | 1.16E-03 | 3 | 22 |
| GO:0071495 | cellular response to endogenous stimulus | 1.40E-03 | 13 | 521 |
| GO:0032774 | RNA biosynthetic process | 1.78E-03 | 44 | 3024 |
| GO:0007154 | cell communication | 1.81E-03 | 50 | 3886 |
| GO:0007167 | enzyme linked receptor protein signaling pathway | 1.83E-03 | 17 | 807 |
| GO:0033002 | muscle cell proliferation | 2.18E-03 | 5 | 95 |
| GO:0043412 | macromolecule modication | 2.39E-03 | 36 | 2359 |
| GO:1900076 | regulation of cellular response to insulin stimulus | 2.62E-03 | 3 | 29 |
| GO:0045321 | leukocyte activation | 3.21E-03 | 12 | 502 |
| GO:0048585 | negative regulation of response to stimulus | 3.32E-03 | 14 | 640 |
| GO:0032268 | regulation of cellular protein metabolic process | 3.63E-03 | 18 | 968 |
| GO:0045892 | negative regulation of transcription, DNA-dependent | 3.92E-03 | 15 | 723 |
| GO:0071841 | cellular component organization or biogenesis at cellular level | 3.95E-03 | 43 | 3056 |
| GO:0010741 | negative regulation of intracellular protein kinase cascade | 4.11E-03 | 5 | 110 |
| GO:0051402 | neuron apoptosis | 4.27E-03 | 6 | 159 |
| GO:0051716 | cellular response to stimulus | 4.41E-03 | 34 | 2576 |
| GO:0070830 | tight junction assembly | 4.50E-03 | 3 | 35 |
| GO:0007289 | spermatid nucleus differentiation | 4.73E-03 | 2 | 11 |
| GO:0036092 | phosphatidylinositol-3-phosphate biosynthetic process | 4.73E-03 | 2 | 11 |
| GO:0033522 | histone H2A ubiquitination | 5.64E-03 | 2 | 12 |
| GO:0010605 | negative regulation of macromolecule metabolic process | 5.66E-03 | 15 | 815 |
| GO:0009725 | response to hormone stimulus | 6.33E-03 | 14 | 689 |
| GO:2000027 | regulation of organ morphogenesis | 6.35E-03 | 5 | 122 |
| GO:0060491 | regulation of cell projection assembly | 7.51E-03 | 3 | 42 |
| GO:0031175 | neuron projection development | 7.65E-03 | 13 | 632 |
| GO:0045934 | negative regulation of nucleobase-containing compound metabolic process | 7.66E-03 | 16 | 854 |
| GO:0006607 | NLS-bearing substrate import into nucleus | 7.68E-03 | 2 | 14 |
| GO:0031146 | SCF-dependent proteasomal ubiquitin-dependent protein catabolic process | 7.68E-03 | 2 | 14 |
| GO:0050678 | regulation of epithelial cell proliferation | 7.94E-03 | 6 | 181 |
| GO:0044260 | cellular macromolecule metabolic process | 8.01E-03 | 74 | 6203 |
| GO:0006801 | superoxide metabolic process | 8.02E-03 | 3 | 43 |
| GO:0018107 | peptidyl-threonine phosphorylation | 8.02E-03 | 3 | 43 |
